# Proceedings of the 27^th^ Annual Meeting of the Portuguese Society of Human Genetics (SPGH – *Sociedade Portuguesa de Genética Humana*) Lisbon, 23 -25 November 2023

**DOI:** 10.1097/MD.0000000000039478

**Published:** 2025-01-24

**Figure:**
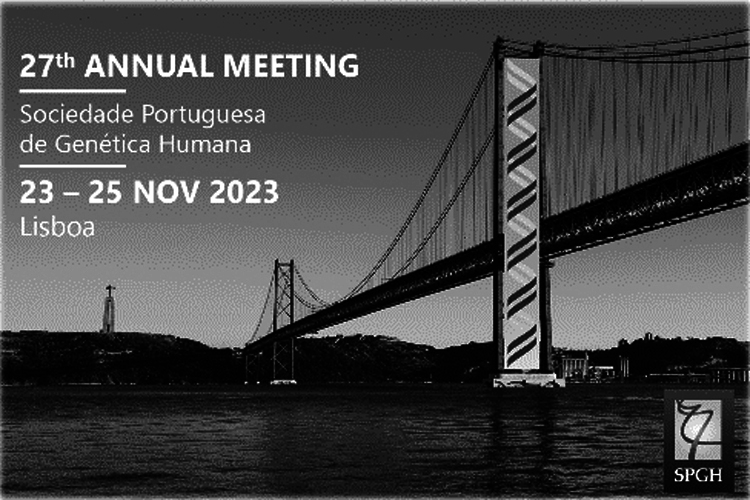


Dear colleagues,

Dear participants,

Dear invited speakers,

The Board of Directors of the Portuguese Society of Human Genetics (SPGH) welcomes you to the 27th Annual Meeting, in Lisbon.

This year, we are very pleased to be able to continue the biggest event in the area of Human Genetics in Portugal, with a very high number of 300 participants and 114 scientific abstracts submitted. Likewise, the support from companies linked to the sector has been notable.

We hope that this year’s scientific program will once again meet your expectations and allow for the much-desired interaction and dialogue between the various professionals in the sector: medical geneticists, researchers, specialists in laboratory genetics, and other professionals with an interest in human genetics. May it once again be a bridge, as suggested by the logo chosen for this meeting, between young and experienced members, between different disciplines of knowledge, and between all those who contribute to the practice of human genetics in this country and in Europe.

We would like to thank, from the outset, everyone who contributed and committed themselves to preparing this meeting: the members of the scientific committee, supported by external collaborators, the members of the bioethics committee, the local collaborators who provide the logistics behind the scenes, and the companies whose support is essential to ensure the meeting´s infrastructure.

We also owe special thanks to the guest speakers who agreed to come and share their results and scientific advances, and are a decisive element of the annual meeting program.

We hope that this annual meeting in Lisbon will be another important milestone in the history of SPGH, and that it will enrich all participants with updated knowledge, new or reinforced professional contacts, and private moments of conviviality.

The effective Management of 2023

Peter Jordan

José Carlos Ferreira

Claudia Oliveira

## Oral presentations

### Basic Research

#### OP1 – PERMISSION TO PUBLISH NOT GRANTED BY THE AUTHORS

#### OP2 - DISSECTING THE ROLE OF MICRORNAS IN EFFECTOR VERSUS REGULATORY CD4+ T CELL DIFFERENTIATION DURING (AUTO)IMMUNE RESPONSES IN VIVO

Carolina Cunha^1^, Paula Vargas Romero^1^, Daniel Inácio^1^, Ana Teresa Pais^1^, Catarina Pelicano^1^, Daniel Sobral^2^, Marina Costa^1^, Sofia Mensurado^1^, Natacha Gonçalves Sousa^1^, Pedro Papotto^1^, Francisco Enguita^1^, Anita Q. Gomes^1,3,*^, Bruno Silva-Santos^1,*^

^1^*Instituto de Medicina Molecular João Lobo Antunes, Faculdade de Medicina, Universidade de Lisboa, Lisbon, Portugal.* ^2^*Instituto Nacional de Saúde Dr. Ricardo Jorge, Lisboa, Lisbon, Portugal.* ^3^*H&TRC Health & Technology Research Center, ESTeSL-Escola Superior de Tecnologia da Saúde, Instituto Politécnico de Lisboa, Lisbon, Portugal.* ^*^ *These authors contributed equally*

**Introduction:** MicroRNAs (miRNAs) are small non-coding RNAs that negatively regulate gene expression at the post-transcriptional level. They have been implicated in the regulation of the differentiation and function of CD4+ T cell subsets, key players in host defense against pathogens, but also responsible for immune-mediated diseases depending on the correct vs incorrect balance, respectively, between pro-inflammatory effector CD4+T cells, including the IFN-γ-producers T helper 1 (Th)1 and the IL-17-producers Th17 cells, and anti-inflammatory regulatory T cells (Treg). While individual miRNAs were found to regulate the differentiation of specific CD4+ T cell populations, an approach based on in vivo responses is still missing and is key to understand how miRNA networks control this balance in pathophysiology.

**Methodology:** We have established a triple reporter mouse for Ifng, Il17 and Foxp3, and subjected it to experimental autoimmune encephalomyelitis (EAE). We performed miRNA-seq analysis on Th1, Th17 and Treg cells isolated from the spleen and lymph nodes (LNs) at peak-plateau stage to identified miRNA candidates specifically expressed in one of the cell populations. We have in vivo modulated their expression levels using antagomiRs and observed the course of EAE progression and characterised their upstream regulation in vitro in either Th1 or Th17 differentiation conditions.

**Results:** The miRNA-seq data has allowed the identification of 110 miRNAs differentially expressed between effector (Th1 and Th17) and regulatory (Treg) subsets. From those, 9 were specifically upregulated in one population versus the others. In vivo miRNA modulation showed that silencing miR-122 precipitated the onset of EAE, whereas overexpressing miR-1247 decreased the severity of the disease. Cytokine-regulated miR-1247 and miR-122 expression levels inversely associated with pathogenic signatures of Th1 and Th17 cells between lymphoid and central nervous systems.

**Discussion:** Our results suggest that miR-122 and miR-1247 act as peripheral brakes to CD4+ T cell pathogenicity that are overruled in the inflamed target organ. These findings may have important implications for autoimmune diseases.

#### OP3 - HE CDH1 REGULATORY BLUEPRINT: SHAPING HEREDITARY DIFFUSE GASTRIC CANCER

Celina São José^1,2,3^, Marta Ferreira^1,4^, Francisco Almeida^1^, José Pelaez-Garcia^1,3^, Ana André^1^, Janine Senz^5^, Pardeep Kourah^5^, Lilian Cordova^5^, Fiona Puntieri^2^, Juliane Glasser^2^, Kasmintan Schrader^5^, Stefan Mundlos^2,6,7^, David Huntsman^5,8,9^, Carla Oliveira^1^

^1^ *Instituto de Investigação e Inovação em Saúde, University of Porto, Porto, Portugal* ^2^ *Max Planck Institute for Molecular Genetics, Berlin, Germany.* ^3^ *Doctoral Programme in Biomedicine, Faculty of medicine, University of Porto, Porto, Portugal.* ^4^ *Dept. Computer Science, Faculty of Science, University of Porto.* ^5^ *Department of Pathology and Laboratory Medicine, University of British Columbia, Vancouver, British Columbia, Canada.* ^6^ *Institute of Medical and Human Genetics, Charité Universitätsmedizin Berlin, Berlin, Germany.* ^7^ *Berlin-Brandenburg Center for Regenerative Therapies (BCRT), Charité Universitätsmedizin Berlin, Berlin, Germany.* ^8^ *Centre for Translational and Applied Genomics (CTAG), BC Cancer Agency, Vancouver, British Columbia, Canada.* ^9^ *Genetic Pathology Evaluation Centre, University of British Columbia and Vancouver General.* ^10^ *Faculty of Medicine of the University of Porto, Porto, Portugal.*

**Background:** Hereditary diffuse gastric cancer (HDGC) is caused by CDH1 or CTNNA1 germline inactivation, which explains <40% of cases. Missing heritability prevents proper management and disease prevention in HDGC-suspected families. We hypothesized that defects in CDH1-regulatory elements contribute to HDGC missing heritability.

**Methods:** We called single-nucleotide/copy-number variants (SNV/CNV) from 19 HDGC probands whole-genome sequencing data, performed gene-ontology analysis, 4C-seq and ATAC-seq in normal stomach epithelia and CRISPR-Cas9, RT-PCR and flow cytometry in cell lines.

**Results:** Besides a MLH1 2,7kb deletion, no relevant coding variants were found in gastrointestinal cancer-associated genes. From stomach ATAC and 4C-seq data, we extracted 46.249 accessible chromatin regions and 370 promoter CDH1 interactor-regions overlapping 1.882 rare CNVs. CNVs overlapping CDH1 promoter interactions revealed a 39 bp-intergenic deletion downstream of CDH1, and a 20kb CDH3 deletion. CRISPR-Cas9 mimicking each deletion (homozygous) triggered 30% and 50% CDH1 mRNA downregulation, respectively for the 39 bp-intergenic and CDH3 CNVs. These sequences acted as enhancers in E-cadherin expressing tissues during development of mouse embryos. The pattern of deleted accessible chromatin regions per patient revealed an HDGC-group bearing downregulated immune-associated pathways.

**Conclusion:** We identified two novel hypomorphic CNVs in CDH1-regulatory regions contributing for CDH1 downregulation, and a MLH1 deletion in a family without classical Lynch Syndrome, that may explain the missing heritability in three HDGC-suspected families. A gastric-specific regulatory element within the MLH1 CNV support predisposition to gastric cancer in this family. Germline CNVs in stomach-specific regulatory regions may predispose to a immune-suppressive phenotype favourable for HDGC development.

Grant references: PTDC/BTM-TEC/30164/2017, PTDC/BTM-TEC/6706/2020, 22184, SFRH/BD/140796/2018.

#### OP4 - USING HRDETECT TO TUNE THE CLASSIFICATION OF VARIANTS OF UNCERTAIN SIGNIFICANCE IN A PAN-CANCER COHORT OF 10,675 SAMPLES

Orlando P. Rodrigues^1,2,3^, Lucia Chmelova^1,2^, Giuseppe Rinaldi^1,2^, James Whitworth^2^, Ramsay Bowden^2^, Helen Davies^1,2^, Gene Koh^1,2^, Serena Nik-Zainal^1,2^

^1^*Early Cancer Institute, Department of Oncology, University of Cambridge, Cambridge CB2 0XZ, UK.* ^2^*Academic Department of Medical Genetics, School of Clinical Medicine, University of Cambridge, Cambridge CB2 9NB, UK.* ^3^*Medical Genetics Department, Hospital Pediátrico do Centro Hospitalar e Universitário de Coimbra*

**Introduction:** Homologous recombination deficiency (HRd) is crucial to the development and treatment of various cancers. We explore if HRDetect – a clinical classifier that uses Whole Genome Sequencing (WGS) data to predict HRd – could be used in conjunction with ACMG criteria to aid the classification of variants of unknown significance (VUS) in HR-related genes. We also survey the HRd status in cancers with variants of known significance.

**Method:** We focused on VUS in BRCA1, BRCA2, PALB2, RAD51, and RAD51D across 10,675 tumor-germline pairs within the Genomics England 100,000 genomes project covering 16 tumor types. In total, we identified 1,580 samples (14,8%) with 2,329 small nucleotide variants (881 germline vs. 1,448 somatic). Clinical significance of these variants was annotated using ClinVar.

**Results:** Our findings suggest the potential reclassification of six VUS as pathogenic, implicating their role in HRd due to the presence of HRd-associated mutational signatures. Additionally, we found that not all cancers with germline pathogenic and likely pathogenic (P/LP) variants in HR genes had high HRDetect scores. Among 48 breast cancers (BCs) with germline BRCA2 P/LP variants, 44 (91,7%) were HRDetect-high (score>0.5), 4 (8,3%) were HRDetect-low (score≤0.5). Likewise, somatic events were inconsistent: among 17 BCs with somatic BRCA2 (P/LP) variants, 13 (76,5%) were HRDetect-high, 4 (23,6%) were HRDetect-low.

**Discussion:** Our findings highlight the promise of integrating HRDetect score into ACMG criteria to inform VUS classification. While our analysis was limited by sample size, it underscores the need for further research to comprehensively assess the added value of HRDetect in VUS clinical interpretation. In clinical practice, tumour fraction and/or variant allele fraction (VAF) are commonly used to distinguish passenger from driver events. Mainstream sequencing approach based on targeted panels often precludes accurate tumour purity information. While higher VAF generally correlates with high HRDetect score status, we have found HRDetect-high samples with variants present at VAF as low as 0.1, raising the question as to what VAF threshold is optimal. Moreover, carriers of germline pathogenic variants in HR genes may develop sporadic cancers driven by other driver events (i.e., HRDetect-low cases), limiting the efficacy of targeted therapies. This work presents the challenges inherent in making evidence-based clinical decisions in genomic testing.

**DOI**: Serena Nik-Zainal holds patents on HRDetect (PCT/EP2017/060294)

#### OP5 - A PROMISING SUBSTRATE REDUCTION APPROACH FOR A RARE GENETIC DISEASE: MUCOPOLYSACCHARIDOSES TYPE III

Juliana I. Santos^1,2,3,4^, Mariana Gonçalves^1,3,4,5^, Sofia Carvalho^1,3,4^, Liliana Matos^1,3,4^, Paulo Gaspar^6^, Maria João Prata^2,7^, Maria Francisca Coutinho^1,3,4^, Sandra Alves^1,3,4^

^1^ *Research and Development Unit, Department of Human Genetics, INSA, Porto, Portugal;* ^2^ *Biology Department, Faculty of Sciences, University of Porto, Portugal;* ^3^ *Centre for the Study of Animal Science, CECA-ICETA, University of Porto, Porto, Portugal;* ^4^ *Associate Laboratory for Animal and Veterinary Sciences (AL4AnimalS);* ^5^ *CITAB – Centre for the Research and Technology of Agro-Environmental and Biological Sciences, Vila Real, Portugal;* ^6^ *Newborn Screening, Metabolism and Genetics Unit, Department of Human Genetics, INSA, Porto, Portugal;* ^7^ *i3S – Health Research and Innovation Institute, University of Porto, Portugal.*

**Introduction:** The term RNA therapy has gained more and more attention over the last years and has reignited research interest since the COVID-19 pandemic, due to the effectiveness of mRNA-based vaccines. In fact, recent advances in the production, modification and cellular delivery of RNA molecules are facilitating the expansion of RNA-based therapeutics. That is why many RNA drugs are approved for clinical practice and many others are in clinical trials, targeting diseases that cannot be treated by other conventional drugs.

We have been addressing the potential antisense oligonucleotides (ASOs) and small interfering RNAs (siRNAs) to correct/ameliorate the sub-cellular phenotype of a particular subset of genetic diseases caused by lysosomal dysfunction. Our goal is to reduce the accumulation of the non-degraded substrate (in this case, the glycosaminoglycan (GAG) heparan sulphate) in cells and tissues of Mucopolysaccharidoses type III patients.

**Methodology:** Different ASOs (steric block splicing switching ASOs and gapmer ASOs) and siRNAs, targeting the XYLT1 gene (involved in GAGs biosynthesis), were designed and transfected in MPS III patients’ fibroblasts. The results at cDNA and mRNA levels were evaluated by RT-PCR and qRT-PCR, respectively. Their effect on protein will be evaluated through western blot assays and the GAGs accumulation will be addressed by LC-MS/MS.

**Results:** We observed the expected splicing modulation after the transfection of the steric block splicing switching ASOs, leading to the skipping of XYLT1 target exons. Similarly, the XYLT1 mRNA downregulation was obtained after the transfection of gapmer ASOs and siRNAs (a range of 4 to 15 times less expression compared to non-transfected fibroblasts). The effects on protein level and GAGs accumulation are currently being addressed.

**Discussion:** Overall, this approach confirmed the ability of specific ASOs to modulate RNA splicing as well as the capacity of gapmers ASOs and siRNAs to promote a reduction in mRNA levels of the target gene, thus paving the way for future studies on the therapeutic potential of this strategy.

**Acknowledgements:** This work is partially supported by FCT (2022.04667.PTDC), SPDM (2019DGH1629/SPDM2018I&D and 2020DGH1834) and SCF (2019DGH1656/SCF2019I&D).

### Clinical Research

#### OP1 - THE SOMATIC LANDSCAPE OF DIFFUSE GASTRIC CANCERS OCCURRING IN CTNNA1 GERMLINE VARIANT CARRIER PATIENTS

Silvana Lobo^1,2,3^, Alexandre Dias^1,2,3^, Marta Ferreira^1,2,4^, Nelson Martins^1,2,5^, Rita Barbosa-Matos^1,2,3^, João Fonseca^1,2,5^, José Garcia-Pelaez^1,2,5^, Chrystelle Colas^6,7^, Robert Hüneburg^7,8,9^, Jacob Nattermann^7,8,9^, Lise Boussemart^10,11^, Liselotte P. van Hest^12^, Leticia Moreira^13,14,15^, Carolyn Horton^16^, Dana Farengo Clark^17^, Sigrid Tinschert^18,19^, Lisa Golmard^6,7^, Isabel Spier^7,8,20^, Adrià López-Fernández^7,21^, Daniela Oliveira^22,23,24^, Magali Svrcek^25,26^, Pierre Bourgoin^26^, Helene Delhomelle^6,7^, Jeremy Davis^27^, Birthe Zäncker^28^, Conxi Lázaro^7,29^, Joana Guerra^3,30^, Manuel R Teixeira^3,7,30^, Kasmintan A Schrader^31,32^, Paul Mansfield^33^, Verena Steinke-Lange^7,34,35^, Sérgio Sousa^22,23,24^, Manuela Baptista^36^, Stefan Aretz^7,8,20^, Judith Balmaña^7,21^, Melyssa Aronson^37^, Augusto Antoniazzi^38^, Edenir Inêz Palmero^39,40^, Lizet E van der Kolk^41^, Annemieke Cats^42^, Jolanda M van Dieren^42^, Sergi Castellví-Bel^13,14,15^, Bryson W. Katona^17^, Rachid Karam^16^, Florence Coulet^43^, Patrick R. Benusiglio^44,45^, Paulo S. Pereira^1,46^, Carla Oliveira^1,2,7,5,*^

^1^*i3S – Instituto de Investigação e Inovação em Saúde, Porto, Portugal;* ^2^*IPATIMUP – Institute of Molecular Pathology and Immunology of the University of Porto, Portugal;* ^3^*Institute of Biomedical Sciences Abel Salazar, University of Porto, Portugal;* ^4^*Faculty of Science, University of Porto, Portugal;* ^5^*Faculty of Medicine, University of Porto, Portugal;* ^6^*Department of Genetics, Institut Curie, University Paris Sciences Lettres, France;* ^7^*Full Member of the European Reference Network on Genetic Tumor Risk Syndromes (ERN GENTURIS) – Project ID No 739547;* ^8^*National Center for Hereditary Tumor Syndromes, University Hospital Bonn, Germany;* ^9^*Department of Internal Medicine I, University Hospital Bonn, Germany;* ^10^*Nantes Université, Univ Angers, CHU Nantes, INSERM, Immunology and New Concepts in ImmunoTherapy, INCIT, France;* ^11^*Department of Dermatology, Nantes University Hospital, France;* ^12^*Amsterdam UMC, Vrije Universiteit Amsterdam, Department of Clinical Genetics, The Netherlands;* ^13^*Department of Gastroenterology, Hospital Clínic Barcelona, Spain;* ^14^*Centro de Investigación Biomédica en Red en Enfermedades Hepáticas y Digestivas (CIBEREHD), Spain;* ^15^*Institut d’Investigacions Biomèdiques August Pi i Sunyer (IDIBAPS), University of Barcelona, Barcelona, Spain;* ^16^*Ambry Genetics, Aliso Viejo, CA, USA;* ^17^*Division of Gastroenterology and Hepatology, University of Pennsylvania Perelman School of Medicine, Philadelphia, PA, USA;* ^18^*Division of Human Genetics, Medical University Innsbruck, Austria;* ^19^*IFLb Laboratoriumsmedizin Berlin GmbH, Windscheidstraße, Berlin-Charlottenburg, Germany;* ^20^*Institute of Human Genetics, Medical Faculty, University of Bonn, Germany;* ^21^*Hereditary Cancer Group, Medical Oncology Department Hospital Vall d’Hebron, and Vall d’Hebron Institute of Oncology, Barcelona, Spain;* ^22^*Medical Genetics Unit, Paediatric Hospital, Coimbra Hospital and University Centre, Portugal;* ^23^*University Clinic of Genetics, Faculty of Medicine, University of Coimbra, Portugal;* ^24^*Clinical Academic Centre of Coimbra, Portugal;* ^25^*Sorbonne Université, UPMC Univ Paris 06, INSERM, UMRS 938, SIRIC CURAMUS, Equipe Instabilité Des Microsatellites Et Cancer, Equipe Labellisée Par La Ligue Contre Le Cancer, Centre de Recherche Saint Antoine, Paris, France;* ^26^*Sorbonne Université, Laboratoire D’anatomie Et Cytologie Pathologiques, Hôpital Saint-Antoine, AP-HP, Paris, France;* ^27^*Surgical Oncology Program, National Cancer Institute, National Institutes of Health, Bethesda, MD, USA;* ^28^*Institut für Klinische Genetik, Universitätsklinikum Carl Gustav Carus Dresden, Dresden, Germany;* ^29^*Hereditary Cancer Program, Catalan Institute of Oncology, Bellvitge Institute for Biomedical Research, Barcelona, Spain; Centro de Investigación Biomédica en Red de Cáncer (CIBERONC), Madrid, Spain;* ^30^*Department of Laboratory Genetics, Portuguese Oncology Institute of Porto, Portugal;* ^31^*Department of Medical Genetics, Faculty of Medicine, University of British Columbia, Vancouver, BC, Canada;* ^32^*Department of Molecular Oncology, BC Cancer, Vancouver, BC, Canada;* ^33^*The University of Texas MD Anderson Cancer Center, Houston, USA;* ^34^*Medizinische Klinik und Poliklinik IV, Klinikum der Universität München, Germany;* ^35^*MGZ – Medizinisch Genetisches Zentrum, Munich, Germany;* ^36^*Centro Hospitalar Universitário São João, Porto, Portugal;* ^37^*Zane Cohen Centre, Sinai Health System, Toronto, Canada;* ^38^*Cancer Genetics Departament, Barretos Cancer Hospital, Brazil;* ^39^*Molecular Oncology Research Center, Barretos Cancer Hospital, Brazil;* ^40^*Department of Genetics, National Cancer Institute, Brazil;* ^41^*Department of Clinical Genetics, Netherlands Cancer Institute, Amsterdam, The Netherlands;* ^42^*Department of Gastrointestinal Oncology, Netherlands Cancer Institute, Amsterdam, The Netherlands;* ^43^*Unité fonctionnelle d’Onco-angiogénétique et génomique des tumeurs solides, Département de Génétique médicale, Hôpital Pitié-Salpêtrière, AP-HP, Sorbonne Université, Paris, France;* ^44^*Unité fonctionnelle d’Oncogénétique clinique, Département de Génétique, Groupe Hospitalier Pitié-Salpêtrière, AP-HP, Sorbonne Université, Paris, France;* ^45^*Chirurgie générale et digestive, Hôpital Saint-Antoine, AP-HP, Sorbonne Université, Paris, France;* ^46^*IBMC – Instituto de Biologia Molecular e Celular, Porto, Portugal;* ^*^Corresponding author (carlaol@i3s.up.pt)

Germline pathogenic variants, including rare copy number variants (CNVs), in cancer predisposing genes (CPG) cause genetic tumour risk syndromes (TRS). TRS-causative variants can be clinically actionable and lead to intensive surveillance and/or risk reducing surgery that improve morbidity and mortality. Regrettably, causative and actionable variants cannot be found for all TRS-suspected individuals. While for SNV-calling specificity/sensitivity is almost 100%, CNV detection in exome-data remains challenging. We hypothesized that pathogenic CNVs in CPG may solve some of the yet unexplained, but clinically suspected gastrointestinal TRS-cases. The ERN-GENTURIS/SOLVE-RD project, re-analyzed exomes from 293 unsolved TRS-cases: adenomatous polyposis (AP; n=105), hyperplastic polyposis (HP; n=98), hereditary gastric cancer (HGC; n=83) and hereditary colorectal cancer (hCRC; n=7). CNVs were called with four different variant callers (ClinCNV, ExomeDepth, Conifer, VarGenius). 341 CNVs filtered from 229 CPGs were prioritized for their involvement in GI tumours, quality and calling by >1 caller. High-quality and/or ‘multiple-called’ CNVs were evaluated using IGV and focused paired-end mapping/split-read analysis. Eight CNVs (6-del; 2-dup), 3 ‘multiple-called’, were found in 11/293 TRS-cases, sometimes in cases with an atypical phenotype. A CDH1 deletion, validated by MLPA, is found in 4 HGC-relatives. Supported by split-reads/paired-end mapping, it was considered an actionable diagnosis (4.8% among HGC cases). A deletion affecting PALB2 in 1/83 (1.2%) HGC cases was validated by qPCR. In an AP case, the last fraction of the APC and the beginning of the SRP19 gene was found deleted and supported by split-reads/paired-end mapping. Afterwards, validated by MLPA. Using a different approach, looking for ‘multiple called’ CNVs in CPG not directly associated with GI-TRS, a big deletion in SLX4, previously associated with sporadic CRC, was also validated by qPCR, solving 1/98 (1%) HP cases. Altogether, this approach delivered a potential diagnosis in at least 2.3% of unsolved GI TRS-cases.

#### OP2 - MEDICALLY ACTIONABLE SECONDARY FINDINGS FROM WHOLE EXOME SEQUENCING (WES) DATA IN A SAMPLE OF 3,972 INDIVIDUALS

Mafalda Melo^1,2^, Paulo Silva^2,3^, Mariana Ribeiro^2,4^, Susana Valente^2,4^, Filipe Alves^2^, Ana Coutinho^2^, Margarida Venâncio^1^, Jorge Sequeiros^2,3,5^, João Parente Freixo^2,3^, Diana Antunes^1^, Jorge Oliveira^2,3^

^1^ *Unidade de Genética Médica, Centro Hospitalar Universitário de Lisboa Central (CHULC), Lisboa, Portugal;* ^2^ *Centro de Genética Preditiva e Preventiva (CGPP), Instituto de Biologia Molecular e Celular (IBMC), Instituto de Investigação e Inovação em Saúde (i3S), Universidade do Porto, Portugal;* ^3^ *UnIGENe, Instituto de Investigação e Inovação em Saúde (i3S), Universidade do Porto, Portugal;* ^4^ *Departamento de Ciências Médicas, Universidade de Aveiro;* ^5^ *ICBAS – Instituto de Ciências Biomédicas Abel Salazar, Universidade do Porto, Porto, Portugal.*

**Introduction:** The application of whole-exome sequencing (WES) for diagnostic purposes has the potential to unravel secondary findings unrelated with the primary purpose of the genetic test. These are of high clinical utility and comprise disease-causing variants in genes related to life-threatening and/or preventable diseases. Clarifying the allelic frequencies of disease-causing variants in specific populations is a crucial step in implementing genomic medicine on a large scale. However, this data is currently unavailable for the Portuguese population.

**Methodology:** We analyzed medically actionable variants in 81 genes from the ACMG list (v3.2) of actionable loci using WES data obtained from a large laboratory cohort comprising 12,167 samples which was tentatively resampled to be representative of the Portuguese population (3,972 individuals).

**Results:** We identified a total of 643 (230 distinct) pathogenic (PAT) or likely pathogenic (L-PAT) variants across 49 ACMG genes, with an overall frequency of 8.1%.

Cardiovascular diseases group was the most predominant with actionable results (2.6% of the individuals), being the most frequent PAT/L-PAT variants found in genes related to cardiomyopathies (TTN and MYH7), dyslipidemias (LDLR), heart conduction disorders (KCNQ1), and aortopathies (FBN1). The second represented group of disorders was cancer predisposition (1.7%) and included variants in genes related to colon cancer (PMS2, MSH6 and MSH2) and breast cancer susceptibility (BRCA2, PALB2, and BRCA1). Finally, we also observed actionable variants related with miscellaneous disorders (1.0%) and metabolic disorders (0.1%), such as hereditary transthyretin amyloidosis (TTR), malignant hyperthermia (RYR1), and the X-linked Fabry disease (GLA).

**Discussion:** Overall, our results suggest that medically actionable findings can be identified in approximately 5.4% of the Portuguese population. To the best of our knowledge, this is the first study estimating medically actionable findings in our country. These results provide valuable information for patients, practitioners, and stakeholders involved in genomic medicine. Ultimately, they may support the implementation of an organized genomic opportunistic screening program, tailored to improve healthcare in Portugal.

#### OP3 - WHOLE-EXOME SEQUENCING AT A PORTUGUESE MEDICAL GENETICS CENTRE: 883 CASES OVER AN 8-YEAR PERIOD

Sara Pinho, Raquel Gouveia Silva, Catarina Macedo, Mariana Neves, Marta Soares, André Travessa, Mariana Soeiro e Sá, Márcia Rodrigues, Patrícia Dias, Oana Moldovan, Juliette Dupont, Ana Berta Sousa

Serviço de Genética Médica, Departamento de Pediatria, Centro Hospitalar Universitário Lisboa Norte, Lisbon, Portugal

**Introduction:** Whole-exome sequencing (WES) has become a fundamental diagnostic tool for patients with a suspected monogenic disorder, especially in complex cases with unknown clinical diagnosis. This study analyzed the patient characteristics and WES results obtained since we first began using this test.

**Methodology:** We performed a retrospective analysis of 883 WES reports, consecutively requested between January 2015 and March 2023 at our Medical Genetics Centre. Each report belongs to a different patient or family, with a wide range of clinical indications.

**Results:** WES was performed in patients of all ages, including 40 in the prenatal period. Causal pathogenic or likely pathogenic variants were identified in 324 cases, resulting in an overall diagnostic yield of 37%. 4 patients had two diagnosis. In total, there were 223 autosomal dominant, 69 autosomal recessive, 32 X-linked and 1 mitochondrial conditions diagnosed. 3 patients were mosaic for an autosomal dominant disease. In 4 cases, new disease associations were established with additional collaborative work-up. 2 further patients have promising variants in candidate genes. Variants of uncertain significance were found in 186 families. In 73%, only the proband was tested. WES-trio, including the proband’s parents, was carried out in 23% of families, with a higher diagnostic yield (48% versus 33% in proband-only cases). Most patients (77%) had a neurodevelopmental phenotype. Interestingly, the rate of diagnosis was higher in individuals with neurological features, whether isolated or syndromic. Twenty one consenting subjects had variants in actionable genes belonging to the American College of Medical Genetics and Genomics list.

**Discussion:** Our diagnostic yield was slightly higher than in other case series with heterogeneous cohorts, with reported rates of 28 to 30%, which probably reflects differences in criteria for requesting WES across medical Centres. Trio analysis also improved the diagnostic yield and saved in subsequent segregation studies. WES was an invaluable tool in reaching etiological diagnosis, with an impact on counselling regarding prognosis, recurrence risk and, at times, therapeutic options.

#### OP4 - HICKAM’S DICTUM: KEEPING UP WITH THE DIAGNOSES

Susana Lemos Ferreira^1^, Joana A. Catanho^1^, Mafalda Melo^1^, Sofia Nunes^1^, Rui Gonçalves^1^, Marta Amorim^2^, Diana Antunes^1^, Márcia Rodrigues^3^, Teresa Kay^1^, Inês Carvalho^1^, Margarida Venâncio^1^

^1^ *Serviço de Genética Médica, Centro Hospitalar e Universitário de Lisboa Central, Lisboa, Portugal* ^2^ *Consulta de Genética Médica, Hospital dos Lusíadas, Lisboa, Portugal* ^3^ *Serviço de Genética Médica, Centro Hospitalar e Universitário de Lisboa Norte, Lisboa, Portugal.*

**Introduction:** Hickam’s dictum is the principle upon is statistically more likely that a patient with multiple symptoms has a combination of diseases rather than only one diagnosis that fully explains the complete phenotype. Dual genetic diagnoses (DGD) consist of distinct or blend clinical diagnoses of more than one locus that segregates independently. Increasing diagnostic yield with techniques such as whole exome sequencing (WES) is associated with rise of unbiased DGD. Data from literature estimates that 7% of cases have DGD.

**Aim and methods:** Retrospective review of pediatric patients with DGD referred to our Genetics Department (2001-2023), to identify and assess their clinical and genetic characteristics.

**Results:** 25 patients were identified with a confirmed or potentially DGD. In 20/25 DGD were confirmed, clinical consistent with the phenotype and genetic results were classified as likely pathogenic/pathogenic. In 5/25 cases, one variant was classified as uncertain significance but considered to be of potential clinical relevance. The main indication for referral was multisystemic disease 22/25. 3/25 patients were referred with a previous genetic diagnosis that did not fully explained the phenotype. Of the cases without previous diagnosis (22/25), 12 presented with atypical features, 2 had a blended phenotype and 8 had a second diagnosis after clinical validation of incidental/secondary findings reported. In most cases on 15/25 the first-tier test provided DGD. Primary diagnoses were obtained by WES in 13 cases and by array in 7, and secondary diagnoses by WES in 14 and array in 8.

**Conclusion:** DGD should be considered when a patient presents with atypical features, especially if associated with multisystemic phenotype. In most cases, clinical suspicion was the main drive to continue investigation. WES data improves our diagnostic capacity by identifying diagnoses that might be difficult to assert, even with deep phenotyping. This review provides important data suggesting that a diagnostic investigation is not necessarily complete after achieving one diagnosis and pursuing a DGD have implications in establishing patient’s prognosis, management and proper genetic counselling.

#### OP5 - AN INSIGHT ON THE ORIGINS OF HEREDITARY DIFFUSE GASTRIC CANCER THROUGH ORGANOID DRIVEN ORGAN ON-A-CHIP TECHNOLOGY

Daniel A. Ferreira^a,b^, Rita Barbosa-Matosa^b,c^, Luzia Garrido^d,e^, Ana Brito^a,b,c^, João Fonseca^a,b,d^, Silvana Lobo^a,b,c^, Ana Moutinho^a,b^, Alexandre Dias^a,b,c^, Irene Gullo^a,d,e^, Ana Mamede^a,b^, Meng Wei Yang^f^, Aldana D. Gojanovich^f^, Renata Oliveira^d,e^, João Freixo^d,g^, Pedro Louro^d,e^, Rita Quental^d,e^, Ana Grangeia^d,e^, Hugo Pinheiro^a,b,h^, Peter Ertli, Pedro Granja^a,j^, Susana Fernandese, Fátima Carneiro^a,d,e^, Sérgio Castedo^a,d,e^, Gustavo Mostoslavsky^f^, Carla Oliveira^a,b,d^

^a^ *i3S/Ipatimup – Instituto de Investigação e Inovação em Saúde/, Universidade do Porto;* ^b^ *Ipatimup – Institute of Molecular Pathology and Immunology, Universidade do Porto;* ^c^ *ICBAS - Instituto de Ciências Biomédicas Abel Salazar, Universidade do Porto;* ^d^ *Faculty of Medicine of the University of Porto;* ^e^ *CHUSJ, Centro Hospitalar e Universitário de São João;* ^f^ *Center for Regenerative Medicine, Boston University School of Medicine;* ^g^ *Center for Predictive and Preventive Genetics, Institute for Molecular and Cell Biology;* ^h^ *Department of Internal Medicine, Centro Hospitalar Tâmega e Sousa;* ^*i*^ *Faculty of Technical Chemistry, Vienna University of Technology (TUW);* ^*j*^ *INEB - Instituto de Engenharia Biomédica, Universidade do Porto*

**Introduction:** Hereditary Diffuse Gastric Cancer (HDGC), caused by CDH1 loss of function, can only be prevented with prophylactic removal of stomach and breasts. The growing number of prophylactic surgeries hamper the study of disease initiation. Herein, we present the development of a biomimetic HDGC model combining organ-on-a-chip technology with patient-derived induced pluripotent stem cells (iPSCs) to overcome the lack of patients’ target organs.

**Methodology:** We developed a microfabrication methodology based on xurography to engineer a functional stomach-on-a-chip, which was tested for biomimetic stretching capability and structural integrity. This was characterized with immuno-fluorescence, enzymatic, and trans-epithelial transport assays. Patients’ blood samples were collected for the establishment of a biobank of HDGC families with peripheral mononuclear blood cells (PBMCs) and its derived iPSCs. iPSCs were obtained using the Sendai virus integration-free method.

**Results:** We designed and fabricated a stomach-on-a-chip device emulating the 3 inner gastric layers and recapitulating gastric peristaltic-like motion, intraluminal-flow, cell polarization, barrier function, pepsin activity, and the gastric lumen. We also established and characterized iPSCs from 9 patient-derived PBMCs, which were validated for pluripotency and further differentiated into stomach organoids. The stomach-on-a-chip is ready to be populated with HDGC patient-derived organoids.

**Discussion:** We created a functional stomach-on-a-chip that can be produced in a few hours, and a unique infrastructure that will allow recreating organs that no longer exist from HDGC patients submitted to life-saving prophylactic surgery. This is a pioneer model to study early diagnosis and treatment in HDGC.

**Funding** Researcher/Horizon_Europe/i3S/2311/2022

LEGOHProject/PTDC/BTM-TEC/6706/2020

DETONATEProject/2022.02951.PTDC

### Cohorts

#### OP1 – CHARACTERIZING HERITABLE TP53-RELATED SYNDROME IN PORTUGAL: INSIGHTS FROM THE FIRST NATIONAL COHORT

Rita Quental^1^, Alexandre Dias^2^, Luzia Garrido^1,2^, Sónia Sousa^3^, Maria João Pina^3^, Pedro Louro^1^, Renata Oliveira^1^, João Parente Freixo^1,2,4^, Carla Pinto^5^, João Silva^6^, Catarina Macedo^7^, Juliette Dupont^7^, Patrícia Dias^7^, Raquel Gouveia Silva^7^, Mariana Soeiro e Sá^7^, Ana Berta Sousa^7^, Sofia Maia^8,9,10^, Jorge M. Saraiva^8,9,11^, Nataliya Tkachenko^12^, Ana Fortuna^12^, Gabriela Soares^13^, Márcia Martins^13^, Alexandra Gonçalves-Rocha^14^, Maria Lopes de Almeida^14^, Gabriela Sousa^15^, Joana Rosmaninho-Salgado^8,9,15^, Marta Amorim^16^, Catarina Machado^17^, Fabiana Ramos^8,9,18^, Inês Carvalho^19^, Diana Antunes^19^, Margarida Venâncio^19^, Sofia Fernandes^20^, Manuel R. Teixeira^5^, Sérgio Castedo^1,3^, Carla Oliveira^2^

^1^ *Serviço de Genética Médica, Centro Hospitalar de São João, Porto, Portugal;* ^2^ *Instituto de Investigação e Inovação em Saúde (i3S), Universidade do Porto, Porto, Portugal;* ^3^ *Instituto de Patologia e Imunologia Molecular da Universidade do Porto, Porto, Portugal;* ^4^ *CGPP-IBMC, Universidade do Porto, Porto, Portugal;* ^5^ *Serviço de Genética Laboratorial, Instituto Português de Oncologia do Porto, Porto, Portugal;* ^6^ *Serviço de Genética Médica, Instituto Português de Oncologia do Porto, Porto, Portugal;* ^7^ *Serviço de Genética Médica, Departamento de Pediatria, Hospital de Santa Maria, Centro Hospitalar Lisboa Norte, Lisboa, Portugal;* ^8^ *Serviço de Genética Médica, Hospital Pediátrico, Centro Hospitalar e Universitário de Coimbra, Coimbra, Portugal;* ^9^ *Centro Académico Clínico de Coimbra, Coimbra, Portugal;* ^10^ *Clínica Universitária de Genética, Faculdade de Medicina, Universidade de Coimbra, Coimbra, Portugal;* ^11^ *Clínica Universitária de Pediatria, Faculdade de Medicina, Universidade de Coimbra, Coimbra, Portugal;* ^12^ *Centro de Genética Médica Doutor Jacinto Magalhães, Centro Hospitalar Universitário de Santo António, Porto, Portugal;* ^13^ *Serviço de Genética Médica, Centro Hospitalar de Trás-os-Montes e Alto Douro, Vila Real, Portugal;* ^14^ *Unidade de Genética Médica, Hospital de Braga, Braga, Portugal;* ^15^ *Departamento de Oncologia Médica, Instituto Português de Oncologia de Coimbra Francisco Gentil, Coimbra, Portugal;* ^16^ *Unidade de Genética Médica, Hospital Dr. Nélio Mendonça, SESARAM;* ^17^ *Hospital Prof. Doutor Fernando Fonseca, Amadora, Portugal;* ^18^ *Hospital do Divino Espírito Santo de Ponta Delgada, Açores, Portugal;* ^19^ *Serviço de Genética Médica, Hospital Dona Estefânia, Centro Hospitalar Lisboa Central, Lisboa, Portugal;* ^20^ *Clínica de Risco Familiar, Instituto Português de Oncologia de Lisboa Francisco Gentil, Lisboa, Portugal.*

**Introduction:** Heritable TP53-related cancer syndrome is a rare inherited genetic condition that significantly increases an individual’s susceptibility to various types of cancer. This syndrome results from heterozygous pathogenic variants in the TP53 gene, which regulates the transcription of target genes involved in cell cycle control, DNA repair, and apoptosis.

**Methodology:** We performed a retrospective analysis of clinical and molecular data from families with TP53 germline variants collected from 12 Portuguese hospitals.

**Results:** Our dataset includes 91 families with pathogenic (P) or likely pathogenic (LP) TP53 germline variants. Two of these families had an additional deleterious variant in a different gene (ATM/CHEK2) and were excluded from the analysis. The remaining 89 families include 114 affected carriers (81 females, 32 males, 1 not reported) and 156 affected relatives with undetermined molecular status (66 females, 47 males, 43 not reported). Thirteen families fulfilled the classical LFS criteria, 42 met the revised Chompret criteria, and 34 did not fulfil any established criteria. Mosaicism was suspected or confirmed in 6 additional patients. Of the 270 affected individuals, 64 (24%) developed multiple tumours, predominantly females (68%). Thirty-eight patients received a cancer diagnosis in childhood, with sarcomas (53%), central nervous system (CNS) tumours (21%), and adrenocortical carcinoma (11%), being the most common malignancies. In adult patients, breast cancer (38%), sarcomas (12%), gastric cancer (8%), and CNS tumours (7%) were the most common. Regarding TP53 mutational spectrum, 47 different germline P/LP variants were identified: 30 were missense, 7 frameshift, 5 nonsense, and 5 splicing variants. In particular, the p.(Arg337His) was the most frequently observed variant, followed by p.(Arg282Gln), p.(Arg196*), p.(Arg158His), p.(Arg248Gln), and p.(Arg267Trp), which together accounted for 40% of the families. In 12 patients the TP53 variant was found to be de novo. Dominant-negative variants tend to cause tumours at an earlier age than loss-of-function variants (mean [SD] 37.9 years [17.9] vs. 39.8 years [17.4], p=0.176), although this difference did not reach statistical significance.

**Conclusions:** This study has increased our knowledge of hereditary TP53-related syndrome in the Portuguese population. It serves as a starting point for future analyses to better understand genotype-phenotype relationships, ultimately improving surveillance for individuals with disease-causing variants.

#### OP2 - CLINICAL AND MOLECULAR CHARACTERISATION OF BAF COMPLEX-RELATED SYNDROMES IN THE PORTUGUESE POPULATION

Daniela Oliveira^1,2,3^, Sara Pinho^4^, Joana Silva^5^, Rita Quental^6^, Inês V. Carvalho^7^, Inês Nunes Vicente^8^, Carla Marques^8^, Cristina Pereira^9^, João Paulo Oliveira^6^, Margarida Venâncio^5^, Ana Berta Sousa^4^, Jorge M. Saraiva^1,3,10^, Sérgio B. Sousa^1,2,3^

^1^*Medical Genetics Unit, Hospital Pediátrico, Centro Hospitalar e Universitário de Coimbra, Coimbra, Portugal;* ^2^ *University Clinic of Genetics, Faculty of Medicine, University of Coimbra, Portugal;* ^3^ *Clinical Academic Center of Coimbra, Portugal;* ^4^ *Medical Genetics Unit, North Lisbon University Hospital Centre, Portugal;* ^5^ *Medical Genetics Unit, Central Lisbon University Hospital Centre, Portugal;* ^6^ *Medical Genetics Unit, São João University Hospital Centre, Portugal;* ^7^ *Neurology Unit, Coimbra Hospital and University Centre, Portugal;* ^8^ *Child Development Centre, Paediatric Hospital, Coimbra Hospital and University Centre, Portugal;* ^9^ *Paediatric Neurology Unit, Paediatric Hospital, Coimbra Hospital and University Centre, Portugal;* ^10^ *University Clinic of Paediatrics, Faculty of Medicine, University of Coimbra, Portugal*

**Introduction:** The BAF chromatin remodelling complex plays a crucial role in regulation of gene expression, cell differentiation, and neural development. The BAF complex-related syndromes are a growing group of autosomal dominant conditions resulting from mutations in over 29 genes that disrupt its function. In this group, Coffin-Siris and Nicolaides-Baraitser syndromes were the first to be recognised but several other intellectual disability (ID) phenotypes are being delineated.

**Methodology:** Clinical and molecular characterisation of cases with BAF complex-related syndromes from four national hospital centres based on retrospective analysis of medical records.

**Results:** We describe 38 patients, from 37 different families, with molecularly confirmed BAF complex-related syndromes due to variants in ARID1B (n:19), ADNP (n:3), ARID2 (n:3), SMARCA2 (n:3), SOX11 (n:3), SMARCB1 (n:2), SMARCC2 (n:2), ARID1A (n:1), BCL11A (n:1) and SMARCA4 (n:1). In two cases, the variant was inherited. We report 17 females and 21 males aged from 4 to 34 years-old (age at diagnosis: 1–33). All assessed patients presented developmental delay/ID: 28% mild, 44% moderate, 24% severe and 4% profound, 27% of them with absent speech. Most patients had hypotonia (72%) and central nervous system abnormalities (70%), particularly anomalies of corpus callosum (40%), and seizures were present in 26%. Other frequent medical issues were visual impairment (69%), cryptorquidism (65%), feeding problems (53%) and frequent infections (53%). Most common dysmorphic features included broad nasal tip (57%), large mouth (52%), prominent eyelashes (52%), thick lower vermillion (50%), hypertrichosis (46%), thick eyebrows (46%), coarse face (45%), broad nasal base (43%), and hypoplastic 5th finger/toenail (38%).

**Discussion:** BAF complex-related syndromes are a frequent group of neurodevelopmental disorders, being this multicentre study the first on a large cohort of Portuguese patients. Classical associated features, such as ectodermal anomalies, coarse face and 5th digit hypoplasia, were also commonly found in our cohort. However, we report a higher incidence of seizures and absent speech. ARID1B was confirmed as the most frequent gene involved. Thorough description of population cohorts contributes to increase an in-depth knowledge of their natural history, genotype-phenotype correlations, and to accurate counselling and personalised management of these patients on a national context.

### Clinical Cases

#### OP1 - A NOVEL FRAMESHIFT VARIANT IN NADSYN1 GENE ASSOCIATED WITH VERTEBRAL, CARDIAC, RENAL, AND LIMB DEFECTS SYNDROME 3

Marisa Rodrigues^1,2^, Leonor Dias^2,3^, Ana Correia-Costa^1,2^, Ana Grangeia^2,4^, Miguel Leão^4^

^1^ *Serviço de Cardiologia Pediátrica do Centro Hospitalar de São João, Porto, Portugal;* ^2^ *Faculdade de Medicina da Universidade do Porto, Portugal;* ^3^ *Serviço de Neurologia do Centro Hospitalar de São João, Porto;* ^4^ *Serviço de Genética Médica do Centro Hospitalar de São João, Porto.*

**Introduction:** Nicotinamide adenosine dinucleotide (NAD) synthetase 1, encoded by NADSY1 gene, is responsible for the last step in NAD biosynthesis, whose alterations impair cell metabolism and embryogenesis. We report a case of a 17yo girl with spondylocostal dysostosis (SCDO), aortic coarctation (CoA) and renal failure.

**Clinical case:** Third child born to healthy and non-consanguineous parents with irrelevant family history. Gestational diabetes was diagnosed, and fetal karyotype performed due to advanced maternal age was normal. The newborn was admitted to NICU on D2 of life due to suspected congenital heart disease. Postductal CoA was confirmed and surgically corrected at D5. She developed acute renal failure on the same day, progressing to end-stage renal disease. Thorax X-ray showed cervical and dorsal hemivertebrae with rib deformity of the left hemithorax. After discharge, she has a multidisciplinary follow-up. At age 5, she had her 1st genetic evaluation. Vertebral MR presented thoracic convexity scoliosis globally anomalous thoracic vertebrae with butterfly wing morphology. Additionally, right deafness and left transmission hypoacusis were diagnosed. Molecular studies of DLL3, MESP2, LFNG, HES7, JAG1 genes and aCGH were normal. At 15yo, she underwent surgery to correct severe scoliosis. Clinical exome was inconclusive. Recent reanalysis revealed 2 variants in apparently compound heterozygosity in NADSYN1 gene (NM_018161.5): c.1481_1484del (p.Tyr494Cysfs*5) and c.1717G>A (p.(Ala573Thr), allowing the diagnosis of NADSYN1-associated vertebral, cardiac, renal, and limb defects (VCRL) syndrome 3 with autosomal recessive inheritance. Progenitors’ segregation studies are in course.

**Discussion:** The association of bi-allelic pathogenic variants in NADSYN1 with multisystemic presentation (vertebral, cardiac, renal and limb abnormalities) was described by Szot et al (2020). Only 19 clinical cases of NADSYN1-associated VCRL syndrome 3 have been published, and none with hypoacusia (Aubert-Mucca et al, 2023). Also, the frameshift variant c.1481_1484del has never been reported. This work expands the clinical and genetic spectrum of NADSYN1-associated VCRL syndrome 3.

#### OP2 - HIGH-LEVEL OF GNA11 MOSAICISM IN A CASE OF SYNDROMIC CUTIS MARMORATA TELANGIECTATICA CONGENITA– A CASE REPORT COMPARED WITH THE LITERATURE

Mariana Neves^1^, Patrícia Dias^1^

^*1*^ *Serviço de Genética Médica, Departamento de Pediatria, Hospital de Santa Maria, Centro Hospitalar Universitário Lisboa Norte, Lisboa, Portugal*

**Introduction:** The GNAQ and GNA11 genes codify for proteins mediating signals between G protein coupled receptors and downstream intracellular effectors, including the ones in endothelial cell signalling linked to the RAS/MAPK pathway. Postzygotic variants in these genes have been implicated in different types of cutaneous capillary malformations both in isolated lesions and complex syndromic pathologies. We report a patient with high-level GNA11 mosaicism and a complex syndromic phenotype with cutis marmorata telangiectatica congenita (CMTC).

**Case report:** 17-year-old female, referred to genetics at 22 months due to CMTC, hypotonia and global developmental delay (GDD). She presented microcephaly, left body hemihypoplasia, large Mongolian spot, glabellar hemangioma, facial telangiectases and cutis marmorata predominantly of the limbs and the trunk. This skin phenotype improved with age. She evolved with moderate intellectual disability, attention deficit hyperactivity disorder and behavioural problems. At 7 years of age she was diagnosed with arterial hypertension and multicystic kidneys. Additionally she had hemiplegic migraine and Raynaud phenomenon complicated with painful perniosis lesions. Capillaroscopy revealed sclerodermic changes with dilated capillaries and megacapillaries. At 16 years she was hospitalized with an acute digestive haemorrhage, portal hypertension and gastroesophageal varicose veins. Portal angio-TC revealed intra-hepatic portal veins absent/difficult to visualize and profuse collateral vascularization. No germinal variants were identified in aCGH and WES testing, however WES testing in DNA from a mucosa swab uncovered a c.548G>A, p.(Arg183His) variant in GNA11 gene with an allele frequency (VAF) of 25%.

**Discussion and conclusions:** Postzygotic variants in codon 183 of GNA11 gene have been associated with a diverse range of congenital capillary malformations and variable systemic manifestations. A previously reported patient harbouring the same variant1 but with a smaller VAF of 6-9% presented mild GDD, mixed vascular naevus and arterial hypertension. We compare our case with 14 additional patients1-7 with different variants affecting this codon, all with lower levels of mosaicism. Body asymmetry (10/15), GDD (4/15) and arterial hypertension (3/15) were the most common additional features, and none was described with internal organ vascular abnormalities identical to our patient. The severity of our patient’s phenotype might be explained by the higher level of mosaicism.

**Referências:** 1 Beteta-Gorriti V. et al. Mixed vascular naevus syndrome: report of three children with somatic GNA11 mutation and new systemic associations. Clin Exp Dermatol. 2022 Jan;47(1):129-135. doi: 10.1111/ced.14848; 2 Thomas AC. et al. Mosaic Activating Mutations in GNA11 and GNAQ Are Associated with Phakomatosis Pigmentovascularis and Extensive Dermal Melanocytosis. J Invest Dermatol. 2016 Apr;136(4):770-778. doi: 10.1016/j.jid.2015.11.027; 3 Sliepka JM. Et al. GNA11 brain somatic pathogenic variant in an individual with phacomatosis pigmentovascularis. Neurol Genet. 2019 Oct 30;5(6):e366. doi: 10.1212/NXG.0000000000000366; 4 Couto JA. Et al. A somatic GNA11 mutation is associated with extremity capillary malformation and overgrowth. Angiogenesis. 2017 Aug;20(3):303-306. doi: 10.1007/s10456-016-9538-1; 5 Davies OMT. Et al. Early-onset hypertension associated with extensive cutaneous capillary malformations harboring postzygotic variants in GNAQ and GNA11. Pediatr Dermatol. 2022 Nov;39(6):914-919. doi: 10.1111/pde.15103; 6 Schuart C. et al. Cutis marmorata telangiectatica congenita being caused by postzygotic GNA11 mutations. Eur J Med Genet. 2022 May;65(5):104472. doi: 10.1016/j.ejmg.2022.104472; 7 Beteta-Gorriti V. et al. Mixed vascular naevus syndrome: report of three children with somatic GNA11 mutation and new systemic associations. Clin Exp Dermatol. 2022 Jan;47(1):129-135. doi: 10.1111/ced.14848.

#### OP3 - FIRST FAMILIAL CASE OF 16P13.3 MICRODELETION SYNDROME – TWO NEW CASES ILLUSTRATING THE PHENOTYPE OF THIS RECENTLY DESCRIBED SYNDROME

C. Macedo^1^, R. Rodrigues^1^, M. Soeiro e Sá^1^, J.P. Monteiro^2^, A. Sousa^1^, A.B. Sousa^1^

^1^*Serviço de Genética Médica, Departamento de Pediatria, Hospital de Santa Maria, Centro Hospitalar Universitário Lisboa Norte, Lisboa, Portugal;* ^2^ *Centro de Desenvolvimento da Criança Torrado da Silva, Serviço de Pediatria, Hospital Garcia de Orta, Lisboa, Portugal.*

**Introduction:** Deletions within 16p13.3, encompassing the genes TBC1D21 and ATP6V0C and distal to CREBBP, have been associated with a novel microdeletion syndrome. Initially described in 2019, this emerging clinical entity is characterized by microcephaly, developmental delay (DD)/intellectual disability (ID), and epilepsy. Epilepsy typically manifests within the first few years of life, with all affected individuals experiencing generalized seizures requiring polytherapy.

**Methodology:** Detailed phenotypic and molecular characterization of two related affected patients.

**Results:** We report a 2-year-old boy born to non-consanguineous parents, referred to our clinic due to DD (he started walking at 20 months of age and said his first words at nearly 2 years old) and behavioural problems (hyperactivity and social interaction deficits). On examination, he presented hirsutism, microcephaly, brachycephaly, arched eyebrows, mild synophrys, hypertelorism, bilateral epicanthus, upslanted palpebral fissures, depressed nasal bridge, and 5th finger clinodactyly. The EEG revealed mild diffuse slowing of brain activity, with no recorded seizures. Abdominal and kidney ultrasounds were normal. His mother had DD/ID and refractory epilepsy since age 14, manifested as tonic-clonic seizures with right frontal focal onset, and nocturnal predomination. She presented with microcephaly and a similar facial gestalt. ArrayCGH analysis revealed an heterozygous 16p13.3 deletion in both, spanning 322.62 kb. Subsequent analysis of the 16p13.3 region in the unaffected maternal grandmother yielded normal results. The maternal grandfather was not available for testing.

**Discussion:** Our family contributes to the recognition of 16p13.3 microdeletion syndrome as a distinct clinical condition. Our cases display notably similar dysmorphism, but differ from those in the literature, suggesting familial traits rather than a recognizable facial gestalt. We report the latest onset of epilepsy to date, highlighting the variable expressivity of this feature, but nonetheless requiring polytherapy, as in previously reported patients. To our knowledge, this is the first familial case report of this syndrome.

#### OP4 - POSSIBILITY OF PRENATAL DIAGNOSIS IN THE OFFSPRING OF HEALTHY ROMANI COUPLES: TWO RECESSIVE PATHOLOGIES, THREE CASES, FOUR DIAGNOSES

Gonçalo Aragão^1^, Fabiana Ramos^2,3^, Pedro Almeida^2^, Miguel Branco^3^, Luís Abreu^3^, Carolina Ribeiro^1^, Filipa Melo^1^, Alexandra Lopes^1^, Rita Cerqueira^1^, Joaquim de Sá^1,2,3^, Marisa Teixeira^1^

^1^*CGC Genetics, Unilabs;* ^2^ *Serviço de Genética Médica, Centro Hospitalar e Universitário de Coimbra;* ^3^ *Centro de Diagnóstico Pré-natal, Centro Hospitalar e Universitário de Coimbra*

**Introduction:** In Portugal, healthy Romani couples descendants have an increased risk of autosomal recessive diseases, even if the couple’s consanguinity is no greater than that of the general population. The most efficient test for the etiological diagnosis of ultrasound abnormalities at present is whole exome sequencing. The wide scope of this test has allowed the identification of individuals with two pathologies in a significant proportion of diagnosed cases.

**Method:** Description of three clinical cases with no known family links: (1) Boy with severe developmental delay, structural heart disease and facial dysmorphisms; deceased older brother with identical phenotype; parental consanguinity (r = 1/8). (2) Ex-fetus submitted to TOP due to bilateral ventriculomegaly, hypoplasic nose, macrocephaly and increased abdominal perimeter; parents with no known consanguinity. (3) Prenatal diagnosis of ventriculomegaly, post-axial polydactyly in both feet and insufficient renal differentiation (cortical and medullary); in the neonate described hypotonia, unconjugated eye movements and facial dysmorphisms; parental consanguinity (r = 1/16). Whole exome sequencing performed at Illumina Novaseq 6000, data processed with in-house pipeline, analysis by NGS software.

**Results:** In all cases a pathogenic homozygous exon 23 deletion of TBCK gene was found. In the third case a likely pathogenic homozygous loss-of-function variant was also identified in the BBS2 gene [c.402del p.(Ala136Argfs*65)]. All parents were heterozygous. This established the diagnosis of hypotonia infantile with psychomotor retardation and characteristic facies [MIM 616900] and Bardet-Biedl syndrome 2 [615981].

**Discussion:** In literature, exome sequencing is already the first study to clarify the etiology of ultrasound or neurodevelopmental anomalies and the laboratory now has to be careful when deciding to conclude the study with a single diagnosis. We propose with these cases to start the discussion we will present on the application of that test in the preconceptional context in healthy people, to assess the future offspring risk for autosomal recessive or X-linked diseases, as well as the ACMG secondary results.

#### OP5 - UNEXPLAINED CARDIORESPIRATORY ARREST IN PEDIATRIC AGE: POSTMORTEM DIAGNOSIS OF ARRHYTHMOGENIC CARDIOMYOPATHY

Catarina F. Silva^1^, Mafalda Melo^2^, Gabriela Pereira^3^, Petra Loureiro^1^, Sérgio Laranjo^1^, Sílvia Aguiar^4^, Conceição Trigo^1^, Rafael Graça^5^, Fátima Pinto^1^, Margarida Venâncio^2^, Diana Antunes^2^

^1^ *Serviço de Cardiologia Pediátrica, Hospital de Santa Marta, Centro Hospitalar Universitário de Lisboa Central, Lisboa, Portugal, Centro de Referência de Cardiopatias Congénitas do CHULC, Lisboa, Portugal, Membro da European Reference Network (ERN) Guard-Heart, Centro Clínico Académico de Lisboa, Portugal;* ^2^ *Unidade de Genética Médica, Hospital Dona Estefânia, Centro Hospitalar Universitário de Lisboa Central, Lisboa, Portugal;* ^3^ *Unidade de Cuidados Intensivos Pediátricos, Hospital Dona Estefânia, Centro Hospitalar Universitário de Lisboa Central, Lisboa, Portugal;* ^4^ *Serviço de Cardiologia, Hospital Santa Marta, Centro Hospitalar Universitário de Lisboa Central, Lisboa, Portugal;* ^5^ *GenoMed – Diagnósticos de Medicina Molecular.*

**Introduction:** Unexplained Cardiorespiratory arrest (uCRA)/Sudden Death(SD) raises concerns regarding hereditary cardiac diseases. The European Society of Human Genetics (ESHG) has published recommendations highlighting the importance of conventional and molecular autopsies. Current guidelines recommend cardiac evaluation of first-degree family members of individuals who experienced uCRA/SD.

**Clinical case:** We present the case of a 9-year-old boy with no previous history of cardiovascular disease who was brought to the emergency room following an uCRA during the night. His brother reported hearing a gasping sound followed by profuse vomiting and loss of consciousness. Upon the arrival of the emergency response team, the patient exhibited a non-shockable rhythm however, following one administration of adrenaline, the patient transitioned into ventricular fibrillation and the team was able to restore spontaneous circulation after two shocks. When admitted to the intensive care unit, the child displayed isochoric and photoreactive pupils however, his neurological status deteriorated. An MRI revealed severe, non-reversible, hypoxic-ischaemic brain lesions. The cardiac evaluation did not reveal any structural heart anomalies. Molecular analysis of a comprehensive panel targeting genes associated with myocardiopathies and rhythm disorders was initiated. Brain death was declared on the ninth day after admission.

Genetic testing identified heterozygotic variants in KCNH2 and FLNC genes. Although both mutations were classified as variants of unknown significance, the KCNH2 variant has been previously reported in Long QT syndrome, while the FLNC variant was previously reported in arrhythmogenic myocardiopathy. The autopsy found the cause of death to be an arrhythmogenic myocardiopathy of the right ventricle complicated by anoxic encephalopathy after cardiac arrest.

Additional testing was extended to first-degree family members to clarify these results. Both variants were detected in the patients’ asymptomatic 18-year-old brother. Additionally, the mother was confirmed to be a carrier of the KCNH2 variant. Both individuals exhibited normal QT intervals on their EKGs and underwent cardiac MRI with no major structural abnormalities.

**Discussion:** This case highlights the complexities of uCRA/SD. Despite the unfortunate outcome in the index case, it prompted the establishment of a multidisciplinary team to assess and monitor at-risk family members. Both family members, while currently asymptomatic, were scheduled for regular cardiologic imaging and follow-up.

## Poster presentation

### Basic Research

#### P1 - SUBLINEAGES OF THE Y-CHROMOSOME HAPLOGROUP E-M78 IN THE PORTUGUESE POPULATION

Diana Alves, Selsabil Bouabdallah, Licínio Manco

Research Centre for Anthropology and Health (CIAS), Department of Life Sciences, University of Coimbra, Coimbra, Portugal

**Introduction:** The Y-chromosome lineage E1b1b1a1-M78, a main subclade of the E1b1b1-M35 haplogroup, has a broad geographical range, encompassing North and East Africa, Europe, and Western Asia1. In Portugal, it represents frequencies of about 4%; however, their main subclades were poorly addressed in Portuguese samples until now. In present study, we examined a set of unrelated men from mainland Portugal for the most known E-M78 sublineages and respective internal microsatellite diversity.

**Methodology:** A total of 17 individuals, previously typed for the M78 marker (12 from central and five from northern regions of mainland Portugal), were analyzed for the three main E-M78 sublineages V12, V13, and V22. Genotyping was made by PCR-RFLP or Sanger sequencing using primers previously described. The internal variation of E-M78 samples was evaluated by the analysis of seven Y-STRs (DYS19, DYS389I, DYS389II, DYS390, DYS391, DYS392, DYS393) using primer sequences obtained at https://strbase-archive.nist.gov/ystr_fact.htm. Multiplex PCR reactions were carried out using the QIAGEN Multiplex PCR kit (Qiagen) with the forward primers labelled on the 5’ end and separation of DNA fragments in an ABI PRISM 3130 Genetic Analyzer (Applied Biosystems).

**Results:** Most individuals belong to the V13 subclade (n = 9) (52.9%). Two individuals belong to the V12 subclade (11.7%) and one to the V22 subclade (5.9%). Five individuals (29.4%) were assigned to the paragroup M78*. The seven analyzed Y-STRs defined a set of 13 different haplotypes in the Portuguese sample set. We found no haplotypes shared by different subclades.

**Discussion:** We highlighted the most common sublineages in a set of M78-derived Y-chromosomes of Portuguese individuals. Most E-M78 chromosomes (about 53%) belong to the E-V13 subclade, similar to results found in other European populations1. The E-V13 subclade was suggested to have a post-Neolithic expansion into Europe from the Near East, where it originated, via the Balkans. The E-M78 subhaplogroups E-V12 and E-V22, also found in the Portuguese population, could was involved in trans-Mediterranean migrations directly from northern Africa, probably during the Islamic period in the territory. The Y-STR analysis encompasses a collection of different haplotypes in the Portuguese samples, suggesting the probability of different evolutionary histories for the E-M78 chromosomes in the country.

**Reference**

1. Cruciani et al. Mol. Biol. Evol. 2007;24(6):1300–1311.

#### P2 - NON-CANONICAL SYNTHESIS OF UPF1 PROTEIN CONTRIBUTES TO ITS ONCOGENIC ROLE IN COLORECTAL CANCER

Rafaela Lacerda^1,2^, Juliane Menezes^1,2^, Adriana Elias^1,2^, Luísa Romão^1,2^

^1^*Departmento de Genética Humana, Instituto Nacional de Saúde Doutor Ricardo Jorge, Av. Padre Cruz, s/n, 1649-016 Lisboa, Portugal;* ^2^ *BioISI — Biosystems and Integrative Sciences Institute, Universidade de Lisboa, 1749-016 Lisboa, Portugal*

Colorectal cancer (CRC) is the third leading cause worldwide and projections point towards an increase over the next two decades. Gene expression dysregulation of several genes involved in CRC contribute to disease development.

The up-frameshift 1 (UPF1) protein plays important roles in several cellular mechanisms and acts as a tumour suppressor in most cancers. However, in CRC, this protein has been described as working as an oncogenic protein. In order to understand the molecular mechanisms underlying the oncogenic role of UPF1 in CRC, we have analysed mRNA and protein levels in different types of cancer. In silico analyses have shown that UPF1 is overexpressed in CRC and lung cancer compared to the other analysed cancers. Also, UPF1 expression is significantly greater in CRC than in normal tissues. Experimentally, we observed that UPF1 expression is maintained under stress conditions that compromise global protein synthesis. In this regard, we tested whether UPF1 translation initiation can be mediated through an alternative cap-independent mechanism. We showed that the 5’ untranslated region (UTR) of UPF1 transcript allows cap-independent translation initiation and mapped the minimal sequence required for this mechanism to work. This region also mediates translation initiation in transcripts lacking a cap structure and under stress conditions like endoplasmic reticulum stress, hypoxia and mTOR pathway inhibition. Then, we designed antisense RNA oligonucleotides (ASOs) that target the minimal region and observed a reduced expression of UPF1 in CRC cells treated with those ASOs compared to cells treated with control ASOs.

All in all, these results show that alternative translation initiation mediated through UPF1 5’UTR allows UPF1 expression levels to be maintained under conditions observed in the tumour microenvironment, which globally repress protein synthesis. Thus, ASOs targeting the minimal region responsible for allowing UPF1 expression can be the beginning of a new RNA-based therapy to prevent CRC development.

#### P3 - DILATED CARDIOMYOPATHY WITH A DOUBLE GENETIC DIAGNOSIS IN ARRHYTHMOGENIC-RELATED GENES

André Ferreira^1^, Miguel Antunes^1^, Mafalda Melo^2^, Manuel Nogueira da Silva^3^, Silvia Aguiar Rosa^1^, Pedro Silva Cunha^1^, Guilherme Portugal^1^, Pedro Brás^1^, José Viegas^1^, Isabel Cardoso^1^, Yuri Chiodo^4^, Mário Oliveira^1^, Margarida Venâncio^2^, Diana Antunes^1,2,4^

^1^ *Centro Hospitalar Universitário de Lisboa Central, EPE/ Hospital de Santa Marta;* ^2^ *Centro Hospitalar Universitário de Lisboa Central, EPE/ Hospital de D. Estefânia;* ^3^ *Hospital Cuf Descobertas;* ^4^ *GenoMed - Diagnósticos de Medicina Molecular SA.*

We report a complex case of a family with a clinical diagnosis of dilated cardiomyopathy that after genetic testing was found to have two pathogenic variants in arrhythmogenic-related genes after genetic testing.

The index patient was referred to the CardioGenetic’s Clinic following a diagnosis of dilated cardiomyopathy detected in a screening echocardiogram for a family history of heart disease. This patient had an uncle with heart transplantation for dilated cardiomyopathy and two other family members with sudden death, an uncle died at 37 yrs old and his son died at 13 yrs old. It was decided to perform genetic testing on the patient with the most severe phenotype, the family member who was submitted to a heart transplant. Previous to the heart transplant, the patient had severe biventricular systolic dysfunction.

A large panel, including genes for cardiomyopathy and arrhythmia, was performed through next-generation sequencing, which revealed a complex genotype: a pathogenic truncating variant in the FLNC gene c.6976C>T, p. (Arg2326)* [NM_001458.4] (ACMG/ACGS 2019:PVS1, PS4_sup, PM2, PP1_str) and also a missense variant at the LMNA gene c.1071C>A, p. (Asp357Glu) [NM _170707.4], not previously reported neither in the literature or populational data; in spite in silico analysis predicted not to be pathogenic, it was in the same residue where other pathogenic variants were reported (ACMG/ACGS 2019:PM1, PM2, PP1_sup, PP2, PM5, BP4). This patient was also found to have two variants of unknown clinical significance, in the DSP gene [NM _004415.3] c.1696G>A, p.(Ala566Thr) and ANK2 gene [NM_001148.4] c.9215A>G p.(Asp3072Gly), possibly benign. Family segregation studies validated the pathogenicity of the LMNA variant, found to be present in two other family members with positive phenotype. Cascade screening revealed additional family member carriers of the truncating FLNC variant in heterozygosity, and carriers of the LMNA variant in heterozygosity. None of other family members were found, yet, to inherited the two pathogenic variants. Two other relatives were already proposed for an implantable cardioverter defibrillator. Sudden cardiac death risk stratification is being performed in all family members available.

This report illustrates the importance of a multidisciplinary team, the absolute benefit of genetic testing in clarifying clinical diagnosis and the importance of awareness for the possibility of unexpected results with utter importance for following up of patients, including primary prevention of sudden death.

#### P4 - COMMON MECHANISTIC PATHWAYS IN RARE CONGENITAL SYNDROMES WITH PRIMARY MICROCEPHALY

Xavier Jorge^1^, Inês Milagre^2^, Anita Ferreira^1^, Sofia Calado^1^, Raquel Oliveira^2,4^, Sara Carvalhal^1,3^

^1^ *Algarve Biomedical Center - Research Institute, Univeristy of Algarve, Portugal;* ^2^ *Católica Biomedical Research Centre, Católica Medical School, Universidade Católica Portuguesa, Portugal;* ^3^ *Faculty of Medicine and Biomedical Sciences, Univeristy of Algarve, Portugal;* ^4^ *Instituto Gulbenkian de Ciência, Portugal.*

Primary microcephaly is an often-seen phenotype in several rare congenital syndromes. It is characterised by a smaller brain size at birth compared to the norm. The causes of this malformation are not fully understood, but genetic testing suggests a connection with defective genes involved in mitotic regulation and proteins related to DNA repair and replication pathways.

Cohesinopathies represent a group of rare syndromes, where several subtypes exhibit spontaneous railroad chromosomes and primary microcephaly. This includes Roberts Syndrome, Warsaw Breakage Syndrome and a recently characterised syndrome caused by mutations in the BUB1 gene. Currently, we are examining fibroblast cells from patients with these syndromes to identify common mechanistic pathways.

In this context, we have identified a new promising candidate: Topoisomerase II alpha, a protein responsible for resolving of the DNA catenation both in the DNA replication and mitosis. Defective localisation of Topoisomerase II alpha may contribute to the observed mitotic defects in these cells. We are currently exploring the impact of these defects on brain development using reprogramming techniques to assess proper neuronal differentiation.

#### P5 - TRANSLATIONAL CONTROL OF Δ160P53 KEEPS THE DARK Side of TP53 in Check

Ana Catarina Ramalho^1,2^, Rafaela Lacerda^1^, Luísa Romão^1^, Marco M. Candeias^1,2^

^1^*Department of Human Genetics, National Institute of Health Doutor Ricardo Jorge, Lisbon, Portugal.* ^2^ *MaRCU – Molecular and RNA Cancer Unit, Graduate School of Medicine, Kyoto University, Kyoto, Japan.*

The TP53 tumour suppressor gene was discovered over 40 years ago, but to this day some aspects of its regulation and function remain a mystery. It encodes the full-length p53 protein (FLp53), a transcription factor with a key role in stress response in multicellular organisms, that can either direct cells towards apoptosis or recovery of homeostasis. With such a decisive role, its expression and activity are tightly regulated. A vast set of RNA-binding proteins (RBPs) have been described to affect the translation of FLp53 or the stability of p53 mRNA in response to different perturbations. But in addition to FLp53, there is a group of shorter protein isoforms lacking the N-terminal region, which have well-described functions, and are translated from the same mRNA. The shorter and less studied isoform is Δ160p53, which promotes cell survival, proliferation, and invasion. Despite its usual low levels, it is commonly overexpressed in tumours. However, the detailed mechanisms and factors involved in the regulation of Δ160p53 are still unknown.

In this work, a mass spectrometry was performed to identify the proteins in an RNA-protein co-immunoprecipitation of the p53 mRNA using the MS2 system in the p53-null cell line H1299. The validation of the hits was undertaken by western blot with specific antibodies after immunoprecipitation. The effect of the binding proteins on the translation of Δ160p53 was assessed by overexpression or knockdown, and the expression levels were verified by western blot or luminescence assays.

The mass spectrometry allowed the identification of potential new binding partners of the p53 mRNA. Resorting to the literature and to computational tools available online to predict protein-RNA interactions, a few hits were selected for follow-up and their interactions confirmed. Simultaneously, the modulation of Δ160p53 expression by some of these proteins was verified.

Considering the importance of TP53 in deciding the fate of the cell, the observation of abnormal levels of the oncoprotein Δ160p53 in cancer is intriguing. Understanding the control of its translation could uncover strategies to block it and pave way for new cancer therapies.

#### P6 - THE Y-CHROMOSOMAL HAPLOTYPE DIVERSITY OF R1B-M269 SUBHAPLOGROUPS IN INDIVIDUALS FROM THE CENTRAL REGION OF PORTUGAL

Fábio Nunes^1^, Licínio Manco^1^

^1^ *Research Centre for Anthropology and Health (CIAS), Department of Life Sciences, University of Coimbra, Coimbra, Portugal*

**Introduction:** The most frequent Y-chromosomal haplogroup in the Iberian Peninsula is R1b-M269, representing frequencies of about 60% in Portugal. The R1b-M269 splits into geographically localized subhaplogroups, showing the S116-DF27 branch as the most common in the Iberian Peninsula (40-48%) [1]. We have previously shown that the subhaplogroup DF27 was the most common (70%) among R1b-M269 individuals from Central Portugal [2]. This study aimed to analyze the distribution of the Y-chromosomal haplotypes within the main branches of the R1b-M269 haplogroup in individuals from the central region of Portugal.

**Methodology:** The study sample comprised 54 individuals carrying the derived allele at the M269 SNP from districts of Aveiro, Coimbra, Guarda and Viseu. The internal variation of R1b-M269 samples was assessed by analyzing seven Y-STRs (DYS19, DYS389I, DYS389II, DYS390, DYS391, DYS392, DYS393). Multiplex PCR reactions were performed using the QIAGEN Multiplex PCR kit. The forward primers were labeled with Cy5 on the 5’ end. The PCR fragments were analysed on an automate ALFexpressTM II sequencer (Amersham Biosciences). The frequencies of the Y-STR haplotypes were determined by direct counting. Population pairwise genetic distances (RSTs) and p-values were calculated using the software Arlequin v3.5.

**Results and Discussion:** The gene diversity of the seven markers ranged from 0.143 (DYS393) to 0.558 (DYS389II) for the sample set. The overall haplotype diversity was 0.343, similar to other Iberian populations (mean 0.368 for five Iberian populations [3]). The total number of observed haplotypes was 31, the most frequent (0.148) was compatible with the “Atlantic Modal Haplotype” (AMH) (DYS19-14/ DYS390-24/ DYS391-11/ DYS392-13/ DYS393-13). The network constructed with the 31 different haplotypes produced a star-like structure with a center occupied by the most common AMH-compatible haplotype (14-13-29-24-11-13-13), shared by eight samples and four different R1b-M269 suclades. The network showed only one missing link, which could indicate evolutionary histories of the R1b-M269 paternal lineages inside the region. The genetic distances between six Iberian populations showed no significant differences, except between Portugal and the Native Basques (RST=0.007; P=0.054).

**References**

1. Valverde et al (2016) Eur J Hum Genet 24:437-441.

2. Nunes & Manco (2022) Medicine (Proceeding Abstracts) 102:13

3. Villaescusa et al (2017) Forensic Sci Int Genet 27:142–148

#### P7 - A 3D CELL CULTURE MODEL OF THE TUBERCULOSIS GRANULOMA THAT CAN BE APPLIED FOR HOST GENETIC STUDIES IN THE CONTEXT OF A MULTICELLULAR IMMUNOLOGIC RESPONSE TO INFECTION

Susana David^1,2^, Manoj Mandal^2^, Elsa Anes^2^, David Pires^2,3^

^1^ *Departamento de Genética Humana, Instituto Nacional de Saúde Doutor Ricardo Jorge (INSA), Lisbon, Portugal.* ^2^ *Host-Pathogen Interactions Unit, Research Institute for Medicines, iMed.ULisboa, Faculty of Pharmacy, Universidade de Lisboa, Lisbon, Portugal.* ^3^ *Universidade Católica Portuguesa, Católica Medical School, Center for Interdisciplinary Research in Health, Rio de Mouro, Portugal.*

**Introduction:** The granuloma is an inflammatory infiltrate of mononuclear cells. Some bacterial infections are characterized by the formation of granulomas as part of the immune response to contain the infection. Granuloma models have contributed valuable insights into the genetic basis of granuloma formation during infection. For example, IFNGR1 and IFNGR2 variants have been found to disrupt the immune response, resulting in impaired granuloma formation and increased susceptibility to diseases by Mycobacterium sp. More easily implemented comprehensive models would facilitate the study of the different immune mechanisms and help identify new disease-associated genes. Our objective is to generate an in vitro 3D cell culture model using human primary cells and microspheres to generate a stratified granuloma model for future use in genetic, immunological and drug discovery studies.

**Methods:** A commercial system was used to encapsulate human peripheral blood mononuclear cells (PBMC) infected with GFP-expressing M. tuberculosis and maintained in culture for several weeks. The cellular constituents of these granulomas and their organization were characterized by fluorescence microscopy and flow cytometry as well as the viability of the cells and the extent of bacterial replication in factor of time.

**Results:** The results demonstrate a ready recruitment of cells towards infected macrophages, leading to the formation of densely populated aggregates. These aggregates maintained cell viability for several weeks and displayed an enhanced control of bacterial replication compared to the more common monolayer infection models. Moreover, the capsules can be easily disrupted when required to isolate genetic material for further analysis.

**Conclusion:** The proposed 3D model resembles some structural and cellular characteristics of the tuberculosis granuloma and maintains its stability beyond more common 2D models of infection. These preliminary results demonstrate that this model can be used to further explore the determinants of granuloma formation and host response to infection.

**Acknowledgements:** This study was supported by FCT – Fundação para a Ciência e a Tecnologia, I.P, under grants EXPL/SAU-INF/0742/2021 to D.P., UIDB/04138/2020 to iMed.ULisboa, UIDB/04279/2020 to the Center for Interdisciplinary Research in Health and CEECINST/00070/2021 to Universidade Católica Portuguesa. M.M. is supported by the fellowship 2021.07978.BD.

#### P8 - PRIME EDITING TESTING TO CORRECT A FABRY DISEASE NONSENSE MUTATION

Ana J. Duarte^1,2,3,4^, Luciana Moreira^1,2,3^, José Bragança^5^, Olga Amaral^1,2,3^

^1^*Department of Human Genetics – R&D Porto, National Heath Institute Doutor Ricardo Jorge (INSA, IP), Porto, Portugal;* ^2^ *Center for the Study of Animal Science (CECA - ICETA), University of Porto (UP), Portugal;* ^3^ *Associate Laboratory for Animal and Veterinary Science (AL4AnimalS), Portugal;* ^4^ *Instituto de Ciências Biomédicas Abel Salazar (ICBAS), Universidade do Porto (UP), Portugal;* ^5^ *Stem Cells Biology Laboratory - Algarve Biomedical Centre Research Institute (ABC-RI), Faculty of Medicine and Biomedical Sciences, University of Algarve (UAlg), Portugal*

**Introduction:** In 2019, David Liu’s laboratory developed Prime Editing (PE), a new CRISPR-Cas9 derived method where Cas9 is substituted by a nCas9 (nickase) producing a nick on the edited locus which diminishes the off-target edits (1). The prime editor is composed by nCas9 fused to a reverse transcriptase (RT), and also a prime-editing guide RNA (pegRNA) who gives the complementary to the target sequence and has the template of the new sequence that will be synthetized by the RT. In this work we want to correct, through PE, the nonsense mutation p.W287X (c.860G>A), present in the alpha-galactosidase gene (GLA), found in a Fabry Disease patient.

**Methodology:** Two approaches are under testing:

1. Using the Prime Editing All-in-One plasmid (PEA1-GFP, Addgene #171993) (2), this plasmid enables an assembly of all the components used in PE: the pegRNA plus a second-nick gRNA. The PEA1-GFP construct was lipodelivered to patient-derived iPSCs produced in our lab (INSAi002-A).
2. We envisage the use of a synthetic engineered pegRNA (epegRNA) (3) and a single guide RNA (sgRNA) along with an in vitro transcribed PE mRNA (wich contains the nCas9 and the RT). The PE mRNA is derived from the plasmid pCMV-PE2 (Addgene #132775).

**Results:** Preliminary results with PEA1-GFP method, showed low efficiency of plasmid delivery and apparent lack of correction. Further changes are under study. As a second approach, the use of synthetic RNAs is more promising (although we have not yet begun the experiments).

**Discussion:** Currently we are optimizing conditions for PEA1-GFP in order to improve the results. However, it is described that our second approach with synthetic RNAs can provide better editing results than plasmid use (4). For that reason, we hope to attain better results using this later method and achieve the correction of the mutation in the patient derived cells. Gene editing, resulting from a worldwide joint effort provides a promising method for tailored therapeutics in genetic diseases.

**References DOI:**

1 – 10.1038/s41586-019-1711-4;

2 – 10.1093/nar/gkab792;

3 – 10.1038/s41587-021-01039-7;

4–10.1038/s41596-022-00724-4

**Acknowledgements**

This work is underway at INSA’s DGH group UID/SA with internal funding and with financial support from FCT/MCTES: UIDB/00211/2020, PTDC/BIM-MEC/4762/2014 and SFRH/BD/118009/2016.

We thank all colleagues; Paul Thomas’s lab gift PEA1-GFP (Addgene #171993) and the kind support of David Liu’s lab, which includes pCMV-PE2 (Addgene #132775).The authors declare no conflict of interests.

#### P9 - THE ROLE OF CYTOCHROME P450 (CYP)-ENZYME COMPLEX PROTEIN FACTORS AND OTHER OXIDOREDUCTASES IN THE MECHANISMS OF ACQUIRED BREAST CANCER DRUG RESISTANCE TO DOXORUBICIN

Daniel Crispim^1^, José Rueff^1^, Michel Kranendonk^1^, Francisco Esteves^1^

^1^ *Center for Toxicogenomics & Human Health (ToxOmics), NOVA Medical School/Faculty of Medical Sciences, Universidade NOVA de Lisboa, Lisboa, Portugal.*

**Introduction:** Drug resistance (DR) is a major challenge in cancer therapy, estimated to contribute in 90% to cancer-related fatalities. Doxorubicin (DOX), one of the most widely used chemotherapeutics in breast cancer (BC) treatment, is metabolized by cytochrome P450 (CYP) enzymes and oxidoreductases. This study addresses the underexplored topic of drug metabolism’s role in DR development, at subtherapeutic levels of DOX.

**Methodology:** BC MCF-7 cells were used to develop 3D spheroid models. Expression levels of 92 phase I drug metabolizing enzymes (DMEs) were assessed by RT-qPCR of MCF-7 DOX-sensitive (DOXS) or DOX-resistant (DOXR 25, 35, or 45 nM) cells. Their microsomal fractions were used to measure the activities of specific CYP isoforms.

**Results:** A signature of 24 statistically differentially expressed genes and enhanced CYP3A dependent metabolism (dibenzylfluorescein O-debenzylation) were identified in DOXR cells, compared with DOXS cells.

**Discussion:** Most of the differentially expressed genes in DOXR cells had previously been correlated with chemoresistance and/or tumour progression in BC patients. The overexpression of CYP4B1, CYP26B1, FDXR, FMO5, and PAH, as well as the augmented CYP3A dependent metabolism, had already been observed in a previous report from our laboratory, using monolayer cultures of DOXR MCF-7 cells (Barata et al, 2022, PMID: 36360213). These outcomes highlight the potential use of encountered expression and activity profiles as predictive markers for DOX resistance development. When comparing results obtained from using monolayer and spheroid culture approaches, several specific differences were noticeable, demonstrating the critical importance of spatial organization on enzyme expression and activity in DOX resistance. In summary, our data reveals an intricate link between the expression and activity of DMEs and the development of acquired resistance to DOX in MCF-7 cells. Additionally, our results underscore the dynamic nature of DR development, transiently dependent on multiple pathways such as drug-, arachidonic acid-, retinoic acid-, and vitamin D-metabolisms.

Partly funded by the Research Center grant ToxOmics (UIDB/00009/2020).

#### P10 - AEXTRACELLULAR VESICLES IN BREAST CANCER-HEPATIC COMMUNICATION: THEIR ROLE IN DOXORUBICIN RESISTANCE

Carolina Ramos^1^, José Rueff^1^, Michel Kranendonk^1^, Francisco Esteves^1^

^1^ *Center for Toxicogenomics & Human Health (ToxOmics), NOVA Medical School/Faculty of Medical Sciences, Universidade NOVA de Lisboa, Lisboa, Portugal.*

**Introduction:** Breast cancer (BC) is a leading cause of cancer related death among women. Although progress has been made in BC treatment, chemotherapy resistance occurs frequently, hallmarked by altered drug metabolism. Extracellular vesicles (EVs) are involved in many biological processes, through cell-to-cell communication. We studied the effect of BC-derived EVs on Phase I drug metabolizing enzymes expression in hepatic cells, the major site of drug metabolism, and their role in drug resistance (DR). We used doxorubicin (DOX), widely used in BC treatment, as a model drug.

**Methodology:** Spheroids of the hepatic model HepG2, and of the BC cell model MCF7 (either parental or resistant to DOX 45 nM), were established. EVs, isolated from the two types of MCF7 spheroids were incubated for 24 hrs with HepG2 spheroids. Expression of 92 genes of HepG2 cells, incubated with DOX alone, or DOX in combination with the two types of MCF7-derived EVs, was assessed by RT-qPCR.

**Results:** EV-titers from MCF7 resistant spheroids (EVR) were significantly higher, when compared with those of DOX-sensitive spheroids (EVS). Expression of several genes were exclusively upregulated in the presence of either type of EVs (e.g., CYP1B1, 2B6, 2E1 and 3A4). When comparing EVS- and EVR-exposures, some HepG2 genes were differentially expressed: e.g., PAM, CYP19A1 (EVs specific) and SQLE, CYP2J2, CYP1A1 (EVR specific). Interestingly, up-regulation of SQLE expression (involved in cholesterol metabolism) was exclusive for EVR.

**Discussion:** Higher production of EVR, when compared with those of EVS, seem to be indicative for their signalling role in chemoresistance. Overexpression of HepG2 genes, induced by both types of EVs, are involved in xenobiotic biotransformation, suggesting that BC cells have common capabilities of drug metabolism, independent of their DR status. Additionally, genes found to be differentially expressed when comparing both types of EVs, are related to xenobiotic (some specific for DOX), fatty acid or cholesterol metabolism, previously implicated in tumour progression and/or chemoresistance. In summary, our data pinpoint to modulation of DR mediated by EVs in BC-liver axis.

#### P11 - Poster canceled

#### P12 - EVOLUTIONARY HISTORY AND GENE EXPRESSION OF ATAXIN-3 PARALOGS

Daniela Felício^1,2,3^, Maria Inês Martins^2^, Andreia Pinto^2^, Inês P. D. Costa^1,2^, António Amorim^1,2,4^, Alexandra M. Lopes^1,2^, Susana Seixas^1,2^, Sandra Martins^1,2^

^1^ *Instituto de Investigação e Inovação em Saúde (i3S), Porto, Portugal;* ^2^ *Institute of Molecular Pathology and Immunology of the University of Porto (IPATIMUP), Porto, Portugal;* ^3^ *Instituto Ciências Biomédicas Abel Salazar (ICBAS), University of Porto, Porto, Portugal;* ^4^ *Dep. Biology, Faculty of Sciences, University of Porto, Porto, Portugal.*

**Introduction:** Ataxin-3 gene (ATXN3; 14q32.1) encodes a ubiquitously expressed deubiquitinating enzyme, with homologues among metazoans, plants and protozoans. In humans, ATXN3 may present a (CAG)n expansion, responsible for Machado-Joseph disease (MJD/SCA3). A highly conserved gene copy, ataxin-3 like (ATXN3L; Xp22.2) encodes a protein shown to cleave ubiquitin substrates in vitro more efficiently than ATXN31. Interestingly, a study on another ataxin gene, ATXN1, demonstrated that its copy, ATXN1L, alleviated the neuropathology in mice2. Thus, it is of upmost importance to understand whether ATXN3 copies can have a functional role in disease pathogenesis.

**Methodology:** To identify ATXN3 copies, we retrieved highly homologous sequences in 33 primates. We explored ATXN3 paralogs through the analysis of interspecific sequence diversity, evolution rates and selective constraints in the reconstructed phylogenies. For ATXN3L, which displays a conserved reading frame, we analysed protein domain conservation and mRNA expression by performing qPCR in 16 human normal tissues.

**Results:** Our results suggested three independent retrotransposition events: 1) in Haplorrhini (~63 MYA) for ATXN3L; 2) before the Platyrrhini-Catarrhini split (~43 MYA) for ATXN3L2 (LOC100132280; 8q23.2 in humans); and 3) in Cercopithecidae (25-30 MYA) for ATXN3L3 (LOC699321; chr11 in Rhesus monkey). ATXN3L seems to be under selective constraints throughout primate evolution as the parental ATXN3, whereas ATXN3L2 gained premature stop codons that likely turned it into an inactive copy. ATXN3L3 appears as a younger retrocopy with predicted alternative reading frames. Finally, we confirmed that ATXN3L is expressed in human placenta, testis and brain (cortex and substantia nigra).

**Discussion:** Phylogenetic analyses support the functional relevance of ATXN3L; however, further studies will be important to assess if ATXN3L expression in brain is conserved in non-human primates and detect the presence of endogenous protein ex vivo.

**References:** 1Weeks et al 2011 J Biol Chem 286:4555; 2Bowman et al 2007 Nat Genet 39:373.

**Acknowledgments:** FCT: 2022.04896.PTDC; UI/BD/154402/ 2023 (DF), and CEECIND/00684/2017 (SM).

#### P13 - MINDDS-WiNGS FEDERATED PLATFORM IMPLEMENTATION: A SAFER APPROCH TO DATA ACCESS

Pedro Carneiro^1,2,3,*^, Benjamin Huremagic^4,*^, Nishkala Sattanathan^5^, Joris Robert Vermeesch^4^, Geert Vandeweyer^5,6^, Paula Jorge^1,2,3^, Cristina Candeias^1,2,3^, Adrian J. Harwood^7^, Yves Moreau^8^, Amin Ardeshirdavani^8,9^, Haleh Chizari^8^, Natália Oliva-Teles^1,2,3,10^

^1^*Serviço de Genética Laboratorial, Clínica de Genética e Patologia, Centro Hospitalar Universitário de Santo António (CHUdSA), 4099-001 Porto, Portugal;* ^2^*UMIB — Unidade Multidisciplinar de Investigação Biomédica, ICBAS — Instituto de Ciências Biomédicas Abel Salazar, Universidade do Porto, 4050-345 Porto, Portugal;* ^3^*ITR — Laboratory for Integrative and Translational Research in Population Health, 4050-600 Porto, Portugal;* ^4^*Department of Human Genetics, KU Leuven, Leuven, 3000, Belgium;* ^5^*Department of Medical Genetics, University of Antwerp, Antwerp, 2000, Belgium;* ^6^*Department of Medical Genetics, University Hospital of Antwerp, Edegem, 2650, Belgium;* ^7^*Neuroscience and Mental Health Innovation Institute (NMHII), Cardiff University, Cardiff CF24 4HQ, UK;* ^8^*ESAT-STADIUS, KU Leuven, Leuven, 3000, Belgium;* ^9^*Agilent Technologies, Leuven, 3000, Belgium;* ^10^*MEDCIDS— Departamento Medicina da Comunidade, Informação e Decisão em Saúde, Faculty of Medicine, University of Porto, 4200-450 Porto, Portugal.* ^*^Shared first authors

**Introduction:** Research using biomedical data generated in hospitals/biomedical health institutions has contributed to considerably increase in knowledge concerning neurodevelopmental disorders (NDDs) and their association with rare genetic variants. However, more could be achieved by pooling data from multisite studies together to create meta-cohorts. A major barrier is the sensitive/private nature of clinical/genomic data, due to strict legislation access, e.g. GDPR, making it hard to share information. A way forward is to implement systems that make specific features derived from personal, clinical, genomic and phenotypic data visible without transferring complete data sets, thus avoiding compromising individual’s data security/privacy. MINDDS-WiNGS is a federated online data platform that overcomes these constraints - instead of physically moving data for analysis among institutions, analysis is carried out in situ through a federated network with world-wide accessibility (https://doi.org/ 10.1101/2022.06.23.497325).

**Methodology:** MINDDS-WiNGS federated platform (FP) has two main components: MINDDS-connect, a means to make selected data elements findable via online search site for assembling virtual meta-cohorts; from which controlled access can be granted to WiNGS for genomic analysis/research of genetic variants. AIM: Installation in Centro Hospitalar Universitário de Santo António (CHUdSA) is ongoing, joining WiNGS network.

**Results/Discussion:** Variants have been accessed via WiNGS as Standard Variant Calling Files (VCFs) and stored in Cardiff University. Access to the platform is monitored internally, by an administrator and one or more principal investigators (PI). Registered users are assigned to a PI with access to the information associated with it. In clinical institutions data can be stored either on local servers or be cloud-based, with access to data limited to a user’s PI; but requests can be made to access another PI-restricted information. The compilation of patient’s clinical, genomic and phenotypic information is achieved by filling in meta-data fields. The MINDDS-WiNGS FP allows data visualization and analysis without the need to have a centralised database, promoting multidisciplinary research across multiple and international borders.

**Acknowledgments:** IG16210 MINDDS-connect, https://www.cost.eu/actions/IG16210/; UMIB/ICBAS/UP, supported by National Funds through FCT, frameworks UIDP/00215/2020 and UIDB/00215/2020. The authors declare no conflict of interest.

#### P14 - ACTIVITY INDUCED GENES EXPRESSION IS IMPAIRED IN POLYGLUTAMINE SPINOCEREBELLAR ATAXIAS

Inês T. Afonso^1,2,3,4^, David V. C. Brito^2^, Hilmar Bading^4^, Clévio Nóbrega^1,2^

^1^*Faculdade de Medicina e Ciências Biomédicas, Universidade do Algarve, Faro, Portugal;* ^2^ *Algarve Biomedical Center – Research Institute, Faro, Portugal*, ^3^ *PhD Program in Biomedical Sciences, Faculdade de Medicina e Ciências Biomédicas, Universidade do Algarve, Faro, Portugal;* ^4^ *Interdisciplinar Center for Neuroscience, Heidelberg, Germany.*

**Introduction: Introduction:** Polyglutamine Spinocerebellar ataxias (SCAs) are a group of 6 incurable genetic disorders, caused by an expansion of the trinucleotide cytosine-adenine-guanine in their causative genes, which produces a protein with an expanded glutamine region. This project focuses on the study of Spinocerebellar ataxia type 2 (SCA2) and type 3 (SCA3) (1), which are rare dominantly inherited disorders that primarily impair the cerebellum therefore leading to motor ataxia.

Activity-induced inhibitor of death (AID), are a group of pro-survival 9 genes which were found to be neuroprotector in several neurological disorders, including stroke, glaucoma, AD, HD, and ALS (2).

In this project, we aim to investigate about the relevance of the expression of AID genes for cerebellum function and whether their expression levels are impaired in SCA2 and SCA3.

**Methodology:** Wildtype (WT), SCA2 and SCA3 transgenic animals were divided into 2 groups: unstimulated and motor stimulated group. The cerebellum was collected to analyze transcription and translation levels of the AID, and the whole brain was collected do detect the cell type expressing the proteins.

**Results:** We found that the phosphorylation of the main transcription required for AID gene expression was decreased in transgenic animals in comparison with age-matched WT animals. Accordingly, transcriptional analysis of AID genes showed that AID1 and AID2 that had their expression 6.3 and 3.3-fold increased, respectively, and 4.4 and 3.1-fold increased, respectively. However, this induction was impaired both in SCA2 and SCA3 mouse models. Moreover, immunostaining showed which neuron type were expressing AID genes.

**Discussion:** AID1 and AID2 are differentially expressed upon stimulation in WT mice, whereas there is decreased in both SCA2 and SCA3 disease models. As well as the translation and transcription factors that regulate the transcription of these genes in impaired in models of both disorders. Finally we found that these genes are expressed in a cell-type specific manner.

**References:**

(1) Bezprozvanny I, Klockgether T. Therapeutic prospects for spinocerebellar ataxia type 2 and 3. Drugs Future. 2009;34(12). doi:10.1358/dof.2009.034.12.1443434

(2) Zhang SJ, Zou M, Lu L, et al. Nuclear calcium signaling controls expression of a large gene pool: identification of a gene program for acquired neuroprotection induced by synaptic activity. PLoS Genet. 2009;5(8):e1000604. doi:10.1371/journal.pgen.1000604

**Acknowledgments:**

This study was funded by the Portuguese Science and Technology Foundation (FCT) scholarship 2022.10161.BD.

**Declaration of Interests:** The authors declare no conflicts of interest.

#### P15 - NOVEL MECHANISMS CAUSING FAMILIAL HYPERCHOLESTEROLAEMIA: FUNCTIONAL CHARACTERIZATION OF VARIANTS IN THE REGULATORY REGIONS OF PCSK9 GENE

Ana M. Mateus^1^, Ana C. Alves^1,2^, Rafaela Lacerda^2,3^, Rafael Fernandes^2,3^, Gilles Lambert^4^, Luísa Romão^2,3^, Mafalda Bourbon^1,2^

^1^*Departamento de Promoção da Saúde e Prevenção de Doenças Não Transmissíveis, Instituto Nacional de Saúde Doutor Ricardo Jorge, 1600-609 Lisbon, Portugal;* ^2^ *BioISI-Biosystems & Integrative Sciences Institute, Faculdade de Ciências, Universidade de Lisboa, 1749-016 Lisbon, Portugal* ^3^ *Departamento de Genética Humana, Instituto Nacional de Saúde Doutor Ricardo Jorge, 1600-609 Lisbon, Portugal;* ^4^ *Laboratoire Inserm UMR 1188 DeTROI, Universite de la Reunion Plateforme CYROI, Sainte Clotilde, France.*

Familial hypercholesterolemia (FH) is a worldwide highly prevalent genetic lipid disorder, characterized by increased LDL cholesterol levels in circulation, leading to premature atherosclerosis and cardiovascular events.

Among the other main causative genes of FH, PCSK9 is a major modulating gene in cholesterol homeostasis, and PCSK9 gain of function variants cause FH.

When intracellular levels of sterols decrease, PCSK9 is positively regulated by Sterol Regulatory Element-Binding Proteins 2 (SREBP2) and Hepatic Nuclear Factor 1α (HFN1α), while LDLR transcription is only regulated by SREBP2. Statins induce the expression of both SREBP2 and HFN1α, which activate PCSK9 transcription, and LDLR transcription cannot compensate for this effect, diminishing the beneficial effects in some patients. The current challenge is to understand the physiological regulation of PCSK9 expression at the transcriptional and post-transcriptional levels.

Based on the lack of understanding of mechanisms behind the regulation of PCSK9 expression, 3 variants in the PCSK9 promoter region, close to the SRE motif, and 14 in the 5’ untranslated region (5’UTR), were studied. To this purpose, the promoter and 5’UTR regions of PCSK9 (-650 to -1) were cloned into pGL4.10[luc2] plasmid containing the Firefly luciferase (Fluc) reporter gene. After directed mutagenesis to obtain the variants in the study, they were transfected in HepG2 and Huh7 cell lines, and luciferase activity was determined by the Dual-Luciferase Reporter Assay System in a GloMax Luminometer. To simulate cholesterol starvation, 24 h hours after transfection, cells were treated with a medium supplemented with 0.1% FBS, and, after 18-24h, cells were treated with mevastatin to activate transcription.

In HepG2 and Huh7, c.-346G>C showed a decrease in promoter activity to 26% and 30% of the wild type, respectively, while c.-353G>T showed an increase to 148% and 178% of the wild type. These results indicate that c.-353G>T can possibly lead to an FH phenotype, while c.-346G>C can result in cases of hypocholesterolemia. The remaining constructs are currently undergoing research.

Expanding our understanding of how these variants affect PCSK9 synthesis through the activation of transcription factors could lead to an opportunity for personalized therapy in patients carrying these variants along with other pathogenic variants in causative genes.

**References: 1**. Mariano, C. et al. The familial hypercholesterolaemia phenotype: Monogenic familial hypercholesterolaemia, polygenic hypercholesterolaemia and other causes. Clin Genet 97, 457-466 (2020). **2**. Graça, R. et al. Characterization of Two Variants at Met 1 of the Human LDLR Gene Encoding the Same Amino Acid but Causing Different Functional Phenotypes. Biomedicines 9, 1219 (2021). **3**. Seidah, N. G. & Prat, A. The Multifaceted Biology of PCSK9. Endocrine Reviews 43, 558-582 (2022). **4**. Dong, B., Singh, A. B., Shende, V. R. & Liu, J. Hepatic HNF1 transcription factors control the induction of PCSK9 mediated by rosuvastatin in normolipidemic hamsters. International Journal of Molecular Medicine 39, 749-756 (2017).

#### P16 - EFFECTS OF METHANOLIC AND AQUEOUS EXTRACTS OF CARICA PAPAYA LEAF ON MIRNA-MEDIATED TRANSCRIPTIONAL REGULATION OF FETAL HEMOGLOBIN

Filipa Jacques, Mariana Delgadinho, Catarina Ginete, Mário Gomes, Edna Ribeiro, Miguel Brito, Anita Q. Gomes

H&TRC – Health & Technology Research Center, Escola Superior de Tecnologia da Saúde de Lisboa, Instituto Politécnico de Lisboa, Portugal

**Introduction:** Hemoglobinopathies are genetic blood disorders resulting characterized by abnormal hemoglobin that disrupts oxygen transport and leads to diverse health complications. Raising fetal hemoglobin (HbF) levels is one possible therapeutic approach. But the current main therapy based on the use of hydroxyurea (HU), faces limitations related to cost and safety, especially in underserved areas. To overcome these constraints, researchers are investigating natural alternatives like Carica papaya (CP) leaf extracts to boost HbF production with fewer side effects.

**Methodology:** We conducted a comparative study using aqueous (AECP) and methanolic (MECP) CP leaf extracts to evaluate their impact on HbF regulation in K562 myeloid cells. Gene expression changes were assessed using RT-qPCR after 24-hour exposure to varying extract concentrations (0,05 μg/ml, 0,5μg/ml and 5μg/ml).

**Results:** The transcriptional analysis of hemoglobin genes (HBA, HBB and HBG1) as well as that of its epigenetic regulators (HDAC1, HDAC2, HDAC3, DNMT1 and DNMT3b), transcription inhibitors (BCL11A and KLF1), transcription activators (MYC and HIC2) and miRNA-mediated regulators (miR-30a-3p, miR-29C-3p and miR-148b-3p) has revealed distinct expression patterns in K562 cells exposed to AECP when compared to MECP. While the expression levels of some genes were affected by one extract, other genes were affected by the other extract and some remained unaffected suggesting that the concentration and specific composition of AECP or MECP has a different impact on hemoglobin expression.

**Discussion:** The differential effects of MECP and AECP exposure on hemoglobin related genes expressed in K562 cells indicates that complex regulatory mechanisms underly their action. The concentration-dependent responses observed emphasize the need for further research to comprehend their implications in hemoglobinopathies. Overall, this study highlights the potential of extracts of CP leaf as modulators of HbF gene expression in K562 cells. Further investigation into their gene modulation mechanisms is warranted.

**Acknowledgements:** This project was supported by the grant IDI&CA-IPL/2022/miRCa/ESTeSL

#### P17 - FUNCTIONAL CHARACTERIZATION OF TWO APOB VARIANTS FROM EXON 29 FOUND IN INDIVIDUALS WITH CLINICAL DIAGNOSIS OF FAMILIAL HYPERCHOLESTEROLEMIA

Maria S. Ferreira^1^, Ana C. Alves^1,2^, Asier Larrea-Sebal^3,4,5^, César Martín^3,4^, Mafalda Bourbon^1,2^

^1^*Grupo de Investigação Cardiovascular, Unidade I&D, Departamento de Promoção da Saúde e Doenças Não Transmissíveis, Instituto Nacional de Saúde Doutor Ricardo Jorge, Lisboa, Portugal;* ^2^*BioISI – Biosystems & Integrative Sciences Institute, Faculty of Sciences, University of Lisbon, Portugal;* ^3^*Department of Biochemistry and Molecular Biology, Universidad del País Vasco UPV/EHU, 48080 Bilbao, Spain;* ^4^*Department of Molecular Biophysics, Biofisika Institute, University of Basque Country and Consejo Superior de Investigaciones Científicas (UPV/EHU, CSIC), 48940 Leioa, Spain;* ^5^*Fundación Biofisika Bizkaia, 48940 Leioa, Spain*

**Introduction:** Familial hypercholesterolemia (FH) is an inherited lipid disorder characterized by increased levels of LDL cholesterol. About 5-10% of FH cases occur due to variants in the APOB gene, but these alterations can be a more common cause of FH than expected since most of APOB variants identified is still unknown their effect on the metabolism. The majority of the variants are missense but there are a few nonsense variants and small indels in exon 29 identified in individuals with hypercholesterolemia phenotype that can cause FH.

The aim of this project was to functional characterize APOB variants from exon 29 identified in individuals referred to the Portuguese FH Study to assess if these are the genetic cause of disease.

**Methodologies:** LDL from index cases and relatives was isolated through sequential ultracentrifugation. ED-LDLR was purified from HEK293 cells transfected with the pcDNA3.1-EC-LDLR-His plasmid by affinity chromatography. Purified ED-LDLR fragments were coated onto 96-well plates and incubated with the different APOB variants. Antibodies were used for ligand detection, and absorbance was determined at 405 nm. CHO-ldlA7 cells were transfected with wt LDLR plasmid and incubated with FITC-labeled LDL to determine LDL binding and uptake by flow cytometry.

**Results:** p.(Gln4316*) and p.(Glu4387Asnfs*7) alterations from exon 29 showed reduced affinity for the LDL receptor. Uptake and binding assays results were similar, so these variants may affect the binding of apoB to the LDL receptor. The alterations studied were not present in a normolipidemic panel.

**Discussion:** APOB variants studied in this work produce truncated forms of apoB, but they are unlikely to lead to nonsense-mediated decay processes due to their location near the end of the gene. Functional studies can provide important evidence for variant pathogenicity assessment being these essential to provide an accurate diagnosis. These assays can confirm the clinical diagnosis by highlighting the cause of disease, and contribute to a personalized treatment and stratify patient associated cardiovascular risk.

#### P18 - STUDY OF THE CONTRIBUTION OF GENES NERVOUS SYSTEM RELATED TO HEART FAILURE

Mafalda Santos^1,2^, Ana Matias^1,2^, Laura Aguiar^3,4^, Mário Rui Mascarenhas^4,5^, Mário Barbosa^6,7^, Ana Melício^8^, Luiz Menezes Falcão^3,7,9^, Manuel Bicho^1,3,4^, Ângela Inácio^1,3,4^

^1^*Laboratório de Genética, Faculdade de Medicina da Universidade de Lisboa;* ^2^*Faculdade de Ciências da Universidade de Lisboa;* ^3^*Instituto de Investigação Científica Bento da Rocha Cabral, Lisboa;* ^4^*Instituto de Saúde Ambiental, Laboratório Associado Terra, Lisboa;* ^5^*Departmento de Endocrinologia, Diabetes e Metabolismo, Hospital de Santa Maria, Centro Hospitalar Lisboa Norte, Lisboa;* ^6^*Departamento de Medicina Interna, Hospital Lusíadas Lisboa;* ^7^*Faculdade de Medicina da Universidade de Lisboa;* ^8^*Serviço de Medicina II, Hospital de Santa Maria, Centro Hospitalar Lisboa Norte, Lisboa;* ^9^*Centro Cardiovascular da Universidade de Lisboa (CCUL@RISE), Faculdade de Medicina da Universidade de Lisboa*

**Introduction:** HF is a clinical syndrome due to a structural and functional abnormality of the heart that results in elevated intracardiac pressures and inadequate cardiac output at rest or during exercise. This syndrome is a major public health problem and one of the main causes of mortality. Moreover, it can be amplified by pathology associated with the Nervous System.

**Aim:** This study aims to investigate the contribution of variants in genes BDNF (rs6265), NTRK2 (rs2289656), NGF (rs6330), NOS2 (rs9282801 and rs2297518) and ADRB2 (rs1042713) to HF development.

**Material and methods:** A case-control study was conducted with a population of 508 individuals, of which 268 had HF and 240 were controls. Among the HF group, the disease was divided into non-preserved ejection fraction (EF) and preserved EF. Variants related to the NOS2 and ADRB2 genes were performed using the RFLP-PCR technique, the remaining variants were analyzed using the Genotyping Endpoint PCR technique. Statistical analysis was performed using IBM software® SPSS® Statistics 28.0, with a statistical significance level set at p < 0.05.

**Results:** For the rs2289656 variant (NTRK2), the presence of the A allele was found to be protective for HF [OR (CI, 95%) = 0.393 (0.184 – 0.837); p = 0.015]. Regarding all the other variants, no statistically significant differences were found between patients and controls. When the analysis between non-preserved EF and controls was carried out, again the presence of the A allele emerged a as protection factor [OR (CI, 95%) = 0.294 (0.116 – 0.746); p = 0.010)]. These results were adjusted for age, BMI and gender.

**Discussion:** These results show an association between the NTRK2 gene and HF in general, and non-preserved EF. The identification of genetic variants that can somehow influence the development and severity of HF may allow its faster diagnosis and the application of methodologies for disease prevention. Moreover, the results of this study will contribute to define a genomic profile associated with the role of the nervous system in HF.

#### P19 - STUDY OF THE CONTRIBUTION OF MODULATORS OF IRON HOMEOSTASIS IN HEART FAILURE

Ana Matias^1,2^, Mafalda Santos^1,2^, Laura Aguiar^3,4^, Mário Rui Mascarenhas^4,5^, Mário Barbosa^6,7^, Ana Melício^8^, Luiz Menezes Falcão^3,7,9^, Paula Faustino^4,10^, Manuel Bicho^1,3,4^, Ângela Inácio^1,3,4^

^1^*Laboratório de Genética, Faculdade de Medicina da Universidade de Lisboa;* ^2^*Faculdade de Ciências da Universidade de Lisboa;* ^3^*Instituto de Investigação Científica Bento da Rocha Cabral, Lisboa;* ^4^*Instituto de Saúde Ambiental, Laboratório Associado Terra, Universidade de Lisboa;* ^5^*Serviço de Endocrinologia, Diabetes e Metabolismo, Hospital de Santa Maria, CHLN, Lisboa;* ^6^*Serviço de Medicina Interna do Hospital Lusíadas, Lisboa;* ^7^*Faculdade de Medicina da Universidade de Lisboa;* ^8^*Serviço de Medicina II do Hospital de Santa Maria, CHLN, Lisboa;* ^9^*Centro Cardiovascular da Universidade de Lisboa (CCUL@RISE), Faculdade de Medicina da Universidade de Lisboa;* ^10^*Instituto Nacional de Saúde Doutor Ricardo Jorge, Lisboa*

**Introduction:** Heart failure (HF) is considered one of the biggest public health problems, affecting 2% of the world’s population. Is defined as a clinical syndrome due to a structural and/or functional abnormality of the heart that results in elevated intracardiac pressures and/or inadequate cardiac output at rest and/or during exercise. It can be influenced by several genetic modulators, in particular genes responsible for the balance of iron (Fe) metabolism, such as the HFE, SLC40A1 and TMPRSS6 genes.

**Aims:** To investigate the contribution of common genetic variants in HFE (C282Y - rs1800562 and H63D - rs1799945), SLC40A1 (rs1439816 and rs2304704) and TMPRSS6 (rs855791) to HF.

**Material and Methods:** The study included a population of 301 HF patients and 361 controls. The polymorphic analysis of the HFE gene variants (C282Y and H63D) was realized using the Multiplex PCR-ARMS technique, while the Endpoint Genotyping PCR technique was used for the remaining variants. Statistical analysis was done using SPSS software, version 28.0, with a statistical significance level of p<0.05.

**Results:** Statistically significant differences were found between patients and controls, in relation to the frequency of the C282Y genotypes. The presence of the Y allele [OR (CI, 95) = 3.127 (1.223-7.995); p = 0.017] was considered a risk factor for HF development.

**Discussion:** Based on the results obtained, the HFE gene was shown to modulate HF. This investigation not only provides a better understanding of the role of HFE in the etiology of HF and is a step forward in personalized medicine, but also underlines the importance of the iron homeostasis in HF. It proposes and reaffirms that the study of iron – related biomarkers as well as HFE common variants should be performed in patients with HF.

#### P20 - UNVEILING SCA2 DISEASE PHENOTYPE: A NEW MOUSE MODEL REVEALS MOTOR DEFICITS AND NEUROPATHOLOGY

André Conceição^1,2,3,4,5^, Rebekah Koppenol^1,2,3^, Inês Afonso^1,2,3^, Renato Vieira^3^, Rodrigo Paulino^3^, Lorenzo Mirapalheta^3^, Carlos A. Matos^1,3^, Clévio Nóbrega^1,3,5^

^1^*ABC-RI, Algarve Biomedical Center Research Institute, Faro, Portugal;* ^2^ *PhD Program in Biomedical Sciences, Faculdade de Medicina e Ciências Biomédicas, Universidade do Algarve, Faro, Portugal;* ^3^ *Faculdade de Medicina e Ciências Biomédicas, Universidade do Algarve, Faro, Portugal;* ^4^ *Center for Neuroscience and Cell Biology (CNC);* ^5^ *Champalimaud Research Program, Champalimaud Center for the Unknown, Lisbon, Portugal.*

**Introduction:** Spinocerebellar ataxia type 2 (SCA2) is a neurodegenerative rare disorder, characterized by an abnormal repeat of the cytosine-adenine-guanine (CAG) trinucleotide within the coding region of the human ATXN2 gene. While healthy individual typically display 22-23 CAG repetitions, when these exceed 33 repeats, people will be affected by the disease. The mutant gene is then translated into the protein ataxin-2 bearing an abnormally long tract of glutamines (polyQ). The molecular mechanisms by which the mutant ataxin-2 leads to neurodegeneration are not fully understood. However, it is known that mutant forms of ataxin-2 bearing abnormal polyQ expansion are responsible for a wide-range of cellular disturbances that include protein aggregation, RNA toxicity, enhanced oxidative stress, aberrant calcium released or autophagy impairment. People affect by SCA2, experience numerous debilitating motor symptoms that culminate in premature death. Currently, there are no therapies able to cure nor delay the natural course of SCA2 disease, and affected people rely only on symptomatic and supportive treatment. Additionally, more than 80% of all therapies that are in clinical trials fail to reach the market, mostly due to poor pre-clinical evidence. This urgent unmet medical need highlights the necessity to create new and more refined tools to studies disorders, in order to develop novel, better and more sustainable therapies. While several SCA2 mouse models were already developed, they fail to recapitulate the disorder phenotype, either because disease-related motor symptoms are mild and late stage, or neurodegeneration is not present.

**Methodology:** Fallowing this rational, and to advance our understanding of SCA2, we have developed a novel transgenic mouse model that expresses a human form of the ATXN2 gene, bearing 129 CAG repeats (that codes for 129 glutamine), under the control of the Purkinje cell-specific promoter L6-L7. We characterized four different SCA2 ATXN2-129Q mouse lines, which were submitted to a battery of behavioural test and neuropathology histological analysis for a period of 1 year, at different time points.

**Results:** Preliminary results, indicate that transgenic SCA2 ATXN2-129Q mice have motor deficits and present neurodegeneration, since early age. This novel SCA2 model will open the possibility to assess new therapeutic approaches and unveil pathophysiological mechanisms underlying this highly debilitating disorder.

#### P21 - INFLUENCE OF GENETIC VARIANTS OF OXIDATIVE STRESS RELATED GENES ON PROGNOSIS AND THERAPY RESPONSE IN CHRONIC MYELOID LEUKEMIA

Raquel Alves^1,2,3,4^, Filipa Ventura^1,2^, Joana Jorge^1,2,3,4^, Ilda P. Ribeiro^2,3,4,5^, Gilberto Marques^6^, Margarida Coucelo^2,3,4,7^, Joana Diamond^8^, Paulo Freitas-Tavares^7^, António Almeida^9,8,10^, Ana Cristina Gonçalves^1,2,3,4^, Ana Bela Sarmento-Ribeiro^1,2,3,4,7^

^1^ *Laboratory of Oncobiology and Hematology (LOH) and University Clinic of Hematology, Faculty of Medicine (FMUC), University of Coimbra, Coimbra, Portugal;* ^2^ *Coimbra Institute for Clinical and Biomedical Research (iCBR)—Group of Environmental Genetics of Oncobiology (CIMAGO), FMUC, University of Coimbra, Coimbra, Portugal;* ^3^ *Center for Innovative Biomedicine and Biotechnology (CIBB), Coimbra, Portugal;* ^4^ *Clinical Academic Center of Coimbra, CACC, Coimbra, Portugal;* ^5^ *Cytogenetics and Genomics Laboratory, Institute of Cellular and Molecular Biology, Faculty of Medicine, University of Coimbra, Coimbra, Portugal.* ^6^ *Clinical Pathology Service, Centro Hospitalar e Universitário de Coimbra (CHUC), Coimbra, Portugal.* ^7^ *Hematology Service, Centro Hospitalar e Universitário de Coimbra (CHUC), Coimbra, Portugal.* ^8^ *Hemato-Oncology Laboratory, Instituto Português de Oncologia de Lisboa Francisco Gentil EPE, Lisbon, Portugal.* ^9^ *Hospital da Luz Lisboa and Faculdade de Medicina, Universidade Católica Portuguesa, Lisbon, Portugal.* ^10^ *CIIS (Centro de Investigação Interdisciplinar em Saúde), Universidade Católica Portuguesa de Lisboa, Lisbon, Portugal.*

Chronic myeloid leukemia (CML) is a myeloproliferative neoplasm characterized by the presence of BCR-ABL1 oncogene. Oxidative stress conditions are crucial in tumor development and progression. The NRF2, codified by NFE2L2 gene, plays a central role in redox balance, promoting the transcription of cytoprotective and antioxidant genes such as SOD2, GPX1 and CAT. The action of this transcription factor is regulated by KEAP1. Alterations in these genes, either due to somatic mutations or genetic variants (SNV), have been associated with the development, prognosis, and therapeutic response in cancer.

This work evaluated the influence of polymorphisms in genes associated with oxidative stress (NFE2L2, KEAP1, SOD2, GPX1, and CAT) in prognosis and treatment response with tyrosine kinase inhibitors (TKI) in CML patients.

In 194 CML samples, nine genetic variants in NFE2L2 (rs6721961, rs4893819, rs35652124, rs6706649 and rs13001694), KEAP1 (rs113540846), SOD2 (rs4880), GPX1 (rs1050450) and CAT (rs1001179) were genotyped by PCR based assays. The association of these genetic variants with clinical-laboratory characteristics were assessed by Fisher’s test, logistic regression, and Kaplan-Meier curves, considering a p<0.05.

The results obtained showed that patients with G allele on GPX1 rs1050450 and KEAP1 rs113540846 variants have an approximately 2-fold and 31-fold higher probability of being TKI resistant, respectively. In TKI-resistant group, patients with CT genotype in NFE2L2 rs4893819 variant were more likely to need three or more lines of treatment (OR=5.60, 95%CI 1.22-25.75, p=0.027). An important factor for TKI response is BCR-ABL1 mutational status, and we found that individuals with AG genotype in NFE2L2 rs13001694 present, approximately 9x more risk of mutation on this fusion gene. Moreover, patients heterozygous for the NFE2L2 rs4893819 (CT genotype) and SOD2 rs4880 (AG genotype) variants have faster disease progression [NFE2L2: hazard ratio (HR)=7.84, 95% CI 1.82-29.93, p=0.020; SOD2: HR=7.19, 95% CI 1.62-31.96, p=0.035] comparing with other genotypes. While the GG genotype of the NFE2L2 rs13001694 variant has a 2-fold lower overall survival than other patients (HR=11.86, 95% CI 1.39-1000.7, p=0.023). Additionally, we also observed an association between haplotypes and genotypic profile with CML prognosis.

In conclusion, our results suggest that genetic variants in genes associated with the redox state might be important factors in the prognosis, survival, and therapy response in CML patients.

#### P22 - REGULATION OF THE ALTERNATIVE SPLICING OF RAC1B IN TUMOR CELLS

Inês Bizarro^1,2,3^, Peter Jordan^1,2^, Vânia Gonçalves^1,2^

^1^*Department of Human Genetics, National Institute of Health “Doutor Ricardo Jorge”, Lisbon, Portugal;* ^2^ *BioISI-Biosystems & Intergrative Sciences Institute, Faculty of Sciences, University of Lisbon, Portugal;* ^3^ *Department of Chemistry and Biochemistry, Faculty of Sciences, University of Lisbon, Portugal.*

**Introduction:** Cancer is a molecularly heterogeneous disease that presents genetic modifications in different alternative pathways. Our group has contributed to identify a subgroup of colon tumors, characterized by the simultaneous presence of an oncogenic mutation in BRAF and overexpression of RAC1B, a splicing variant of the GTPase RAC1. Together, these two changes stimulate signaling pathways that promote the proliferation and survival of colorectal cells. The overexpression of RAC1B has also been identified in pancreatic, breast, lung and thyroid cancer. This splicing variant could be a therapeutic target for the treatment of patients with certain tumour subtypes, which highlights the importance of studying its regulation in human cells. But how does the overexpression of a splicing variant occur in tumour cells? Our group found that the inclusion of alternative exon 3b, which gives rise to the variant protein RAC1B with 19 additional amino acids, depends on two splicing factors, SRSF1 (former SF2) and SRSF3 (former SRP20), which promote and inhibit, respectively, the inclusion of this exon in colorectal cancer cells. More recently, the ESRP1 factor has also been studied in these cells and found to promote RAC1B expression (unpublished data). Here we studied whether the 3 splicing factors mentioned above are also modulators of the alternative splicing of RAC1B in breast and lung cancer cells.

**Methodology:** We overexpressed the 3 splicing factors by co-transfection with a RAC1 minigene and analyzed the expression levels of the RAC1 minigene-derived transcripts. Next, we silenced the endogenous expression of the factors by siRNA and evaluated the endogenous levels of RAC1B transcript and protein.

**Results and Discussion:** After confirming the expected results in colorectal cancer cells, we were able to successfully induce the overexpression and silencing of the 3 splicing factors in breast cancer cells. A similar role for SRSF1 and SRSF3 in positively and negatively modulating RAC1B alternative splicing, respectively, was found in breast cancer cells. Intriguingly, ESRP1 promoted the skipping of exon 3b in these cells, revealing an opposite role in the alternative splicing of RAC1B comparing to what was previously observed in colorectal cancer cells. Currently, we are extending this study to lung cancer cells. These results contribute to a better understanding of the cellular specificity of alternative splicing mechanisms in general, and to the regulation of RAC1B in breast cancer in particular.

#### P23 - IL-6 INDUCES THE OVEREXPRESSION OF GENES INVOLVED IN THE REGULATION OF APOPTOSIS AND CELL-CELL ADHESION IN POLARIZED COLORECTAL CANCER CELLS

Bárbara Alves^1^, Joana F. Pereira^1,2^, José Ferrão^1^, Luís Vieira^1,3^, Paulo Matos^1,2^, Peter Jordan^1,2^, Vânia Gonçalves^1,2^

^1^*Departamento de Genética Humana, Instituto Nacional de Saúde Doutor Ricardo Jorge, 1649-016 Lisbon, Portugal;* ^2^ *BioISI – Instituto de Biosistemas e Ciências Integrativas, Faculdade de Ciências, Universidade de Lisboa, 1749-016 Lisbon, Portugal;* ^3^ *Centro de Toxicogenómica e Saúde Humana (ToxOmics), NOVA Medical School | Faculdade de Ciências Médicas, Universidade Nova de Lisboa, 1169-056 Lisbon, Portugal*

**Introduction:** An inflammatory microenvironment was identified as a critical tumor-promoting condition for cells harboring tumor-initiating mutations. Cancer cells respond to pro-inflammatory signals with changes in their transcriptome, namely upregulating the expression of pro-tumorigenic transcript variants. A paradigmatic example is the variant RAC1B. We found increased RAC1B levels in samples from inflammatory bowel disease patients or following experimentally-induced acute colitis in mouse models and showed it to be overexpressed in colorectal tumors. Recently, we found that the overexpression of RAC1B in polarized Caco-2 colorectal cancer (CRC) cells was triggered by the presence of pro-inflammatory interleukin (IL)-6. Here we describe the use of RNA-seq to characterize the transcriptome-wide changes induced in CRC cells by exposure to IL-6.

**Methods:** Total RNA isolated from control and IL-6-stimulated cells was subjected to library preparation and paired-end sequencing. Sequencing fastq files were first processed and quality controlled. Salmon was used to align raw reads to the human reference transcriptome (hg38) and to perform transcript quantification. Differentially expressed genes (DEGs) were identified using DESeq2, by analyzing the summarized counts per gene comparing control and IL-6 stimulation conditions. We have also performed an analysis for biological process enrichments by Gene Ontology. Selected DEGs in significantly enriched categories were validated by semiquantitative (sq)RT-PCR.

**Results and Discussion:** We show that the most significant IL-6-induced transcriptional changes were associated with the upregulation of a cluster of 18 genes, mostly associated with the regulation of apoptosis and CEACAM-mediated cell-cell adhesion. Using sqRT-PCR, we validated the upregulation of three of these genes in IL-6 treated Caco2-cells, but, interestingly, found their expression to be instead downregulated in primary tumors of the CRC TCGA dataset.

**Conclusion:** Exposure of polarized Caco-2 CRC cells to IL-6 causes upregulation of pro-tumorigenic genes, which, paradoxically, appear to be downregulated in primary CRC tumors. Further studies will be required to clarify the impact of these results in CRC development.

#### P24 – PERMISSION TO PUBLISH NOT GRANTED BY THE AUTHORS

#### P25 - TRNA EPITRANSCRIPTOME DISRUPTION OCCURS IN ALZHEIMER’S DISEASE IN RESPONSE TO PROTEIN AGGREGATION BURDEN, AND REPRESENTS A PROMISING THERAPEUTIC TARGET TO RECOVER PROTEOSTASIS IN NEURODEGENERATION

Marisa Pereira^1^, Stefanie Kaiser^2^, Miguel Moutinho^3^, Ana R. Soares^1^

^1^*Department of Medical Sciences, Institute of Biomedicine (iBiMED), University of Aveiro, Portugal;* ^2^ *Institute of Pharmaceutical Chemistry, Goethe-University, 60438 Frankfurt, Germany;* ^3^ *Stark Neurosciences Research Institute, Indiana University School of Medicine, Indianapolis, IN, USA.*

Alzheimer’s Disease (AD) is a challenging neurodegenerative disorder, characterized by the accumulation of β-amyloid aggregates and loss of neuronal proteostasis. The maintenance of proteostasis requires proper transfer RNA (tRNA) modifications, which play a pivotal role in ensuring optimal translation of the genetic information. These modifications are catalyzed by tRNA modifying enzymes that are often mutated or dysregulated in neurological disorders. However, not much is known concerning the relevance of tRNA modifying enzymes and tRNA modifications in the context of AD.

Here, we show that the expression of several tRNA modifying enzymes is impaired in the brains of AD patients at the transcriptional level. We further show, for the first time, that the expression of the specific tRNA modifying enzyme ELP3 negatively correlates with the amyloid plaque burden in AD patients and that ELP3 is consistently found dysregulated in different animal and cellular models of AD. Our data further demonstrate that decreased ELP3 expression in AD-neuronal like cellular models leads to tRNA hypomodification, dysregulation of the tRNA pool and translation slowdown in an attempt to counteract the deleterious effects of previous accumulation of β-amyloid aggregates. Indeed, we were able to show that accumulation of β-amyloid aggregates is the trigger for ELP3 expression disruption and tRNA epitranscriptome reprograming in AD. Of note, we were able to recover translation efficiency and proteostasis after correcting tRNA deficiencies by tRNA transfection.

In summary, our findings strongly suggest that amyloid pathology disrupts neuronal proteostasis by reducing ELP3 expression, tRNA modification levels, and consequently translation efficiency. This study underscores the therapeutic potential of modulating the tRNA epitranscriptome to reinstate neuronal proteostasis in AD, thereby preserving overall neuronal function.

**Acknowledgements:** Research funded by the FCT, POCH, FEDER, and COMPETE2020 (SFRH/BD/135655/2018, POCI-01-0145-FEDER-029843, CEECIND/00284/2018), the EU (H2020-WIDESPREAD-2020-5 ID-952373), and the Alzheimer’s Association (AARG-NTF-23-1149641).

#### P26 - PRECISION GENOME ANALYSIS: UNRAVELING SNVS AND CNVS WITH A MULTI-VARIANT CALLER WGS WORKFLOW

Marta Ferreira^1,2,3,4^, Celina São José^2^, Francisco Almeida^1,2,4^, Joaquín Maqueda^1,2^, Rita Monteiro^1,2,5^, Pedro Ferreira^1,2,4^, Carla Oliveira^1,2,6^

^1^ *Instituto de Investigação e Inovação em Saúde, Universidade do Porto, Porto, Portugal;* ^2^ *Institute of Molecular Pathology and Immunology of the University of Porto, Porto, Portugal;* ^3^ *Doctoral Program in Computer Sciences, Faculty of Sciences, University of Porto, Porto, Portugal;* ^4^ *Department of Computer Science, Faculty of Science, University of Porto, Porto, Portugal;* ^5^ *Currently at: Inovretail, SA, 4200-355 Porto;* ^6^ *Dept. of Pathology, Faculty of Medicine, University of Porto, 4200 - 319 Porto, Portugal*

**Introduction:** Exome analyses fail to detect single nucleotide variants (SNVs) or structural variants (SV) occurring outside the coding sequence. Genome-wide analysis emerges as a solution to understand genome variation, and Whole Genome Sequencing (WGS) the preferred method for that purpose. We aimed at developing a WGS analysis workflow using a combination of multiple variant callers for SNV, CNV and SV calling.

**Methods:** We used a gold standard sample from the Genome in a Bottle project (GIAB) to access the performance of a novel pipeline encompassing alignment (BWA mem); post-processing (GATK tools); CNV (integration of LUMPY, Delly and GRIDSS caller outputs) or SNV (integration of HaplotypeCaller-GATK (HC) and DeepVariant (DV) outputs) calling; and Merging of multiple called variants (overlap analysis of SNV, CNV and SV calls). We calculated recall, precision and F1 scores by comparing our outputs with those from GIAB for SNVs. As no gold standard is available in GIAB for CNVs, we compared our CNV outputs with a GIAB pool of high confidence and with variant calls from Manta, to improve our pipeline.

**Results:** We called 4.309.554 SNVs with DV and 4.578.886 with HC, and 4.109.099 were common. The performance of DV alone (F1 score=0.9819, 3.802.474 true positives) was better than SNV calling with HC alone (F1 score=0.9522, 3.759.774 true positives), but worse than the combination of DV and HC outputs (F1 score=0.9841, 3.836.476 true positives variants). Our CNV calling pipeline called a higher number of variants than the, as well as a set of inversions confirmed by visualization in samplot and supported paired-end and/or split-reads, that were not called by GIAB or Manta.

**Conclusion:** Our WGS-pipeline shows high performance and improves the likelihood of finding true positive germline SNVs, captures high confidence CNVs and uniquely calls inversions.

**Acknowledgments:** MF’s PhD fellowship Ref. 2020.05763.BD, GenomePT 22184, LEGOH PTDC/BTM-TEC/6706/2020, SOLVE-RD H2020-SC1-2017, PDCC - Faculty of Science, University of Porto

#### P27 - IN VITRO FUNCTIONAL STUDIES OF VARIANTS IN THE LDLR GENE

Inês Rodrigues^1^, Maria S. Ferreira^1^, Ana M. Medeiros^1,2^, Joana R. Chora^1^, Mafalda Bourbon^1,2^, Ana C. Alves^1,2^

^1^ *Unidade de I&D, Grupo de Investigação Cardiovascular, Departamento de Promoção da Saúde e Prevenção de Doenças Não Transmissíveis, Instituto Nacional de Saúde Doutor Ricardo Jorge, Lisboa, Portugal;* ^2^ *BioISI – Byosystems & Integrative Sciences Institute, Faculdade de Ciências, Universidade de Lisboa, Lisboa, Portugal.*

**Introduction:** Familial Hypercholesterolemia (FH) is a semi-dominant disease characterized clinically by an increase in plasma LDL cholesterol levels, leading to its accumulation mainly in tendons (tendinous xanthomas) and arteries. Genetically, this pathology is characterized by variants in three genes: LDLR, APOB and PCSK9. The majority are found in the LDLR gene. The functional characterization of variants found in the genes associated with FH is important to increase the scientific knowledge of the receptor function.

In the Portuguese Familial Hypercholesterolemia Study (PFHS), 163 LDLR alterations were found in 416 index patients and their relatives up to date. Until now, 103 of these alterations already have a final classification of pathogenic or likely pathogenic and 15 have been proved by in vitro studies to be non-pathogenic. The aim of the present work is to functionally characterize 4 LDLR missense alterations found in Portuguese FH patients and worldwide.

**Methods:** Four LDLR mutants were generated by site-directed mutagenesis and expressed in CHO–ldlA7 cells lacking endogenous expression of LDLR. To determine the effects of alterations on LDLR function, cell surface expression and binding and uptake of FITC-LDL was assessed by flow cytometry and western blot.

**Results:** The variant p. (Pro608His) show a very low LDLR activity (binding, internalization of LDL and expression <2%). In the p. (Phe403del) variant, we observed an LDLR activity of ~20%. The p. (Pro608Ser) variant showed a decrease in the binding and internalization of LDL (30-50%) and normal expression. p. (Val827Ile) variant does not alter the LDLR function (binding, internalization of LDL and expression >90%).

**Discussion:** Functional studies increase the knowledge about how variants affect the LDLR cycle. Functional characterization of LDLR variants is important for a correct variant classification, which will, in turn, influence patients’ diagnosis, treatment and, in the end, patient prognosis. Moreover, it contributes to the clarification of the molecular basis of FH worldwide.

#### P28 - GENANIMATED: INNOVATIVE GENETICS EDUCATION GOES TO THE MOVIES

Marisa Silva

Instituto Nacional de Saúde Dr. Ricardo Jorge, Departamento de Genética Humana, Lisboa, Portugal

Despite living in a flourishing era of Genetics and Genomics, its perception by the Portuguese general public is still limited. Furthermore, personalized treatments based on genetic testing/information will become increasingly available in upcoming years. Knowledge of genetics/genomics concepts is therefore crucial to understand recent developments and to make decisions based on accurate and trustworthy information.

GenAnimatEd is a series of 3D animation videos on Genetics and Genomics and their potential impact on human health. Its focus is to communicate complex concepts, technologies/applications, and research projects, in a clear and plain language, to students, teachers, educators, non-geneticists healthcare professionals (HCPs), and society in general.

Presently, the series comprises 17 digital animations, each addressing a specific subject – from DNA to proteins – or area of expertise – from Cytogenetics to Proteomics – and their potential impact on human health. Additional episodes focus on specific research projects, their aims, methodology, and results (obtained or expected) and how they may affect patients/healthcare users. The whole series is freely available as a playlist on our Institute’s YouTube channel to increase outreach.

Combined visualizations have surpassed 5000 views, a high number of which occurred in classrooms, 4 communication symposiums, and 5 national and international science/film festivals, leading to an even wider outreach, both in number and type of audience. Despite targeting mainly high-school and university students, as well as educators and HCPs, increasingly positive qualitative feedback has been obtained from non-targeted and diverse groups.

The successful outreach of our series confirms that animation videos are a high-quality innovative format that improves health communication to a widespread audience. It also acts as an accessible and appealing tool to fight misinformation. This contributes to a more informed vision and greater understanding of the importance and potential impact of genetic information in healthcare, which is crucial for health decisions pertaining disease diagnosis, treatment, and management of patients and their families.

#### P29 - A MACHINE LEARNING-BASED ALTERNATIVE FOR THE IDENTIFICATION OF CANDIDATE GENES FOR SCHIZOPHRENIA

Daniel Martins^1,2^, Maryam Abassi^1,3^, Joel P. Arrais^1^, Conceição Egas^2,4^

^1^*University of Coimbra, Centre for Informatics and Systems of the University of Coimbra, Department of Informatics Engineering, Coimbra, Portugal;* ^2^ *University of Coimbra, Centre for Innovative Biomedicine and Biotechnology., Coimbra, Portugal;* ^3^ *Polytechnic Institute of Coimbra, Applied Research Institute, Coimbra, Portugal;* ^4^ *Biocant – Transfer Technology Association, Cantanhede, Portugal*

**Introduction:** Heritability studies have indicated a substantial contribution of genetic factors to the manifestation of Schizophrenia (SCZ). However, it still lacks definitive hypotheses to explain its etiology. This uncertainty forces SCZ diagnosis to rely on broad frameworks, which, in turn, hinders the collection of robust case-control cohorts. As a paradigmatic example of this issue, one of the largest cohorts on SCZ was drawn from the Swedish Hospital Discharge Register, for which there have been reported relatively high SCZ misdiagnosis rates (ranging from 6% to 19%).

**Methodology:** This large-scale case-control Whole-Exome Sequencing dataset from the Swedish population was reduced to 18,970 variants with significant associations to the phenotype. A gene-annotation-based Machine Learning model was built and trained on the entire data to enhance the within-group distinctions. The outlying cases, associated with a higher likelihood of misclassification, were excluded from the subsequent analysis. A second iteration of the model was trained to analyze the capability of the refined dataset to identify candidate genes for Schizophrenia.

**Results:** After excluding samples on a proportion equivalent to the misdiagnosis rate associated with a narrow SCZ definition (19%), the classification model presented an AUC value of approximately 0.81 for the test set. This degree of separability between cases and controls would be in line with Schizophrenia heritability estimates from the existing literature. Further analyses point to a greater weight of contribution of genes on glucose metabolism pathways

**Discussion:** Our findings suggest that the reported misdiagnosis rates for Schizophrenia may be reflected on its case-control cohorts. This could compromise the performance of purely genetic studies relying on such datasets. In comparison to the original data, the genetic profiles leading to the distinction of cases and controls in the refined dataset were more consistent across different replications of the training process. This supports the presented strategy to define more informative subsets from large-scale cohorts and opens the door to reusing such data with adapted machine learning approaches for complex disease research.

**Acknowledgements:** FCT SFRH/BD/146094/2019; UIDB/ 00326/2020; UIDP/00326/2020; UIDB/04539/2020; UIDP/04539/2020; LA/P/0058/2020; dbGaP phs000473.v2.p2.

### Clinical Research

#### P30 - FIRST CLINICAL GENETIC STUDY OF INTELLECTUAL DISABILITY IN THE PEOPLE’S REPUBLIC OF ANGOLA

Roberto Lardoeyt Ferrer^1^, Maria do Rosário Bragança^2^, Fernando Alberto Quilezi^2^, Vanilson de Oliveira Porto Borges^2^, Bárbara Tchissola Sanjulo da Rocha Quilezi^3^, Marisa Teca Nuno^4^, Vicelma Mateus Vicente Ferreira-Borges^2^, Albertino Candimba Sebastião^1^

^1^ *Instituto Superior Politécnico Alvorecer da Juventude (ISPAJ). Luanda, Angola.* ^2^ *Universidad Katyavala Bwila, Facultad de Medicina. Benguela, Angola.* ^3^ *Hospital General de Benguela. Benguela, Angola.* ^4^ *Hospital Municipal de Bocoio. Benguela, Angola.*

**Introduction:** The American Association on Mental Retardation characterizes intellectual disability (ID) as significant limitations in intellectual functioning and adaptive behavior (practical, social, and conceptual skills), beginning before the age of 18.(1) The etiology of ID is multiple; more than 250 causes that originate it have been identified. According to the American Association of Mental Retardation (AAMR), etiological factors can have an origin: prenatal (whether genetic or environmental), perinatal and postnatal.(2,3)

**Objective:** To characterize the causes of intellectual disability in a sample of institutionalized children at the Special School in Benguela in the People’s Republic of Angola.

**Methodology:** A cross-sectional descriptive observational study was carried out on a population of 139 schoolchildren with ID. Guvtavson etiopathogenic classification was applied to define the cause. Genetic causes were also identified.

**Results:** Prenatal etiology was the most frequent (45.3%), followed by postnatal (29.5%) and perinatal ones (18.0%). Environmental etiology (20.9%) was the most prevalent in the prenatal category, highlighting alcohol consumption (8.6%). In the perinatal stage, hypoxia at birth (5.0%) stood out. In the postnatal stage, cerebral malaria (15.1%) was the first cause, being also the third most predominant among all categories. In addition, 80% of the cases studied had at least one relative with the same disability. Thirteen cases with genetic syndromes were identified, four cases with chromosomal aberrations and six with a multifactorial disorder.

**Discussion:** In Angola, a prenatal care strategy should be implemented from the genetic point of view, continuing to insist on prenatal, perinatal and postnatal care for mothers and children. In addition, genetic counseling services should be implemented to help increase genetic education in the Angolan population, and frequent infections such as Malaria, both in the prenatal and postnatal stages, should be controlled. All these elements can enrich the idea of implementing community genetics supported by a technological support.

**References:**

1. Campo BA, Hernández FA, Pérez VA, Toledo GGC, Fernández Perrone AL. Discapacidad intelectual. Protoc diagn ter pediatr [Internet]. 2022 [Citado 05/03/2023];1:51-64. Disponible en: https://www.aeped.es/sites/default/files/ documentos/06.pdf

2. Fioravante Diniz NL, Parlato-Oliveira E, Ayres Pimenta PG, Arantes de Araújo L, Ribeiro Valadares E. Autism and Down syndrome: early identification and diagnosis. Arq Neuropsiquiat [Internet]. 2022 [Citado 05/03/ 2023];80(6):62030. Disponible en: https://pubmed.ncbi. nlm.nih.gov/35946706/

3. Lantigua CA, Portuondo SM, Mesa CT, Lardoeyt-Ferrer R. Epidemiology of Prenatal Genetic and Environmental Factors of Mental Retardation in Cuba. MEDICC Review [Internet]. 2008 [Citado 05/03/2018];10(1):29-36. Disponible en: https://pubmed.ncbi.nlm.nih.gov/21483354/

**Conflict of interest**: The authors declare that they have no conflict of interest with the preparation and dissemination of this work and the information it contains.

#### P31 - DISCLOSURE OF GENETIC RISK INFORMATION TO FAMILY MEMBERS: PREFERENCES OF INDIVIDUALS FROM FAMILIES WITH INHERITED CONDITIONS IN PORTUGAL

Mara Pinto^1^, João P. Freixo^2^, Filipa Júlio^3^, Tamara H. Milagre^4,5^, Liliana Sousa^1^, Luís Sousa^6^, Álvaro Mendes^2,7^

^1^ *Department of Education and Psychology, CINTESIS@RISE, Universidade de Aveiro, Aveiro, Portugal;* ^2^ *CGPP – Centre for Predictive and Preventive Genetics, IBMC – Institute for Molecular and Cell Biology, i3S – Instituto de Investigação e Inovação em Saúde, Universidade do Porto, Porto, Portugal;* ^3^ *Associação Portuguesa dos Doentes de Huntington, Lisboa, Portugal;* ^4^ *Associação Evita – Cancro Hereditário, Lisboa, Portugal;* ^5^ *ePAG ERN GENTURIS - European Patient Advocacy Group, European Reference Network for all Patients with one of the Rare Genetic Tumour Risk Syndromes;* ^6^ *APAHE – Associação Portuguesa de Ataxias Hereditárias, Vila Franca de Xira, Portugal;* ^7^ *UnIGENe, IBMC – Institute for Molecular and Cell Biology, i3S – Instituto de Investigação e Inovação em Saúde, Universidade do Porto, Porto, Portugal*

Communicating genetic risk to patients’ relatives is key to realizing the full benefits of genetic health care. Standard practice involves healthcare professionals (HCPs) assisting patients in identifying at-risk relatives and encouraging disclosure, with patients bearing the responsibility for informing relatives of their genetic risk. However, this communication is often challenging and genetic counselling and testing among at-risk relatives are underutilized. This study aimed to explore the preferences of individuals from families with inherited genetic conditions about the disclosure of genetic risk to family members.Seventeen patients’ organizations in Portugal advertised online surveys between November 2022 and January 2023. Data were analyzed through descriptive and comparative statistics.

292 participants were considered: mean age 45.5 (range: 18-77, SD 11.54), 74.7% were female, 7.2% had children, 59% had higher education. Participants comprised 45.4% symptomatic individuals, 26% direct relatives, 21.4% pre-symptomatic carriers, and 7,2% non-biological family members, mainly from families with hereditary ATTR amyloidosis (15%) Huntington’s disease (14%), cystic fibrosis (13%), and hereditary cancer predisposition syndromes (11%). Most participants (39%) believed that all relatives should be informed, 32% thought that direct relatives should be contacted first, and 26% believed that only direct relatives should be informed. A substantial number (45%) believed that probands should initiate the first contact, with HCPs following up; while 28% thought that probands should decide whether HCPs would be involved in informing relatives. A significant majority (84%) believed that HCPs should actively offer support for inform relatives. Moreover, 83.6% felt that HCPs should directly inform relatives when patients are unable or unwilling to do it, for treatable or untreatable conditions.

These results highlight preferences for involving patients and HCPs in informing relatives of their genetic risk, including direct contact by genetics services. This study contributes for discussions on how to appropriately cascade relevant information to at-risk relatives.

#### P32 - THE CLINICAL APPROACH IN CASE OF DPYD MUTATION, AT THE PORTUGUESE INSTITUTE OF ONCOLOGY OF COIMBRA

Filipa P. Freitas^1^, Joana Oliveira^1^, Marta Mota^1^, Joana Rosmaninho-Salgado^2^, Ana. R. Paiva^1^, Rui Soares^1,3^

^1^*Clinical Pathology Service, Portuguese Institute of Oncology of Coimbra Francisco Gentil, EPE;* ^2^ *Serviço de Genética Médica, Hospital Pediátrico de Coimbra, CHUC;* ^3^ *Institute of Medical Microbiology, Faculty of Medicine, University of Coimbra*

**Introduction:** Personalizing pharmacotherapy has emerged as a pivotal approach for enhancing drug efficacy while minimizing adverse effects. Fluoropyrimidines (FP) is a drug used in the treatment of some cancers1. Dihydropyrimidine dehydrogenase (DPD) is a crucial enzyme in the metabolism of FP2. In Portugal due to DPYD variants, European Medicines Agency and Infarmed recommended the testing of the four recommended variants (DPYD*2, DPYD*13, HapB3, and c.2846A>T) before the beginning of treatments3. In our hospital, usually, clinicians prescribed the FP in reduced doses before the diagnosis independently of DPYD genotype. The DPYD-genotype was established since 2021, therefore this work intends to evaluate if testing for DPYD-genotype had an impact on dose individualization in daily clinical care.

**Methodology:** The blood of 320 oncologic patients from the Portuguese Institute of Oncology of Coimbra Francisco Gentil was collected. Genomic DNA and the detection of the variants was through real-time polymerase chain reaction using gb PHARM DPYD kit according to the manufacturer’s instructions.

**Results:** Of 320 patients who were submitted to the DPYD test, 3% presented one of tested variants, in heterozygosity: c.2846AT (n=4), DPYD*2A (n=3), and HapB3 (n=3). Four of these patients had already initiated treatment before undergoing DPYD-genotype test, one passed away before the treatment, one had to be suspended, and one did not present clinical features to do the treatment. The other three patients did the full treatment, where in two (66%) it was reduced 50% of the dose and only one maintained the initial dosage of FP.

**Discussion:** The 3% of DPDY variants is according to the literature2. Although a small number of patients benefit from DPYP variants, this study supports the clinical utility of DPYD genotyping as a screening test for DPD deficiency. A previous diagnosis of DPDY genotype could prevent the toxicity and a bigger damage to the health of the patients.

**Declaration of interests:** The 1st and the 2nd author rely on the sponsorship of Bioportugal for the costs of event registration

**References**:

1 Lau et al., BMC cancer, 2023, 23.1: 380

2 Lunenburg et al., Eur J Hum Genetics, 2020, 28.4: 508-517

3 Circular Informativa DGS N.º 072/CD/550.20.001; 18/03/2020

#### P33 - PROP1 MUTATIONS IN PORTUGUESE PATIENTS WITH COMBINED PITUITARY HORMONE DEFICIENCY

Ana C. Ribeiro^1^, Eduarda Coutinho^1^, Catarina I. Gonçalves^1^, Luís R. Saraiva^2^, Manuel C. Lemos^1^

^1^*Unidade de Genética Médica, Centro de Genética Médica Doutor Jacinto Magalhães, Centro Hospitalar Universitário de Santo António, Porto, Portugal.* ^2^ *Life and Health Sciences Research Institute (ICVS), School of Medicine, University of Minho, Braga, Portugal;* ^3^ *ICVS/3B’s – PT Government Associate Laboratory, Braga/Guimarães, Portugal.* ^4^ *Unit for Multidisciplinary Research in Biomedicine, Abel Salazar Biomedical Sciences Institute, Porto University, Porto, Portugal.* ^5^ *Laboratório de Citogenética, Instituto de Ciências Biomédicas Abel Salazar (ICBAS), Universidade do Porto, Porto, Portugal.*

**Keywords**: PROP1, Combined Pituitary Hormone Deficiency, mutation, pathogenic variant.

**Introduction:** Combined pituitary hormone deficiency (CPHD) is characterized by impaired secretion of growth hormone and one or more other anterior pituitary hormones. Genetic CPHD can be caused by mutations in genes involved in the hypothalamic-pituitary development. Particularly, inactivation of PROP1, which encodes a pituitary-specific transcription factor, was firstly related to CPHD in 1998. Since then, PROP1 become recognized as the most common genetic cause for this disorder. Our aim was to establish the prevalence of PROP1 mutations in a cohort of Portuguese patients with idiopathic CPHD.

**Methodology:** This study included 119 CPHD patients, consisting of 98 sporadic cases and 21 familial cases. The PROP1 gene was analysed by Sanger sequencing. Putatively pathogenic variants were selected by keeping non-synonymous variants located in the exons and intron-exon boundaries, along with an allele frequency below 0.001 in gnomAD.

**Results:** Homozygous or compound heterozygous PROP1 mutations were identified in 30 (25.2%) patients with CPHD. These were present in 9 (9.2%) of sporadic cases and 21 (100%) of familial cases. The most frequently observed mutation was the homozygous deletion c.301_302delAG, p.(Leu102Cysfs*8), present in 24 (20.2%) CPHD patients.

**Discussion:** PROP1 mutations were identified in 25.2% of Portuguese patients with idiopathic CPHD. In accordance with previous studies, we observed a high frequency of the c.301_302delAG deletion. This first reported mutation remains the most frequent PROP1 pathogenic variant reported so far.

**Acknowledgements:** This work was funded by Portuguese Foundation for Science and Technology (PTDC/SAU-GMG/098419/2008 and UI/BD/151021/2021).

#### P34 - LEARNING IS A LIFELONG EXPERIENCE: LESSONS FROM 30 YEARS OF THE PORTUGUESE FANCONI ANEMIA COHORT

Jorge Diogo Da Silva^1,2,3,4^, Cláudia Oliveira^5^, Ana Rita Soares^1,4^, Isabel Serra Nunes^1,4^, Nádia Neto^5^, Ana Maria Fortuna^1,4^, Nataliya Tkachenko^1,4^, Beatriz Porto^5^

^1^*Serviço de Genética Médica, Departamento de Pediatria, Hospital de Santa Maria, Centro Hospitalar Universitário Lisboa Norte, Lisboa, Portugal;* ^2^*Serviço de Genética Médica, Hospital Pediátrico, Centro Hospitalar Universitário de Coimbra, Coimbra, Portugal;* ^3^*Unidade de Genética Médica, Centro de Genética Médica Doutor Jacinto Magalhães, Centro Hospitalar Universitário do Porto, Porto, Portugal;* ^4^*Serviço de Genética Médica, Hospital Dona Estefânia, Centro Hospitalar Universitário Lisboa Central, Lisboa, Portugal;* ^5^*Centro de Genética Preditiva e Preventiva, Instituto de Biologia Molecular e Celular, i3S, Porto, Portugal.*

**Introduction:** the characterization of disease cohorts from different populations is extremely important in genetic conditions. However, this is extremely difficult in rare conditions as the cases are not often centralized in a specific center or diagnostic lab. One of these rare conditions is Fanconi anemia (FA), a chromosome instability syndrome with a multisystemic clinical presentation. In Portugal, the diagnosis of FA is mostly centralized in a laboratory that tests for diepoxybutane (DEB)-induced chromosome breakage. We aimed to assess genotypic-phenotypic (G-P) aspects of the FA Portuguese population.

**Methodology:** we retrospectively reviewed clinical and molecular information from FA cases from January 1991 to June 2023, from the Cytogenetics Laboratory of the School of Medicine and Biomedical Sciences. Cases were considered positive if DEB-treated cell cultures showed at least a mean number of 0.96 chromosome breaks per cell, including the presence of multiple radial figures.

**Results:** A total of 84 patients were diagnosed with FA at a median age of 6 years, out of which 48 (57%) are female. There was positive family history in 14% and consanguinity in 35% of cases. In 37 (44%) cases there was a molecular diagnosis, with 33 of those (89%) presenting biallelic FANCA pathogenic variants. The most common variant was the nonsense FANCA c.295C>T, corresponding to an overrepresentation of the Romani ethnicity, which comprises 31% of cases. Some patients were diagnosed due to positive family history, and were clinically asymptomatic at diagnosis; hematological and morphological features were found in 75% and 58% of cases respectively. Interestingly, patients displaying anemia had a significantly increased number of DEB-induced breaks per cell. Carriers of a homozygous FANCA c.295C>T variant had increased rates of red blood cell (RBC) regeneration, with no other relevant G-P correlation. Twenty-three patients underwent hematopoietic stem cell transplant. In at least 2 patients, somatic genetic rescue was presumed due to long-term loss of the cytogenetic phenotype, through a direct reversal mechanism in one case, and indirect in the other.

**Discussion:** this work illustrates the well-known lack of G-P correlations in FA. Nevertheless, RBC levels seem to be a reliable proxy for cytogenetic and molecular findings, something which has not been prior reported. Another differing aspect from this cohort is that the well-established male overrepresentation is not present. The FANCA c.295C>T variant can be considered overrepresented in the Portuguese cohort. In conclusion, this work highlighted some specificities of the Portuguese FA population as well as the importance of centralizing data on rare diseases.

#### P35 - COMBINED GERMLINE AND TUMOR MUTATION SIGNATURE TESTING IDENTIFIES NEW FAMILIES WITH NTHL1 TUMOR SYNDROME

Carla Pinto^1,2,3,†^, Joana Guerra^2,4,†^, Manuela Pinheiro^2^, Carla Escudeiro^1,2^, Catarina Santos^1,2^, Pedro Pinto^2^, Miguel Porto^2^, Carla Bartosch^5,6^, João Silva^2,7^, Ana Peixoto^1,2^, Manuel R. Teixeira^1,2,8^

^1^ *Department of Laboratory Genetics, Portuguese Oncology Institute of Porto (IPO-Porto)/Porto Comprehensive Cancer Center, Porto, Portugal;* ^2^ *Cancer Genetics Group, IPO-Porto Research Center (CI-IPOP)/RISE@CI-IPOP (Health Research Network), Portuguese Oncology Institute of Porto (IPO-Porto)/Porto Comprehensive Cancer Center, Porto, Portugal;* ^3^ *Department of Pathological, Cytological and Thanatological Anatomy, School of Health, Polytechnic Institute of Porto, Porto, Portugal;* ^4^ *Doctoral Programme in Biomedical Sciences, School of Medicine and Biomedical Sciences (ICBAS), University of Porto, Porto, Portugal;* ^5^ *Department of Pathology, Portuguese Oncology Institute of Porto (IPO-Porto)/Porto Comprehensive Cancer Center, Porto, Portugal;* ^6^ *Cancer Biology and Epigenetics Group, IPO-Porto Research Center (CI-IPOP)/RISE@CI-IPOP (Health Research Network), Portuguese Oncology Institute of Porto (IPO-Porto)/Porto Comprehensive Cancer Center, Porto, Portugal;* ^7^ *Department of Medical Genetics, Portuguese Oncology Institute of Porto (IPO-Porto)/Porto Comprehensive Cancer Center, Porto, Portugal;* ^8^ *School of Medicine and Biomedical Sciences (ICBAS), University of Porto, Porto, Portugal;* ^†^ *These authors have contributed equally to this work and share first authorship.*

**Introduction:** NTHL1 tumor syndrome is an autosomal recessive rare disease caused by biallelic inactivating variants in the NTHL1 gene and which presents a broad tumor spectrum. We aimed to contribute to the characterization of the phenotype of this syndrome.

**Methodology:** We studied 467 index patients by KASP assay or next-generation sequencing, including 228 patients with colorectal polyposis and 239 patients with familial/personal history of multiple tumors (excluding multiple breast/ovarian/polyposis).

**Results/Discussion:** Three NTHL1 tumor syndrome families were identified in the group of patients with polyposis and none in patients with familial/personal history of multiple tumors. Altogether, we identified nine affected patients with polyposis (two of them diagnosed after initiating colorectal cancer surveillance) with biallelic pathogenic or likely pathogenic NTHL1 variants, as well as two index patients with one pathogenic or likely pathogenic NTHL1 variant in concomitance with a missense variant of uncertain significance. Here we identified a novel inframe deletion classified as likely pathogenic using the ACMG criteria, supported also by tumor mutational signature analysis. Our findings indicate that the NTHL1 tumor syndrome is a multi-tumor syndrome strongly associated with polyposis and not with multiple tumors without polyposis.

**Acknowledgments:** FCT PhD fellowship (ref. SFRH/BD/138670/2018); P.CCC NORTE-01-0145-FEDER-072678 (NORTE 2020).

#### P36 - NEURODEVELOPMENT TRENDS IN PORTUGUESE CHILDREN DIAGNOSED IN THE NEWBORN SCREENING WITH PHENYLKETONURIA OR CONGENITAL HYPOTHYROIDISM: A RETROSPECTIVE COHORT STUDY

Celia Azevedo Soares^1,2,3,4^, Carla Carmona^1,2,5^

^1^*Centro de Genética Médica Dr. Jacinto Magalhães, Centro Hospitalar Universitário do Porto, Porto, Portugal.* ^2^ *Unidade Multidisciplinar de Investigação Biomédica, Instituto de Ciências Biomédicas Abel Salazar (UMIB/ICBAS) and Laboratory for Integrative and Translational Research in Population Health (ITR), Universidade do Porto, Porto, Portugal.* ^3^ *Departamento de Ciências Médicas, Universidade de Aveiro, Aveiro, Portugal.* ^4^ *i3S - Instituto de Investigação e Inovação em Saúde, Universidade do Porto, Porto, Portugal.* ^5^ *Centro de Referência de Doenças Hereditárias do Metabolismo, Centro Hospitalar Universitário do Porto, Porto, Portugal.*

**Introduction:** Neurodevelopmental disorders pose a significant public health concern, with early diagnosis and intervention being crucial for optimal outcomes. This study investigates the neurodevelopmental trends of children diagnosed with phenylketonuria (PKU) or congenital hypothyroidism (CHT) in the newborn screening, aiming to shed light on the timing of neurodevelopmental challenges in a Portuguese cohort.

**Methodology:** In this retrospective cohort study we analyzed a comprehensive dataset of 98 patients with PKU and 168 with CHT, including neurodevelopmental and intellectual quotient (IQ) assessment. The primary outcome measure was IQ in childhood.

**Results:** Our findings revealed distinct neurodevelopmental trends in the two groups. Children with CHT who later a lower IQ have exhibited lower neurodevelopmental assessment scores in their first year of life, highlighting the early onset of cognitive challenges in this population. In contrast, children with PKU with a lower IQ later in childhood displayed a decline in neurodevelopmental scores only after the third year of life, suggesting a delayed onset of neurocognitive difficulties compared to those with congenital hypothyroidism.

**Discussion:** This study underscores the importance of early and continuous monitoring of neurodevelopment in children diagnosed with PKU and CHT. Early diagnosis and targeted interventions for children with congenital hypothyroidism may help mitigate cognitive deficits, while close monitoring beyond the third year of life is essential for those with PKU. Further studies are needed to clarify the differences in the age of onset of neurodevelopment difficulties in these two populations.

#### P37 - DELIBERATING GENETIC RISKS: PROTOCOL OF A PROSPECTIVE, MULTI-METHOD STUDY ON PATIENT DECISION-MAKING AND DISCLOSURE FROM GENETIC COUNSELLING TO THE FAMILY

Milena Paneque^1,2^, Maria Barbosa^1,3^, Angus Clarke^4^, Sofia Fontoura Dias^5^, Filipa Júlio^6,7,8^, Alison Metcalfe^9^, Jorge Sequeiros^1,2^, Liliana Sousa^4^, Álvaro Mendes^1^

^1^ *UnIGENe and CGPP – Centro de Genética Preditiva e Preventiva, IBMC – Institute for Molecular and Cell Biology, i3S – Instituto de Investigação e Inovação em Saúde, University of Porto, Portugal;* ^2^ *ICBAS - School of Medicine and Biomedical Sciences, University of Porto, Portugal;* ^3^ *Faculty of Psychology and Educational Sciences, University of Porto, Portugal;* ^4^ *Division of Cancer & Genetics, Institute of Medical Genetics, Cardiff University School of Medicine, UK;* ^5^ *Department of Education and Psychology, CINTESIS@RISE, University of Aveiro, Portugal;* ^6^ *European Huntington Association and Portuguese Huntington Association;* ^7^ *Faculty of Psychology and Educational Sciences, University of Coimbra, Portugal;* ^8^ *CIBIT – Coimbra Institute for Biomedical Imaging and Translational Research, University of Coimbra, Portugal;* ^9^ *LOHA Health Ltd, UK*

The confirmation of a genetic disease in a patient has implications for family members, who may be at risk of developing the condition or of passing it on to their offspring. Family communication of risk information is commonly encouraged in genetic counselling (GC), but this communication may be challenging and, often, some at-risk relatives remain uninformed. Barriers and facilitators to communicating genetic risk within the family are known; yet, evidence is limited on how those impact on the patient’s decision-making process, and how GC influences these decisions.

We describe a protocol, for the project DECIDE, which aims to uncover the course of patients’ decision-making about disclosure of genetic information to family members and to co-produce a counselling framework that assists healthcare providers in facilitating family communication of genetic risks.

The specific goal of DECIDE is to investigate the patient’s journey throughout the decision-making process by examining the impact of GC on how patients deliberate their disclosure decisions, and the role of the family and other lifeworld factors in that process. This project draws on a prospective, sequential, multi-methods study. First, we will observe genetic counselling clinics to investigate the framings and discussions when addressing communication with the family. Secondly, patients will be invited to keep diaries, through which we will examine their process of reflection and how it translates into communication with the family. Finally, in-depth interviews with patients will be conducted to explore their overarching and more settled perspectives around family disclosure. Data will be collected in medical genetics departments across Portugal.

Data will be analysed using a composite of theme-oriented discourse analysis of observations of genetic counselling clinics, and reflexive thematic analysis of diaries and interviews. We will focus on connecting the negotiation of meaning in relation to genetic counselling and patient decision-making. This will provide an empirical foundation upon which a multi-stakeholder panel will engage (involving representatives of patient advocacy groups, healthcare professionals and researchers) to co-produce and validate a consensus-based counselling framework, using a Delphi study with specific values, strategies, attitudes and practical points to supplement GC.

We hope this project may contribute to fill in knowledge gaps on GC research and support patients regarding family communication of risk information.

This project has received funding from the FCT - Fundação para a Ciência e Tecnologia (2022. 04025.PTDC).

#### P38 - GENETIC MODIFIERS OF SICKLE CELL ANEMIA SEVERITY, IN AN ANGOLAN COHORT

Catarina Ginete^1^, Mariana Delgadinho^1^, Brígida Santos^2,3^, Armandina Miranda^4^, Carina Silva^1,6^, Paulo Guerreiro^1^, Emile R. Chimusa^5^, Miguel Brito^1,2^

^1^ *H&TRC- Health & Technology Research Center, ESTeSL- Escola Superior de Tecnologia da Saúde, Instituto Politécnico de Lisboa, Lisboa, Portugal.* ^2^ *Centro de Investigação em Saúde de Angola (CISA), Bengo, Angola;* ^3^ *Hospital Pediátrico David Bernardino (HPDB), 3067 Luanda, Angola* ^4^ *Instituto Nacional de Saúde Doutor Ricardo Jorge (INSA), Lisboa, Portugal;* ^5^ *Department of Applied Sciences, Faculty of Health and Life Sciences, Northumbria University, Newcastle, Upon Tyne, NE1 8ST, UK.* ^6^ *Centro de Estatística e Aplicações, Universidade de Lisboa, Lisboa, Portugal*

**Introduction:** Sickle Cell Anemia (SCA) is an inherited disease caused by a single nucleotide substitution in HBB gene, that encodes for the B-globin subunit of hemoglobin. Although patients’ phenotypes are very heterogenous, in terms of severity and life spam, patients homozygous for this mutation usually exhibit chronic hemolytic anemia, report frequent and severe painful crisis and present extensive organ damage. The aim of this study was to identify genetic modifiers of SCA phenotypes and severity in the HBB Cluster, HBS1L-MYB intergenic region, BCL11A, KLF1, FOX3, and ZBTB7A genes, and assess their influence and prevalence in an Angolan population.

**Methodology:** Using next-generation sequencing, HBB Cluster, HBS1L-MYB intergenic region, BCL11A, KLF1, FOX3, and ZBTB7A genes, from samples of 192 SCA children, were sequenced. Patient’s severity phenotypes were evaluated considering hematological, biochemical, and clinical data.

**Results:** Samples from 192 Angolan patients (99 female), aged between 3 and 12 years old, were sequenced and 5,019,378 variants of high quality were registered. Values of Fetal hemoglobin ranged from 0,7 and 23.8%, and children were grouped according to previous manifestations/phenotype (Hemolytic, vaso-occlusive and less severe phenotypes).

**Discussion:** Two SNPs in the intronic region of chromosome 2q16.1, harboring the BCL11A gene, rs1427407 (P=1.29e−09, MAF=0.22) and rs71327644 (P=7.39e-08, MAF=0.30) are genome-wide significant associated with decreasing HbF. Several variants, identified in 18 and 12 chromosomal regions, were also identified as nominally associated with the decrease of HbF and with VOC phenotype, respectively. Most of the genes associated with these variants are part of BCL11A functional/physical and co-expression network. To our knowledge, this is the first investigation of clinical variation in SCA in Angola using a well-customized and targeted sequencing approach, bringing significant contributions to present knowledge of the clinical heterogeneity of SCA.

This research was funded by FCT/Aga Khan (project nº330842553) and FCT/MCTES (UIDB/05608/2020 and UIDP/05608/2020) –H&TRC.

The authors declare that they have no competing interests.

#### P39 - COMMUNICATING GENETIC INFORMATION IN FAMILIES WITH LATE-ONSET NEUROLOGICAL DISEASES: A SCOPING REVIEW

Sofia Fontoura Dias^1,2^, Maria Barbosa^3,4^, Filipa Júlio^5,6,7^, Milena Paneque^3,8^, Jorge Sequeiros^3,8^, Liliana Sousa^1,2^, Álvaro Mendes^3^

^1^ *Department of Education and Psychology, University of Aveiro, Aveiro, Portugal;* ^2^ *CINTESIS@RISE - Center for Health Technology and Services Research, University of Aveiro, Aveiro, Portugal;* ^3^ *UnIGENe and CGPP – Centro de Genética Preditiva e Preventiva, IBMC – Institute for Molecular and Cell Biology, i3S – Instituto de Investigação e Inovação em Saúde, University of Porto, Porto, Portugal;* ^4^ *Faculty of Psychology and Educational Sciences, University of Porto, Porto, Portugal;* ^5^ *European Huntington Association and Portuguese Huntington Association;* ^6^ *Faculty of Psychology and Educational Sciences, University of Coimbra, Coimbra, Portugal;* ^7^ *CIBIT – Coimbra Institute for Biomedical Imaging and Translational Research, University of Coimbra, Coimbra, Portugal;* ^8^ *ICBAS School of Medicine and Biomedical Sciences, University of Porto, Porto, Portugal.*

Communicating genetic information in families with late-onset neurological diseases (LONDs) is complex due to the limited medical actionability of genetic risk information. Despite the abundant literature on communication of genetic information in families with preventable or treatable conditions, no reviews have specifically addressed LONDs. This scoping review aims to map the research evidence on the communication of genetic information in families with LONDs, to identify its key characteristics and the obstacles and facilitators involved.

This review followed the JBI and PRISMA guidance. It included peer-reviewed primary research articles, published since 1997, and written in English, Portuguese, Spanish, or French. Studies concerning individuals at risk or with LONDs or their relatives and focused on family communication of genetic information were included.

In total, 30 articles met the eligibility criteria. These were published from 1997 to 2023, mainly conducted in the USA (n=9). Most focused on families with Huntington’s disease (n=26) and used qualitative research methods (n=26). A preliminary analysis suggested that most studies addressed whether and why, what, how, and when to communicate, and who was involved. Studies emphasized an individual’s responsibility to inform relatives (whether and why). Parents, particularly older generations, were seen as pivotal figures, mainly women (who). The communication content included selected information on symptoms, prognosis, or the course of the condition (what), which could be shared either abruptly or gradually (how). Finding the “right time” to disclose, especially to children or partners (when), was often reported. Ultimately, decisions about communication were influenced by prior knowledge of the condition, family dynamics, as well as the perceived vulnerability and receptivity of family members. Stigma was often cited as a barrier to family communication, leading to secrecy.

Future research should examine family communication about LONDs using longitudinal approaches. Also, how the prospect of available effective therapies may influence family intentions and communication patterns should be investigated.

#### P40 - GENETIC TESTING FOR GERMLINE VARIANTS IN HOMOLOGOUS RECOMBINATION REPAIR GENES, OTHER THAN BRCA1 AND BRCA2, IN PATIENTS WITH SUSPECTED HEREDITARY CANCER SYNDROMES

Daniela Arnaut^1^, Pedro Rodrigues^1,2^, Patrícia Theisen^1^, Dina Carpinteiro^1,2^, Luís Vieira^1,2^, João Gonçalves^1,2^

^1^*Human Genetics Department, National Institute of Health Dr. Ricardo Jorge, Lisbon, Portugal.* ^2^ *Center for Toxicogenomics and Human Health, Nova Medical School, Lisbon, Portugal. Support: FCT/MCTES, Projects - ToxOmics and Human Health (UIDB/00009/2020) and GenomePT (POCI-01-0145-FEDER-022184).*

Homologous recombination repair (HRR) is the cellular mechanism for error-free repair of DNA double-strand breaks. Pathogenic germline variants in BRCA1 and BRCA2 lead to HRR deficiency associated with breast, ovarian, prostate, pancreatic cancers and are sensitive to PARP inhibitors (PARPi). Defects in HRR genes beyond BRCA1/2 could also result in HRR deficiency and sensitize the tumor to PARPi, thus expanding the subset of patients that can benefit from these targeted therapy cancer drugs.

We studied 56 DNA samples obtained from patients with personal and family history of cancer. Genes involved in HRR (ATM, BAP1, BLM, BRIP1, FANCA, FANCB, FANCC, FANCD2, FANCE, FANCF, FANCG, FANCI, FANCL, FANCM, NBN, PALB2, RAD51C, RAD51D) were analysed by NGS using TruSight® Hereditary Cancer. Sequence alignment and annotation included DRAGEN Enrichment and Variant Interpreter - Illumina®. Variant classification, according to ACMG-AMP 1, was based on VEP, HSF, Alamut, VarSome and several databases (ex. HGMD, gnomAD, dbSNP). Variants of uncertain significance (VUS) were also classified with the stepwise ABC system2. All pathogenic/likely pathogenic SNVs and CNVs were confirmed by Sanger sequencing or MLPA.

We identified 156 SNVs and one CNV, of these 125 were benign/likely benign. Seven clinically actionable variants were found in 10.7% of the patients: 3 pathogenic variants in FANCA, FANCD2 and FANCI give rise to premature stop codons and one pathogenic CNV in FANCA (deletion of exons 38 and 39); 2 likely pathogenic variants in BLM and FANCI affecting splicing and one frameshift in FANCG. Classification of 18 VUS with the ABC system resulted in: 8 class 0 (normal finding), 7 class E (potential interest) and 3 class D (low penetrance) variants. In addition, 7 SNVs were classified as hypomorphic alleles.

This study confirmed: i) the importance of extending the molecular study beyond BRCA1/2 to other genes involved in HRR, ii) some variants require functional/family studies to establish their pathogenicity, and iii) these genes could potentially be considered for specific and clinical studies involving PARPi therapy.

1 - doi:10.1038/gim.2015.30; 2 - doi:10.1038/s41431-021-00903-z.

#### P41 - GENETIC STUDY OF PATIENTS WITH PRIMARY CILIARY DYSKINESIA AND INFERTILITY

Nygell Alves^1,2^, Telma Barbosa^2,3^, Catarina Dias^2,4^, Edite Ferreira^2,5^, Ângela Alves^1,2^, Jorge Oliveira^2,6^, Mário Sousa^1,2,*^, Rute Pereira^1,2,*^

^1^*1Laboratory of Cell Biology, Department of Microscopy, ICBAS-School of Medicine and Biomedical Sciences, University of Porto, Porto, Portugal;* ^2^*UMIB-Unit for Multidisciplinary Research in Biomedicine, ICBAS-UP/ ITR-Laboratory for Integrative and Translational Research in Population Health, University of Porto, Porto, Portugal;* ^3^*Department of Children and Adolescents, Centro Materno-Infantil do Norte (CMIN), Centro Hospitalar Universitário de Santo António (CHUdSA), Porto, Portugal;* ^4^*Department of Pneumology, Centro Hospitalar Universitário de Santo António (CHUdSA), Porto, Portugal;* ^5^ *Department of Otorhinolaryngology, Centro Hospitalar de Vila Nova de Gaia/Espinho (CHVNG/E), Vila Nova de Gaia, Portugal;* ^6^ *Center for Predictive and Preventive Genetics, Institute of Health Research and Innovation (IBMC/i3S), University of Porto, Porto, Portugal;* ^*^Equal contributing authors

Primary ciliary dyskinesia (PCD) is a rare autosomal recessive disease caused by defects on motile cilia structure/function. Cilia are conserved and complex cell structures with vital biological functions. Clinical manifestations vary, encompassing respiratory issues, infertility, and laterality disorders. PCD diagnosis is challenging and depends on a multidisciplinary team and several laboratory techniques, including the analysis of ciliary function by high-speed video-microscopy (HSVM), ciliary structure by transmission electron microscopy (TEM), and genetic analysis. Currently, 45 genes are linked to PCD, but the genetic diagnosis is still complex, as about 30% of the patients remain without a conclusive diagnosis.

We here investigate the genotype-phenotype correlations in three female patients with a clinical suspicion of PCD and infertility.

Under written informed consent, blood and nasal brushing samples were obtained. The ciliary beat frequency (CBF) and pattern (CBP) were assessed by HSVM, and the axoneme ultrastructure was evaluated by TEM. Genomic DNA was extracted from peripheral blood for whole exome sequencing (WES) analysis.

HSVM analysis revealed a low CBF mean (< 5 Hz), compared to the reference CBF (mean 12.75 Hz) in all three cases; regarding CBP, two patients presented a 100% dyskinetic pattern, and one a 91.7% dyskinetic pattern. TEM analysis showed defects in axonemal dynein arms (motor structures). WES evaluation identified 4 heterozygous compound variants (2 in DNAH5 and 2 in CCDC40) in one patient, a homozygous splicing variant in CCDC39 in another patient, and a homozygous splicing variant in CCDC40 in the third patient. These variants are extremely rare (allelic frequencies < 0.01% in the global population) and considered pathogenic by bioinformatic tools. To confirm pathogenicity, we are assessing the variant impact through mRNA and protein expression analyses in patient and family samples.

We foresee that these results will enhance genotype-phenotype knowledge in PCD, aiding in pathophysiology comprehension, and improving the genetic counselling.

#### P42 - WHAT PROCESS STUDIES IN GENETIC COUNSELING CAN TEACH US ABOUT COMMUNICATION OF GENETIC INFORMATION TO FAMILY MEMBERS: A SCOPING REVIEW

Maria Barbosa^1,2^, Sofia Fontoura Dias^3,4^, Filipa Júlio^5,6,7^, Milena Paneque^1,8^, Célia Sales^2,9^, Jorge Sequeiros^1,8^, Liliana Sousa^3,4^, Álvaro Mendes^1^

^1^*UnIGENe and CGPP – Centro de Genética Preditiva e Preventiva, IBMC – Institute for Molecular and Cell Biology, i3S – Instituto de Investigação e Inovação em Saúde, University of Porto, Porto, Portugal;* ^2^ *Faculty of Psychology and Educational Sciences, University of Porto, Porto, Portugal;* ^3^ *Department of Education and Psychology, University of Aveiro, Aveiro, Portugal;* ^4^ *CINTESIS@RISE - Center for Health Technology and Services Research, University of Aveiro, Portugal;* ^5^ *European Huntington Association and Portuguese Huntington Association;* ^6^*Faculty of Psychology and Educational Sciences, University of Coimbra, Coimbra, Portugal;* ^7^ *CIBIT – Coimbra Institute for Biomedical Imaging and Translational Research, University of Coimbra, Coimbra, Portugal;* ^8^ *ICBAS School of Medicine and Biomedical Sciences, University of Porto, Porto, Portugal;* ^9^ *CPUP - Center for Psychology at the University of Porto, Porto, Portugal.*

Few studies examine how healthcare professionals (HCPs) address family communication in genetic counselling (GC). Process studies in GC may elucidate less-known aspects of practice, but no literature reviews have exclusively focused on process studies that explore how communicating genetic risk information to family members is addressed in GC. This scoping review aims to fill this gap by investigating roles and practices of HCPs concerning family disclosure during GC.

This scoping review followed the JBI and PRISMA guidance. It included peer-reviewed primary research articles published from 1997 onwards and written in English, Portuguese, Spanish, or French. Studies that reported how family communication of genetic information is addressed in GC, either from the perspective of HCPs or the patients, were included. Four databases (Web of Science, PubMed, PsycINFO and Scopus) were searched and 21 articles met the eligibility criteria for analysis. These were published between 2005 and 2022; seven were conducted in Australia. Nearly all (n=19) studies were retrospective and qualitative, using interviews, focus groups, clinical notes and audio-recordings of GC appointments. Eight studies focused on HCPs’ views, 9 on patients’ views, and 4 on both, one being an observational study.

Preliminary analysis suggested that HCPs consistently mentioned discussing family disclosure with patients. Some studies, however, reported that family disclosure was discussed only minimally during GC or, sometimes, not at all. HCPs’ roles and practice included providing information, using written materials, guidance and multidisciplinary support, conducting psychosocial assessments, and addressing practical procedures. Patients frequently suggested how HCPs could facilitate family disclosure, with most suggestions reflecting practices already adopted by HCPs.

This review provides insight that may inform ongoing discussions on the scope of practice, roles, and responsibilities of HCPs in supporting patients in sharing information with family members. Further prospective studies are needed to clarify how GC influences decisions to disclose genetic risk information and family communication.

#### P43 - A NOVEL DE NOVO SERPINC1 VARIANT ASSOCIATED WITH ANTITROMBIN III DEFICIENCY

Rita Certã^1^, Isabel Moreira^1^, Sara Morais^2^, João Gonçalves^1,3^

^1^ *Human Genetics Department, National Institute of Health Dr. Ricardo Jorge, Lisbon, Portugal.* ^2^ *Clinical Haematology Service, Hospital Geral Santo António, Centro Hospitalar Universitário de Santo António, Porto, Portugal.* ^3^*Center for Toxicogenomics and Human Health, Nova Medical School, Lisbon, Portugal.*

**Introduction:** Antithrombin III (ATIII), encoded by SERPINC1 gene (with 7 exons lies in 1q25.1), is a natural anticoagulant glycoprotein that inhibits thrombin and activated factors X and XI regulating the blood coagulation cascade. ATIII deficiency is associated with increasing risk for venous thrombosis, is present in 1-2% of thrombosis cases and can be inherited or acquired. Inherited deficiencies are divided into type I and type II. In type I, the concentration and activity of ATIII are decreased while in type II, despite its normal concentration, the functional activity is low. ATIII deficiency usually has an autosomal dominant pattern of inheritance.

**Methods:** A 40 years old female patient clinically diagnosed with ATIII deficiency (Activity: 41%; Antigenic: 54%; ref.>80%) and no family history of thrombosis, was sent for molecular studies. Genomic DNA was used for SERPINC1 analysis (PCR and Sanger-sequencing). Bioinformatic studies were performed using several pathogenicity prediction tools (Alamut Visual; VarSome; Human Splicing Finder; Franklin by genoox).

**Results and Discussion:** SERPINC1 analysis allowed the identification of the missense variant: c.512A>G, p.(Lys171Arg), present in heterozygosity. This alteration is not reported in any database, neither in scientific literature. Physicochemical differences between Lys and Arg are small, and Arg is weakly conserved between species. in silico analysis to assess the variant´s consequences suggested that its protein function is not affected (PolyPhen2: benign; SIFT: tolerated; MutationTaster: polymorphism). Splicing predictors suggested that normal mRNA processing may be affected, due to a new donor splice site. According to ACMG-guidelines, this alteration can be classified as variant of uncertain significance (VUS) (PM2_moderate, PM1_supporting). Family studies confirming that it was a de novo event (absent in both parents). As no other variant was identified in this gene, c.512A>G, p.(Lys171Arg) is most likely associated with the ATIII deficiency in this patient. Further investigation, including mRNA studies, are planned in order to characterise the molecular mechanism underlying the ATIII deficiency.

#### P44 - NEUROFIBROMATOSIS TYPE I: MOLECULAR DIAGNOSIS IN A PORTUGUESE COHORT

Carolina Teixeira^1,2^, Mário Laço^3^, Patrícia Martinho^2^, Janet Pereira^2,4^, Fabiana Ramos^5^, Sofia Fernandes^6^, Teresa Fidalgo^2^

^1^ *1Faculty of Medicine, University of Coimbra, Coimbra, Portugal;* ^2^*Molecular Hematology Functional Unit, Centro Hospitalar e Universitário de Coimbra, Coimbra, Portugal;* ^3^*Medical Genetics Unit, Centro Hospitalar do Tâmega e Sousa, Penafiel, Portugal;* ^4^*Research Centre for Anthropology and Health (CIAS), Department of Life Sciences, University of Coimbra, Coimbra, Portugal;* ^5^*Medical Genetics Unit, Hospital Pediátrico, Centro Hospitalar e Universitário de Coimbra, Coimbra, Portugal;* ^6^*Family Risk Clinic, Portuguese Oncology Institute of Lisbon (IPO Lisbon), Lisboa, Portugal.*

**Introduction:** Neurofibromatosis type I (NF1), an autosomal dominant disorder, is one of the most common RASopathies with an incidence of 1 in 2500 to 1 in 3000 individuals. It’s mainly characterized by multiple café-au-lait macules, cutaneous and subcutaneous neurofibromas, iris hamartomas, freckling of axillary and inguinal regions and plexiform neurofibromas. NF1 is a disease caused by loss-of-function variants in a tumor suppressor gene, the neurofibromin 1 gene (NF1). More than 4000 pathogenic variants are reported and around 30% of the pathogenic NF1 DNA variants affect the splicing event. The aim of this work was to carry out the molecular diagnosis of patients with clinical suspicion of NF1 using a two-step strategy genomic DNA (gDNA) and complementary DNA (cDNA).

**Methodology:** In this study, 34 individuals from 28 unrelated families with NF1 clinical suspicion were studied. The sequential study of NF1 starts with the study of the coding region by next generation sequencing (NGS), proceeds to the study of copy number variations (CNV) by multiplex ligand probe amplification (MLPA) and ends with cDNA analysis.

**Results:** Nineteen different variants have been identified in NF1, in 20 of 28 probands, of which two are de novo and four are novel (c.5692dup, c.2614G>T, c.1392 + 751T>G, c.(3974 + 10_3982)_(6250_6387)del). Seventy-three percent of patients were NF1 positive and 27% negative. The NGS study revealed 16 different of variants, MLPA identified a large deletion, and cDNA Sanger sequencing allowed the identification of a deep intronic variant and the confirmation of the previously identified splice variant (skipping of exon 31 was detected). The cDNA study was also performed on novel variants to understand their effect on mRNA. Six variants were classified according to the NF1 splicing variant system classification1, based on their effect on the splicing event.

**Discussion:** This two-step strategy proved to be highly efficient, as the combination of three different and complementary methods (gDNA and cDNA sequencing, and MLPA) it was able to identify 19 different variants, four of which had not been previously described. The addition of cDNA sequencing to the diagnostic algorithm was fundamental to understanding the impact of DNA variants on NF1 mRNA. Phenotype-genotype correlation in NF1 is usually difficult and it was no exception in this study, although some correlations could be established. To better assess the sensitivity of this two-step strategy, a large number of individuals should be studied.

[1] Wimmer K, et al (2007) Hum Mut 28: 599.

#### P45 - GENETIC TESTING FOR CARDIOMYOPATHY IN AN UNIVERSITY HOSPITAL IN BRAZIL

Maria Angelica de Faria Domingues de Lima^1^, Antonio Pedro Lima Costa Pereira^2^, Matheus Medeiros Foureaux^2^, Ana Gabriela Medeiros^2^, Lucas Fonseca da Silva^2^, Fabio de Souza^2^

^1^*Gaffrée and Guinle University Hospital – HUGG/EBSERH;* ^2^ *Federal University of the State of Rio de Janeiro - UNIRIO*

Hereditary cardiomyopathy is a heterogenous group of diseases that affect families and has a major risk of arrhythmia, cardiac failure and sudden death. The uptake of genetic testing is small in Brazil, especially in the public health system. We report on the experience of genetic testing for hereditary cardiomyopathy among patients of a university hospital in Rio de Janeiro.

All patients with primary cardiomyopathy, who underwent genetic testing for primary cardiomyopathy in the cardiology or genetics out-patient clinic at Gaffrée and Guinle Hospital were invited to participate in this study. This is an observational study. Data about cardiomyopathy phenotype, age at diagnosis, genetic testing results, and family history was collected from all participants.

From 2021 until 2023, 21 patients were included in the study; 71% were male and the mean age was 57 years old. 62% presented hypertrophic phenotype and 38% dilated cardiomyopathy. Other phenotypes were not observed in this sample. Family history was positive for sudden death, cardiomyopathy, arrhythmia, or myocardial infarction in 66%. Genetic testing identified pathogenic variants in 8 cases and variants of unknown significance in another 9 cases. Most were index cases for families, except for two patients who were cousins. Pathogenic variants were identified in BAG3 (1), TTR (3), MYH7 (2) and GLA (2). Noteworthy, one case of dilated phenotype was associated with TTR variant, however, further investigation excluded amyloid cardiomyopathy.

Even though the interest in hereditary cardiomyopathy has increased in past years, the uptake of genetic testing is still discrete. Studies show that most cases of cardiomyopathy are associated to MYH7, TTN and MYBPC3 genes. Even though this sample has showed different results, we feel it is still too small to draw conclusions. Access to genetic testing is a problem in Brazil, as it is expensive, private health insurance does not cover and is unavailable in the public health system; therefore, sponsored testing or testing in the research setting are the available options for most of the population.

Declaration of Interests: No conflict of interests

#### P46 - PHENOTYPIC AND GENETIC ANALYSIS OF MACROTHROMBOCYTOPENIA PATIENTS WITH VARIANTS IN GLYCOPROTEIN IB ALPHA SUBUNIT (GP1BA), GLYCOPROTEIN IB BETA SUBUNIT (GP1BB) AND GLYCOPROTEIN IX (GP9)

Catarina Monteiro^1,2,3,4^, Ana Gonçalves^1,3,4^, Mónica Pereira^2,3,4^, Marta Gonçalves^3,4,5^, Catarina Lau^3,4,5^, Eugénia Cruz^2^, Sara Morais^2,3,4^, Rosário Santos^1,3,4^

^1^ *Laboratório de Genética Molecular, Serviço de Genética Laboratorial, Centro de Genética Médica Jacinto Magalhães, Centro Hospitalar Universitário de Santo António (CHUdSA), Porto.* ^2^ *Unidade de Trombose e Hemostase & Centro de Coagulopatias Congénitas, Serviço de Imunohemoterapia, Centro Hospitalar Universitário de Santo António (CHUdSA), Porto.* ^3^ *UMIB - Unit for Multidisciplinary Research in Biomedicine, ICBAS - School of Medicine and Biomedical Sciences, University of Porto (UMIB/ICBAS/UP).* ^4^ *ITR - Laboratory for Integrative and Translational Research in Population Health, University of Porto, Porto.* ^5^ *Unidade de Diagnóstico Hematológico, Serviço de Imunohemoterapia, Centro Hospitalar Universitário de Santo António (CHUdSA), Porto.*

**Introduction:** The GPIb/IX/V complex is the platelet receptor for von Willebrand factor (VWF), essential for haemostasis. Homozygous or compound heterozygous variants in GP1BA, GP1BB or GP9, encoding the complex subunits, are at the origin of Bernard-Soulier syndrome (BSS), a bleeding disorder characterised by macrothrombocytopenia and platelet dysfunction, due to reduced or dysfunctional complex. Recently reported monoallelic cases, with GP1BA or GP1BB variants transmitted as an autosomal dominant state, show moderate phenotypes. Gain-of-function (GoF) GP1BA variants are associated with an increase in the binding affinity for VWF, resulting in platelet-type von Willebrand Disease (PT-VWD).

**Methodology:** From a cohort of patients with inherited thrombocytopenia, 14 probands were suspected of having GPIb-IX-V involvement, based on clinical data, platelet counts and size, and functional studies (ristocetin-induced platelet agglutination and GPIb/IX glycoproteins expression quantification). Genetic screening was performed by NGS and/or Sanger sequencing. The variant’s pathogenicity was assessed by ACMG recommendations, segregation studies and *in-silico* predictions.

**Results:** Genetic studies allowed the confirmation of 13 BSS families and 1 PT-VWD case. Five families were identified as biallelic BSS, 3 with variants in GP1BA and 2 with a pathogenic GP9 variant. Regarding the eight monoallelic BSS families, 4 presented variants in GP1BB and 4 in GP1BA. Phenotypically, biallelic BSS cases have a more pronounced PLT dysfunction phenotype compared to monoallelic. Furthermore, a new GoF variant in GP1BA was identified in a novel case of PT-VWD. Overall, 10 new variants were found (6 in GP1BA and 4 in GP1BB) and classified as variants of unknown significance (5), Likely Pathogenic (4) or Pathogenic (1).

**Discussion:** This work expands the spectrum of heterozygous variants in GP1BA and GP1BB associated with monoallelic BSS. Upon clinical and genetic evaluation, a diagnosis was obtained in a total of 27 cases, from 14 unrelated families. In general, BSS variants were identified in the extracellular and transmembrane regions, involved in GPIb-IX-V assembly and ligand binding.

#### P47 - GENOTYPE-PHENOTYPE ASSOCIATIONS OF HERITABLE TP53-RELATED CANCERS BEARING LOSS-OF-FUNCTION OR DOMINANT NEGATIVE PATHOGENIC VARIANTS

Dias A.^1,2,3^, Quental R.^4^, Sousa S.^2,3^, Barbosa-Matos R.^2,3^, Oliveira C.^2,3^

^1^ *Abel Salazar Biomedical Sciences Institute (ICBAS), Porto;* ^2^ *Institute for Research and Innovation in Health (i3S), Porto;* ^3^ *Institute of Molecular Pathology and Immunology of the University of Porto (IPATIMUP), Porto;* ^4^ *University Hospital Center of São João (CHSJ), Porto.*

**Background/Objectives:** TP53 germline pathogenic variants predispose to diverse cancers. Non-truncating variants are often Dominant-Negative (DNE) and truncating Loss-of-Function (LoF). Intensive surveillance of target organs reduces cancer-related mortality; however, it is difficult to prioritize high-risk organs for surveillance in different TP53 variant types. We conducted a genotype-phenotype analysis considering LoF vs. DNE pathogenic TP53 variant carriers.

**Methods:** We selected ClinVar TP53 pathogenic/likely pathogenic variants reviewed by expert panels/consensual among multiple submitters to guide data collection from IARC TP53 Database. Variants were classified as LoF (frameshift, nonsense, out-of-frame splice-site, large deletions, and missense) or DNE (missense). Genotype-phenotype associations were analyzed using Mann-Whitney or χ2 tests and multivariable logistic regression models.

**Results:** We included 2772 clinical data registries from 2106 patients belonging to 930 families carrying 210 distinct variants. 64% (1764/2772) cancer-phenotypes had DNE variants and 62% (1717/2772) occurred in females. Multivariable logistic regression showed that adrenal gland, esophagus, and prostate carcinomas were associated with LoF variants, while brain and lung carcinomas and other sarcomas with DNE variants (p<0.05). Both DNE and LoF variants have lower age-of-diagnosis in male patients (p=0.003 and p=0.004, respectively). The five core cancers also exhibit a significantly lower age-of-diagnosis than non-core (p<0.0001) cancers, and DNE-associated phenotypes had lower age-of-diagnosis, specially in the non-core cancers (p<0.05).

**Conclusion:** The estimated risks of specific cancers depend on the type or germinative variant (DNE or LoF), support, if further validated, privileged surveillance of specific organs/systems according to the TP53 variant molecular type.

#### P48 - HEREDITARY BREAST OVARIAN CANCER (HBOC): A PROJECT TO OPTIMIZE HEREDITARY CANCER PATHWAYS TO SAVE LIVES AND COSTS

Ana Mamede^1^, Luzia Garrido^1,2,3^, Liliana Sousa^1,4^, Renata Oliveira^2^, João Parente Freixo^2^, Pedro Louro^2^, Susy Costa^3^, André Magalhães^3^, Sónia Sousa^1,4^, Susana Fernandes^5^, Sérgio Castedo^1,2,4^, Carla Oliveira^1,4^

^1^ Instituto de Investigação e Inovação em Saúde (i3S), Universidade do Porto, Porto, Portugal;^2^ Serviço de Genética Médica, Centro Hospitalar Universitário São João, Porto, Portugal;^3^ Centro de Mama, Centro Hospitalar Universitário São João, Porto, Portugal;^4^ Instituto de Patologia e Imunologia Molecular da Universidade do Porto, Porto, Portugal;^5^ Serviço de Genética, Faculdade de Medicina da Universidade do Porto, Porto, Portugal

**Introduction:** Hereditary Breast and Ovarian Cancer (HBOC) syndrome is caused by inactivating BRCA1 and BRCA2 germ-line variants. Female carriers have high-risk of developing one or more deathly early-onset cancers in breast, ovary and pancreas, while males may develop prostate, breast and pancreatic cancer. Intensive surveillance and/or prophylactic surgery decrease cancer risk and increase carrier lifespan, but the economic burden of prevention is unknown. We aim to estimate the economic impact that the application of optimized care pathways and multidisciplinary care management may have in patients and families with HBOC.

**Methodology:** We performed a retrospective analysis of clinical and molecular data from Oncogenetic clinical records of families with germline variants in BRCA1/2 followed at Centro Hospitalar Universitário São João (CHUSJ) that will be used to anticipate patients’ individual trajectories of HBOC patients and prevention cost-effectiveness analysis.

**Results:** We identified 220 families carrying a BRCA1/2 Pathogenic/Likely Pathogenic variant with a total of 1002 individuals tested. BRCA1 variants were identified in 78 families with 332 family members tested (218 positive, 114 negative) and BRCA2 variants in 139 families with 658 individuals tested (354 positive, 297 negative and 7 waiting for the result). Three additional families have both a BRCA1 and a BRCA2 variant, with 12 family members studied (5 positive for both, 3 negative for both, 3 positive for the BRCA1 variant and negative for the BRCA2 variant and 1 negative for BRCA1 and positive for BRCA2.

**Discussion/ Conclusions:** These data will be used to calculate the cost and impact of preventive measures applied to HBOC patients in the north of Portugal.

#### P49 - NOVEL INSIGHTS INTO LIFETIME RISK IN HDGG: LOWER CANCER RISK FOR THE MOST RECURRENT CDH1 VARIANT IN EUROPE

Barbosa-Matos R.^a,b,c^, Fonseca J.^a,b,d^, Garrido L.^e^, Mamede A.^a,e^, Oliveira R.^d,e^, Freixo J.^d,f^, Louro P.^d,e^, Quental R.^d,e^, Grangeia A.^d,e^, Pinheiro H.^a,b,g^, Fernandes S.^e^, Gullo I.^a,d,e^, Carneiro F.^a,d,e^, Castedo S.^a,d,e^, Drouet Y.^h^, Oliveira C.^a,b,d^

^a^ *i3S(Instituto de Investigação e Inovação em Saúde), U.Porto, Porto, Portugal;* ^b^ *Ipatimup(Institute of Molecular Pathology and Immunology), U.Porto, Porto, Portugal;* ^c^ *ICBAS(Instituto de Ciências Biomédicas Abel Salazar), U.Porto, Porto, Portugal;* ^d^ *Faculty of Medicine of the University of Porto, Porto, Portugal;* ^e^ *CHUSJ(Centro Hospitalar e Universitário de São João), Porto, Portugal;* ^f^ *Center for Predictive and Preventive Genetics, Institute for Molecular and Cell Biology, Porto, Portugal;* ^g^ *Department of Internal Medicine, Centro Hospitalar Tâmega e Sousa, Portugal;* ^h^ *Department of Public Health, Centre Léon Bérard, University of Lyon, France.*

**Introduction:** Hereditary Diffuse Gastric Cancer (HDGC) is an autosomal dominant syndrome mostly associated with pathogenic germline variants in the CDH1 gene conferring an increased risk for Diffuse Gastric Cancer (DGC) and Lobular Breast Cancer (LBC). The current understanding of HDGC families’ disease risk is limited due to its incomplete penetrance, different severity, and unknown mechanisms of disease progression. It is urgent to maximize disease prevention and to facilitate risk stratification of carrier that should undergo risk-reducing surgeries.

**Methodology:** We collected extensive demographic and clinical data from 11 families from the North region of Portugal carrying the pathogenic c.1901 C>T CDH1 founder variant (285 individuals with 171 genotyped cases). Using the genotype restricted likelihood (GRL) method, considering non-genotyped individuals and conditioning on all observed phenotypes and genotypes (n=285), we estimated the cumulative risk of DGC and LBC for the c.1901 C>T CDH1 variant.

**Results/Discussion:** This retrospective cohort study constitutes the largest dataset of probands and relatives for the most recurrent CDH1 variant in Europe. For the first time, we calculated less biased estimates for both HDGC-cancer phenotypes in carriers of a specific CDH1 variant, with the lowest cumulative risks ever described (DGC= 13.8% for both genders at 65 years old and LBC= 11.8% for female carriers only at 65 years old).

**Conclusion:** Our results reinforce the importance of collecting extended pedigree information for penetrance studies and support a strategy for creating clinical datasets that can be applicable to other recurrent variants from different hereditary syndromes.

#### P50 - BREAKING PERCEPTIONS: HOW TO EFFECTIVELY SUPPORT HEALTH PROFESSIONALS TO PARTICIPATE IN RESEARCH PROJECTS

Isabel S. Nunes^1,2^, Célia A. Soares^1,2,3,4^, Jorge D. da Silva^1,2,5,6^, Emídio V. Fernandes^2,7^, Márcia Barreiro^2,7^, Ana M. Capela^1^, Maria Abreu^1^, Cláudia F. Reis^1,2,5,6^, Ana M. Fortuna^1,2^, Natália Tkachenko^1,2^, Ana R. Soares^1,2^

^1^ *Primeiros Coautores; 1 Serviço de Genética Médica, Centro de Genética Médica Jacinto Magalhães, Centro Hospitalar Universitário de Santo António, Porto, Portugal;* ^2^ *Unit for Multidisciplinary Research in Biomedicine, Instituto de Ciências Biomédicas Abel Salazar/Universidade do Porto, Porto, Portugal;* ^3^ *Departamento de Ciências Médicas, Universidade de Aveiro, Aveiro, Portugal;* ^4^ *i3S – Instituto de Investigação em Saúde, Universidade do Porto, Porto, Portugal;* ^5^ *Life and Health Sciences Research Institute (ICVS), University of Minho, Campus de Gualtar, Braga 4710-057, Portugal;* ^6^ *ICVS/3B’s, PT Government Associate Laboratory, Braga, Guimarães, Portugal - Clinical Academic Center, Braga, Portugal;* ^7^ *Centro de Procriação Medicamente Assistida/ Banco Público de Gâmetas, Serviço de Ginecologia – Departamento da Mulher e da Medicina Reprodutiva, Centro Materno-Infantil do Norte, Centro Hospitalar Universitário de Santo António, Porto, Portugal.*

The community of health professionals (HP) performing research in Portugal and Europe, has been growing over the last few years. The engagement and active participation of HP in research projects led by basic scientists brings a clinical and practical perspective to basic research.

Clinical genetics is the area in which medical geneticists are responsible for diagnosing and advising on genetic diseases. They are responsible for determining the risk arising from family history, selecting cases in which further genetic investigations are needed and determining a surveillance plan and prevention measures appropriate to the risk level of disease.

These HP are in a privileged context to participate and lead research projects, due to their proximity to patients and to the opportunity to tackle clinically driven research questions. Moreover, clinical geneticists involved in research is an increasing necessity to facilitate the integration of research results in their healthcare practice and, ultimately, improve individual and community health outcomes.

Understanding how clinical geneticists perceive organizational support for research and how this influences their research engagement will shed light into this subject.

Our aim is to understand what the main enablers are and barriers for clinical geneticists successfully engage in research.

A systematic literature review allowed us to identify more than 30 enablers/barriers for the involvement of these HP in research, the most prominent of which are: time, recognition and awards, organizational support, accessible funding, consideration of research in career appraisal, training, and organizational culture.

The practical relevance of the identified variables will be assessed through a questionnaire survey, addressed to health professionals and hospital administrators in public health organizations from north to south of Portugal.

The main contribution of this project is to provide a better understanding of the impact that organizational support and management have in health professionals´ research engagement, and how to effectively support them within their clinical context to participate and/or lead research projects.

#### P51 - RETROSPECTIVE STUDY OF DIAGNOSTIC UTILITY OF USING ARRAY-CGH OR SEQUENCING PRENATALLY, IN FETUS WITH CENTRAL NERVOUS SYSTEM ABNORMALITIES

Vera MF Santos^1^, Fabiana Ramos^1,2^, Joaquim Sá^1,2^, Luís Abreu^2^, Filipa Nunes^2^, Filipa Marques^2^, Eulália Galhano^2^, Miguel Branco^2^, Raquel Pina^3^, Catarina Cerdeira^3^, Lina Ramos^1,2^

^1^ *Medical Genetics Unit, Hospital Pediátrico, Centro Hospitalar e Universitário de Coimbra, EPE;* ^2^ *Prenatal Diagnosis Unit, Maternidade Bissaya Barreto – Centro Hospitalar e Universitário de Coimbra, EPE;* ^3^*Pathological Anatomy Unit, Centro Hospitalar e Universitário de Coimbra, EPE*

**Introduction:** Central nervous system (CNS) abnormalities, represent a common and serious congenital malformation. In Portugal, its prevalence is 21 in 10 000 births, and many are detected in prenatal. Diagnose represents a challenge. Studies in the prenatal period should be efficient, economical and reliable to allow for proper genetic counselling. Previous studies have shown that the diagnostic rate of comparative genomic hybridization array (aCGH), is 6.5%3,4, and the diagnostic rate of exome sequencing (ES), varies from 8.5% to 24%4,5. Since we have expensive studies and limited resources, it is imperative to define well-founded strategies. The objective of this work is to assess the value of each of these techniques as a diagnostic tool, comparing the diagnostic yield of aCGH and ES, and associating it with the diagnostic rate.

**Methods:** Retrospective study of two years of evaluation, from January 1, 2021 to December 31, 2022. We compared 27 cases of fetuses following termination of pregnancy due to CNS abnormalities, identified by prenatal ultrasound, in the Prenatal Diagnosis Unit. Clinical findings were organized in isolated, multiple or multisystem. Data from clinical evaluations, prenatal ultrasound, autopsy and results from aCGH and/or sequencing were analysed.

**Results:** The study included 56 cases of fetuses following termination of pregnancy due to CNS abnormalities, focusing on 36 on which a genetic study was performed. Among all 36 cases, 9 CNS abnormalities were isolated, 12 were complex and 15 were multisystem. Array-CGH was performed in 25 cases and diagnosis was achieved in 6 cases (24%); ES was performed in 12 cases, and we obtained an increase of 28,6% (4) in the diagnostic rate: 20% (isolated), 25% (complex) and 66,7% (multisystem). An autopsy was performed on all fetuses and confirmed and detail the clinical findings. Relatively to the cases diagnosed, it also completed with new findings in other systems in 5 cases, and in 1 suggested a diagnosis, confirmed after by genetic study.

**Discussion:** In our study, the diagnostic rate of aCGH and ES results are slightly higher than in previous studies. ES had a similar detection rate in both groups, isolated and complex, but they are higher when compared with the detection rate of aCGH. The diagnosis rate is even more significant in multisystem cases, achieving 50% with aCGH and 66,7% with ES. In a general way, ES seems to be a potential method to improve the diagnosis, and performed after the aCGH leads to a significant increase in the diagnosis rate, mostly in multisystemic cases. However, we must take note that two years is a short period of evaluation, and our group is also limited. Further research is needed, particularly expanded to other units, for definitive conclusions, standardization of procedures and maximization of benefits and efficiency. Autopsy proved to be an excellent in the diagnostic process.

#### P52 - LDLR ACTIVITY AND CARDIOVASCULAR BURDEN IN PORTUGUESE FAMILIES WITH FAMILIAL HYPERCHOLESTEROLEMIA

Beatriz R. Miranda^1,2^, Ana M. Medeiros^1,2^, Ana C. Alves^1,2^, Mafalda Bourbon^1,2^

^1^ *Grupo de Investigação Cardiovascular, Unidade I&D, Departamento de Promoção da Saúde e Doenças Crónicas, Instituto Nacional de Saúde Doutor Ricardo Jorge, Portugal;* ^2^ *BioISI– Biosystems & Integrative Sciences Institute, Faculty of Sciences, University of Lisboa, Campo Grande, Lisboa*

**Introduction:** Familial hypercholesterolemia (FH) is the most common inherited disorder of lipid metabolism and is clinically characterized by elevated plasma cholesterol, which predisposes to cardiovascular disease (CVD). In nearly 90% of the cases, FH is caused by a pathogenic/likely pathogenic variant in the LDLR gene. In this work, we aimed to compare the cardiovascular burden in families from the Portuguese FH Study (PFHS) carrying different LDLR variants functionally studied.

**Methodology:** The PFHS database (containing clinical and molecular characterization of individuals referred to the PFHS) was consulted. Considering well-documented personal and familial history of CVD, a total of 246 PFHS families carrying LDLR causative variants (previously functionally characterized) were selected for this study.

**Results:** According to the results of functional assays reported, 47 different pathogenic/likely pathogenic variants (found in 617 subjects) were divided into 3 cut-offs of LDLR activity: <5% (n=15), 5-30% (n=16), and 30-70% (n=16). Within 80 families carrying variants with a LDLR activity of <5% (214 participants), 10% of individuals suffered at least one cardiovascular event (mainly myocardial infarction) at medium age of 44 years, and the majority reported familial history of CVD in more than 2 generations. In 115 families carrying variants with LDLR activity between 5-30% (280 participants), 7% of the subjects had a cardiovascular event at medium age of 41. It is relevant to note that the individuals presenting CVD had, specifically, variants showing less than 15% of activity. Despite comparatively fewer subjects (only 123) in 51 families carrying variants with a LDLR activity of 30-70%, 12% of them reported development of CVD at notably older age (medium of 51).

**Discussion:** Although the percentage of patients with premature CVD seemed to be very similar in the different groups, the mean age of onset is considerably higher in patients with a higher LDLR activity. To decrease the cardiovascular burden of individuals with FH, the early identification of these individuals and the functional characterization of variants, should be performed for a better and more personalized diagnosis and disease management.

#### P53 - UNRAVELLING THE GENETIC BASIS OF COMPLEX CLINICAL CASES OF HEMOGLOBINOPATHIES

Eduarda Silva^1^, Rita Pena^1^, Armandina Miranda^2^, Alcina Costa^2^, Gisela Gaspar^2^, Pedro Lopes^1^, Paula Faustino^1,3,4^

^1^ *Departamento de Genética Humana, Instituto Nacional de Saúde Doutor Ricardo Jorge, Lisboa;* ^2^ *Departamento de Promoção da Saúde e Prevenção de Doenças Não Transmissíveis, Instituto Nacional de Saúde Doutor Ricardo Jorge, Lisboa;* ^3^ *Grupo Ecogenética e Saúde Humana, Instituto de Saúde Ambiental, Faculdade de Medicina, Universidade de Lisboa, Lisboa;* ^4^ *Laboratório Associado TERRA, Faculdade de Medicina, Universidade de Lisboa, Lisboa*

Hemoglobinopathies encompass all genetic diseases of hemoglobin (Hb), the iron-containing oxygen-transport protein present in red blood cells. They occur due to mutations in globin genes or in their regulatory regions, and are classified as Hb variants and thalassemias. The aim of this work was to identify the molecular lesions in the origin of complex cases of hemoglobinopathies and understand the underlying pathophysiological mechanisms.

We investigated 15 clinical cases suspected of having one or more hemoglobinopathy, presenting with atypical hematological phenotypes. The study included the search for alterations in beta- and alpha-globin gene clusters by PCR, Gap-PCR, Sanger sequencing, and Multiplex Ligation-dependent Probe Amplification. In silico analyses were performed using Polyphen-2, SIFT, and varSeak.

Two beta-thalassemia carriers with abnormally low HbA2 level were found to have double heterozygosity for a mutation in HBB gene (c.92 + 1G>A, c.92 + 6T>C) and a delta-chain Hb variant (Hb A2-Yialousa). Another case was justified by a novel large deletion, which removes the entire beta-globin gene cluster as well as the olfactory receptor genes, OR52A1 and OR51V1. Changes in HbA2 values were also justified by a deletion that eliminates the HBD (Corfu deletion) or by the presence of the HbA2´variant. Atypically high levels of fetal Hb were explained by alterations in promoters of HBG genes (HBG1:c.-248C>G, HBG1:c.-228T>C, HBG2:c.-211C>T) or by deletions that remove both HBD and HBB (HPFH-1, HPFH-2). An even more complex case was originated by triple heterozygosity involving the Southeast Asian alpha-thalassemia deletion, the alpha-chain variant Hb Westmead, and the beta-chain variant HbE. As far as we know, this is the first case in which the three alterations were found in the same individual.

Individuals presenting abnormal phenotypes due to more than one hemoglobinopathy may be misdiagnosed if not correctly studied. Unravelling the genetic basis of complex clinical cases allows a better referral to genetic counselling, improves the understanding of the pathophysiology of the disease and its modifying factors, and may reveal new therapeutic targets.

#### P54 - DEVELOPMENT OF A PIPELINE THAT LINKS GENOMIC DATA WITH CT IMAGES OF HEAD AND NECK CANCER FOR THE IDENTIFICATION OF CLINICAL BIOMARKERS

Alexandre Costa^1^, Francisco Caramelo^2,3,4^, Francisco Marques^5^, Joana Barbosa Melo^1,3,4^, Isabel Marques Carreira^1,3,4^, Ilda Patrícia Ribeiro^1,3,4^

^1^ *Cytogenetics and Genomics Laboratory, Faculty of Medicine, University of Coimbra, Coimbra, Portugal;* ^2^ *Laboratory of Biostatistics and Medical Informatics, iCBR - Faculty of Medicine, University of Coimbra, Coimbra, Portugal;* ^3^ *University of Coimbra, Coimbra Institute for Clinical and Biomedical Research (iCBR) and Center of Investigation on Environment Genetics and Oncobiology (CIMAGO), Faculty of Medicine, Coimbra, Portuga;* ^4^ *CIBB – Centre for Innovative Biomedicine and Biotechnology, University of Coimbra, Coimbra, Portugal;* ^5^ *Department of Dentistry, Faculty of Medicine, University of Coimbra, Coimbra, Portugal*

**Introduction:** Head and neck cancer (HNC) is the 7th most common cancer and is also one of the most fatal cancers. Furthermore, as this cancer develops primarily in an area where several important human structures are located, such as the tongue, gums, pharynx, and vocal cords, patients who must undergo more aggressive treatments may lose, partially or totally, their ability to speak, swallow or chew.

This study aimed to develop a pipeline to check for differences in copy number variations between groups of HNC patients with similar radiomic features. An understanding of these differences can be used to develop more personalized treatments to reduce both the mortality and morbidity of this disease.

**Methods and Results:** For this study, a small dataset of CT images from nine patients with head and neck cancer was used. The pipeline begins with the segmentation phase, using a graphical user interface (GUI) designed for this purpose, of the most visible regions of the tumor in the available images of each patient. Radiomic features are then extracted from the segmented tumor regions that are used, in the next stage, to perform a clustering analysis enabling patients to be grouped into clusters. In the final stage, all the genes identified with copy number alterations, in the DNA extracted from the tumor tissue, using the array Comparative Genomic Hybridization technique are analyzed and it is checked which ones show a significant difference between clusters.

**Conclusion:** The development of this pipeline has provided a methodology that makes it possible to link copy number alterations to information present in CT images. This form of analyzing data can be applied to other situations provided that clinical images and genomic information is given, which makes it extremely versatile.

#### P55 - DISRUPTION OF POGZ AND SYNDROMIC INTELECTUAL DISABILITY: REPORT OF 4 PORTUGUESE CASES

Isabel Serra Nunes^1,2^, Jorge Diogo Silva^1,2,3,4^, Maria Abreu, Ana Catarina Prior^5^, Ana Fortuna^1,2^, Natalia Tkachenko^1,2^, Ana Rita Soares^1,2^

^1^ *Centro de Genética Médica Jacinto de Magalhães, Centro Hospitalar Universitário de Santo António (CHUdSA), 4099-001 Porto, Portugal;* ^2^ *UMIB— Unidade Multidisciplinar de Investigação Biomédica, ICBAS— Instituto de Ciências Biomédicas Abel Salazar, Universidade do Porto, 4050-345 Porto, Portugal;* ^3^ *Life and Health Sciences Research Institute (ICVS), University of Minho, Campus de Gualtar, Braga 4710-057, Portugal;* ^4^ *ICVS/3B’s, PT Government Associate Laboratory, Braga, Guimarães, Portugal - Clinical Academic Center, Braga, Portugal;* ^5^ *Unidade de Neurodesenvolvimento, Centro Hospitalar Universitário de Santo António (CHUdSA), 4099-001 Porto, Portugal.*

**Introduction:** CWhite–Sutton syndrome (WHSUS) is a monogenic, autosomal dominant neurodevelopmental disorder characterized by cognitive dysfunction, disruptive behaviors and dysmorphic features. The diagnosis is established in a proband with suggestive findings and heterozygous pathogenic variant in POGZ gene. To date, around 100 cases have been reported. Here we report 4 unrelated Portuguese individuals with heterozygous mutations in POGZ and compatible phenotype.

**Case Report:**

**Case 1**: Seven-year-old boy with neurodevelopmental delay, unspecific dysmorphisms and disruptive behaviour. Irrelevant family history. Genetic evaluation at 16 years old included Intellectual Disability (ID) gene panel - heterozygous pathogenic variant POGZ: c.3001C>T (Arg1001*), de novo.

**Case 2**: Seven-year-old boy referred for developmental delay, dysmorphic features, heteroaggressiveness and obesity. Family members with learning difficulties (LD). ID gene panel with pathogenic variant POGZ: c.1837del p.(His613Metfs*13) in heterozygosity. Parents refused testing.

**Case 3**: Boy with five years old with development delay, behaviour issues, facial dysmorphisms and obesity. Irrelevant family history. Evaluation at 12 years-old included a ID gene panel that revealed a heterozygous likely pathogenic variant in POGZ: c.3624del p.(Trp1208Cysfs*20), not present in the mother.

**Case 4**: Nine-year-old boy evaluated for ID, facial dysmorphisms and autism spectrum disorder. Family with LD. Genetic evaluation five years later included ID gene panel: variant of unknown significance POGZ: c.2459G>A (p.C820Y). Father is dead, mother’s study was negative.

**Discussion:** WHSUS is a neurodevelopmental disorder with unspecific phenotype and, thus, likely underdiagnosed. The vast majority of cases have truncating variant and missense variants’ role is still unclear. This type of variant is not clearly associated with cognitive impairment but appears to be associated with disruptive behaviors. With this small cohort description characterization, the authors aim to characterize the first cohort of WHSUS Portuguese patients and to highlight the high incidence and impact of obesity and behavioural disturbs in this syndrome.

#### P56 - CYTOGENETIC CHARACTERIZATION OF GLIOBLASTOMA CELLS – PRELIMINARY DATA

Ilda Patrícia Ribeiro^1,2,3^, Domingos Roda^4^, Cláudia Pais^1^, Alexandra Mascarenhas^1^, Pedro Veiga^1^, Isabel Marques Carreira^1,2,3^, Joana Barbosa Melo^1,2,3^

^1^ *Cytogenetics and Genomics Laboratory, Faculty of Medicine, University of Coimbra, Coimbra, Portugal;* ^2^*University of Coimbra, Coimbra Institute for Clinical and Biomedical Research (iCBR) and Center of Investigation on Environment Genetics and Oncobiology (CIMAGO), Faculty of Medicine, Coimbra, Portuga;* ^3^*CIBB – Centre for Innovative Biomedicine and Biotechnology, University of Coimbra, Coimbra, Portugal;* ^4^*Algarve Radiation Oncology Unit – Joaquim Chaves Saúde (JCS), Faro, Portugal*

**Introduction:** The cytogenomic analysis of tumor cell lines and primary cell cultures has proven invaluable in identifying genetic alterations associated with cancer development and progression, as well as in investigating novel therapeutic approaches. This study aimed to establish new primary cell cultures from glioblastoma tumor samples and to perform a cytogenetic characterization of both a commercial cell line and newly established primary cell cultures.

**Methods:** A commercial glioblastoma cell line, U87 from the ATCC, was used and one newly primary cell culture from a glioblastoma tumor sample was successful establish. The cells were grown in Dulbecco’s Modified Eagle’s Medium (DMEM) containing 10% fetal bovine serum and 1% penicillin and streptomycin. U87 cell line identity was confirmed by short tandem repeats profile. The cytogenetic characterization of cells was assessed by karyotype.

**Results and Discussion:** In terms of morphology, glioblastoma cells initially exhibited polymorphism, showing localized variations in cell density and irregular cytoplasmic extensions. Subsequently, they underwent a transformation characterized by a reduction in cytoplasmic extensions, taking on a spindle-shaped appearance dominated by small oval nuclei. Numerical and structural chromosomal abnormalities were found in both U87 cell line and primary cell culture. U87 is an hypertriploid cell line, with a modal chromosome number of 79. The most common rearrangements in this cell line are translocations, some in the centromere/near-centromeric regions, such as der(10)t(10;22)(p10;q10) and der(16)t(1;16)(p13.3;p13,2); and complex rearrangements involving three or more chromosomes, such as der(1)t(1,?)(p22.1;?) and der(20)t (20;1;14) (p10;q10q13). The primary cultured cells is a diploid sample, with a modal number of 46 chromosomes, being also the most commonly observed structural rearrangements translocations, such as t(1;12)(p36.3;q13.1), t(4;8)(p16.3;q21.3), and t(17;22)(q11.2;q13.3). The most frequently observed numerical rearrangements were trisomy or tetrasomy of chromosome 7 and monosomies of chromosomes 10 and 14. These preliminary findings improve our understanding of the molecular mechanisms underlying glioblastoma carcinogenesis process and lay the foundation for subsequent pharmacological studies. Further molecular studies and genotype phenotype correlations are in progress.

#### P57 - POI PATHWAY (WP5316) UNRAVELS A HIGH NUMBER OF FANCONI ANEMIA DNA REPAIR GENES IMPLICATED IN PRIMARY OVARIAN INSUFFICIENCY

Vanessa Sousa^1,2,3^, Bárbara Rodrigues^1,2,3^, Nuno Maia^1,2,3,4^, Isabel Marques^1,2,3^, Rosário Santos^1,2,3^, Friederike Ehrhart^5^, Paula Jorge^1,2,3^

^1^ *Laboratory Genetics Service, Genetics and Pathology Clinic, Centro Hospitalar Universitário de Santo António, Porto, Portugal.* ^2^ *UMIB-Unit for Multidisciplinary Research in Biomedicine, ICBAS-School of Medicine and Biomedical Sciences, University of Porto, Porto, Portugal.* ^3^ *ITR-Laboratory for Integrative and Translational Research in Population Health, Porto, Portugal.* ^4^ *School of Health, Polytechnic Institute of Porto, Porto, Portugal.* ^5^ *Department of Bioinformatics-BiGCaT, NUTRIM/MHeNs, Maastricht University, The Netherlands*

**Introduction:** Primary Ovarian Insufficiency (POI) is one of the most common causes of female infertility, affecting about 1-3.7% of women under 40 years old. POI often leads to permanent sterility and severe comorbidities such as osteoporosis and cardiovascular diseases and neurodegenerative conditions. Most of the cases remain without an identified molecular cause, making infertility a major public health problem. Recent studies have identified pathogenic variants in DNA damage/repair and meiotic genes in POI. However, the underlying mechanisms remain unknown. Aiming to identify which repair mechanisms are implicated, we used the POI pathway recently developed by our group, to understand the synergies between POI and Fanconi Anemia (FA), a well-known disease caused by defects in DNA repair.

**Methodology:** The POI pathway created and uploaded to WikiPathways database, was analyzed. The updated human DNA repair genes table was used to identify the DNA damage/repair genes. Network analysis of the POI and FA pathways were performed in Cytoscape software. G:Profiler database was used to perform the functional profile of the shared genes.

**Results:** The molecular pathway includes 176 POI-associated genes, in which 15% are DNA repair genes, most of them associated with homologous recombination and FA. The comparison between POI and FA pathways revealed an overlap of eight genes associated with protein monoubiquitination, DNA repair, interstrand crosslink repair and chromosome stability: BRCA1, BRCA2, FANCA, FANCC, FANCL, FANCG, FANCM and NBN.

**Discussion:** The POI pathway allowed the contextualization of the biological and molecular data enabling large-scale data analysis, integration, and interpretation. The comparison with FA pathway revealed an association between DNA repair and infertility. Our comparative analysis led us to hypothesize that FA patients have impaired FANCI-FANCD2 mono-ubiquitination which may affect the DNA damage repair efficiency and meiotic fidelity in oocytes, leading to untoward consequences on gonadal function. In fact, some FA patients exhibit symptoms of POI and infertility. The identification of variants in the FA genes (e.g. FANCD1/ BRCA2, FANCM, and FANCL) in patients with POI, also supports our results. Since FA is highly associated with cancers, it is tempting to speculate that other DNA repair and cancer susceptibility genes should be explored in the context of POI.

#### P58 - PRECISION GENOMIC MEDICINE OF GENOMIC DISORDERS

Dezso David^1^, Joana Fino^1^, Sofia Nunes^2^, Márcia Saraiva^1^, André Travessa^3^, Oana Moldovan^3^, Sofia Doria^4^, Natália Oliva-Teles^5^, Nataliya Tkachenko^5^, João Freixo^6^, Márcia Rodrigues^3^, Cynthia C Morton^7^

^1^ *Departamento de Genética Humana, Instituto Nacional de Saúde Doutor Ricardo Jorge, Lisboa;* ^2^ *Serviço de Genética Medica, Centro Hospitalar Universitário Lisboa Central, Norte, Hospital Dona Estefânia, Lisboa;* ^3^ *Serviço de Genética Medica, Centro Hospitalar Universitário Lisboa Norte, Hospital de Santa Maria, Lisboa;* ^4^ *Departamento de Patologia, Faculdade de Medicina da Universidade do Porto, Porto;* ^5^ *Centro de Genética Médica Jacinto de Magalhães (CGM), Centro Hospitalar Universitário do Porto, Porto;* ^6^ *Centro de Genética Preditiva e Preventiva, Instituto de Biologia Molecular e Celular, Porto;* ^7^ *Department of Obstetrics and Gynecology and of Pathology, Brigham and Women’s Hospital, Harvard Medical School, Boston, USA; Broad Institute of MIT and Harvard, Cambridge, USA; Manchester Center for Audiology and Deafness, University of Manchester, UK*

**Introduction:** Subjects with disease-associated cytogenomically visible or cryptic balanced or unbalanced chromosomal or genomic structural variants (SVs), known as genomic disorders (GD), are ideal for application of a genome sequencing (GS) based precision genomic medicine (PGM) approach. Furthermore, such SVs offer unique resources for identification or confirmation of disease associated pathogenic genomic elements that otherwise would be difficult or impossible to detect and study. A GS approach with high physical coverage and sequencing-read depth, allowing identification of the full spectrum of genomic and genetic variants is a prerequisite for a PGM approach.

**Methodology:** We applied large-insert, short-insert, low-pass and mate-pair GS for application of the preconceived PGM approach in a group of 29 and 11 probands with simple and complex chromosomal SVs (cxCSVs), respectively. This was complemented by a robust genotype-phenotype correlation analysis, including phenotypic similarity search and reverse clinical phenotyping of the subjects and their family members. Of the analysed 40 subjects, four were clinically normal and the remaining presented unselected mainly neurodevelopmental clinical phenotypes of variable severity.

**Results:** In subjects with simple CSVs (SCSVs), pathogenic gene disruption, chromosomal position effect, single nucleotide variants (SNVs) and combined multigenic or multilocus alterations were the underlying causes of the clinical phenotypes. Over 64 % of the SCSVs were classified as pathogenic whereas 32% as likely pathogenic. Presently, chromosomal position effect can be ascertained based on three previously reported cases or hypothesized merely based on convergent genomic evidence. Altogether, a position effect was observed in 33% of the SCSVs. Over 90 % of the cxCSVs have been classified as pathogenic and multilocus or multigenic alterations including more frequent findings of copy number variations in this group. Furthermore, several unreported disease-causing candidate genes or variants were identified.

**Conclusion:** We implemented a PGM approach, leading to improved genomic healthcare of patients with GDs and their families and improved annotation of the morbid human genome. We continue our efforts for clinical implementation of this approach.

#### P59 - INTERPRETING GENETIC TESTING FOR INHERITED CARDIAC DISORDERS: UNCOVERING NUANCES BETWEEN ‘NEVER’ AND ‘ALWAYS’

Diana Antunes^1,2,3^, Rafael Graça^1^, Inês Custódio Santos^3^, Silvia Aguiar Rosa^4^, Catarina Silveira^1^, Sofia Pérez^3^, Yuri Chiodo^1^, Maria Carmo-Fonseca^5^

^1^ *GenoMed - Diagnósticos de Medicina Molecular, S.A, Lisboa, Portugal*, ^2^ *Santa Marta Hospital, Medical Genetics Department, Lisboa, Portugal*, ^3^ *Dona Estefânia, Medical Genetics Department, Lisboa, Portugal*, ^4^ *Santa Marta Hospital, Cardiology Department, Lisboa, Portugal*, ^5^ *Instituto de Medicina Molecular João Lobo Antunes, Faculdade de Medicina da Universidade de Lisboa, Portugal.*

**Introduction:** The “Expert Consensus Statement on the state of genetic testing for cardiac diseases”, published in 2022, appeared to be converging in a downgrade for genetic testing in hereditary cardiac diseases (HCD), supporting target testing in well-defined phenotypes.

**Methods:** Retrospective analysis of genetic testing carried out for suspected HCD between November 2015 and August 2023. The main goal was to validate in-house results, but also to evaluate the applicability of the 2022 Consensus.

**Results:** 978 genetic tests were categorized in: Hypertrophic Cardiomyopathy (HCM, N=395), Dilated Cardiomyopathy (DCM, N=274), Brugada Syndrome (BS, N=117), Arrhythmogenic Cardiomyopathy (ACM, N=48), Unspecified Cardiomyopathy (uCM, N=40), Unexplained Cardiac Arrest (uCA, N=27), Rhythm Disorders (RD, N=26), Non-compaction cardiomyopathy (NCM, N=24), and Long QT Syndrome (LQT, N=23).

Pathogenic (P) or likely pathogenic (LP) variants were identified in 209 patients with an overall diagnostic yield of 21.37% (N=209): ACM 31.25% (N=15), NCM 29,17% (N=6), HCM 25.32% (N=100), RD 23.08% (N=6), DCM 22.71% (N=62), uCA 18.52% (N=5), uCM 12.5% (N=5), LQT 17.39% (N=4), and BS 4.27% (N=5).

P/LP variants were identified in the following genes for HCM: ACTN2, ALPK3, CSRP3, FHOD3, GLA, KCNQ1, MYBPC3 (35%, N=35), MYH7 (31%, N=31), MYL2, PRKAG2, RBM20, RYR2, SLC25A4, TNNC1, TNNI3, TNNT2, TPM1, TRIM63 – and for DCM: DSP, FLNC, GLA, LMNA (11.29%, N=7), MYBPC3, MYH7, PKP2, RBM20, RYR2, SCN5A, TCAP, TNNT2, TPM1, TTN (38.71%, N=24), TTR (6.45%, N=5).

Double genetic diagnoses (GD) were found in 2.39% of cases (N=5) - HCM 3% (N=3) and DCM 3.23% (N=2), P/LP copy number variants were identified in 3 cases – 1,4%.

**Discussion:** These results revealed GD for HCM and BS lower than expected. More than half of the HCM GD were attributed to MYBPC3 and MYH7, while all BS cases were associated to SCN5A. The most prevalent DCM GD, as anticipated, was TTN.

P/LP variants were found in genes not covered by the 2022 Consensus, such as GLA in HCM or TTR in DCM. Although the guidelines recommend restraining from genetic testing in non-well defined phenotypes, P/LP variants were detected at significant rates in conditions categorized as less consensual such as uCM, RD and NCM.

An overall diagnostic yield >20% for patients suspected of having HCD, underscores the urgency of mainstreaming genetic testing.

While we acknowledge that ‘more is not always more’, as large panels can yield uncertain results, as we enter early stages of genomic screenings, we must evaluate the potential of opportunistic screening.

#### P60 - COPY NUMBER VARIANTS (CNVS) AND SYNDROMIC OBESITY: CLINICAL AND GENOMIC CHARACTERIZATION OF A PORTUGUESE PAEDIATRIC COHORT

Ana Mafalda Gonçalves^1^, Mariana Val^2^, Susana I. Ferreira^2^, Jorge M. Saraiva^1,3,5^, Isabel M. Carreira^2,4^, Joana Rosmaninho-Salgado^1^

^1^ *Serviço de Genética Médica, Hospital Pediátrico, Centro Hospitalar e Universitário de Coimbra;* ^2^ *Laboratório de Citogenética e Genómica, Faculdade de Medicina, Universidade de Coimbra;* ^3^ *Clínica Universitária de Pediatria, Faculdade de Medicina, Universidade de Coimbra;* ^4^ *Centro de Inovação em Biomedicina e Biotecnologia (CIBB), iCBR - CIMAGO – Faculdade de Medicina, Universidade de Coimbra;* ^5^ *Centro Académico Clínico de Coimbra, Coimbra, Portugal.*

Syndromic obesity is characterised by a combination of early-onset obesity and other clinical findings, most frequently intellectual disability (ID) or psychomotor developmental delay (PDD). The genetic causes of hereditary obesity remain largely unknown, prompting a necessity for a deeper understanding of its mechanisms and a detailed description of clinical features.

Different studies identified large copy number variants (CNV’s) in groups of patients with syndromic obesity [1,2,3,4], suggesting that structural defects might correspond to a substantial fraction of these cases. This study aims to expand the knowledge about the CNV’s most frequently associated with these phenotypes in a Portuguese paediatric cohort.

A retrospective analysis of clinical features and genomic findings was performed in a group of individuals referred for obesity and ID/PDD. We selected those who had been tested for CNV’s by 180K oligonucleotide microarray-based comparative genomic hybridisation (aCGH) as first line of diagnosis. The clinical features of those with pathogenic (P) or likely pathogenic (LP) variants were analysed. All variants of uncertain significance were excluded from this group.

A total of 133 cases were analysed (68 female) with a mean age of 13.5 ± 8.5 years. A total of 26 (19.5%) individuals (16 female) had P or LP variants (9 deletions and 7 duplications). The most frequent variants observed in this cohort were deletion 16p11.2 (n=8, 30.8%) and deletion 15q11.2 (n=2, 7.7%). The average BMI was 33.48 Kg/m2. In all cases, we identified variants previously associated with obesity and/or neurodevelopmental disorders. In addition to obesity and ID/PDD, 18 cases had other clinical features: dysmorphisms (n=13), congenital malformations (n=12) or epilepsy (n=5). Eight (30.8%) identified CNVs were inherited from one progenitor and 5 (19.2%) of those segregated with obesity.

We observed that 19.5% of cases in our cohort had at least one P or LP CNV associated with obesity and ID/PDD. This result is consistent with the diagnostic yield found in literature [1,2,3,4].

Array-CGH in patients with syndromic obesity allowed the discovery of CNV’s that hadn’t been previously known, but the actual detection rate has not been conclusively established. With these results, we hope to contribute to a list of genomic regions of interest and to the future development of a more streamlined approach to diagnosing syndromic obesity.

**References:**

1- D’Angelo CS et al (2014);

2-Sadia Saeed et al (2020);

3-Maria Pettersson et al (2017);

4-Micleaa et al; (2019)

#### P61 - CHOLESTATIC DISEASE IN THE NEWBORN AND CHILD: CLINICAL AND MOLECULAR CHARACTERIZATION OF A GROUP OF PATIENTS

Catarina S. Rosas^1^, Isabel Gonçalves^2,3^, Sandra Ferreira^2,3^, Susana Nobre^2^, Isabel Alonso^4^, João P. Freixo^4^, Lina C. Ramos^1,5,6^

^1^ *Medical Genetics Unit, Hospital Pediátrico, Centro Hospitalar e Universitário de Coimbra;* ^2^ *Paediatric Hepatology and Liver Transplant Unit, Hospital Pediátrico, Centro Hospitalar e Universitário de Coimbra;* ^3^ *ERN RARE-LIVER;* ^4^ *Center for Predictive and Preventive Genetics, Institute for Molecular and Cell Biology;* ^5^ *Faculty of Health Sciences, Universidade da Beira Interior, Covilhã, Portugal;* ^6^ *ERN ITHACA*

**Introduction:** Cholestatic jaundice is a common presenting feature of hepatobiliary or metabolic dysfunction in the newborn and young infant. The rapid evaluation to determine the etiology is relevant to decide for medical or surgical interventions and to optimize outcomes. The interaction between a Medical Genetics Unit and a Paediatric Hepatology and Liver Transplant Unit led to the development of a project which included the molecular etiological study of patients with neonatal or childhood cholestasis and eventual establishment of phenotype-genotype correlations. We aim to present preliminary results obtained to date.

**Methodology:** Data collection involving family interviews, patient objective examination and clinical records consultation. Clinical data included pre and postnatal history and family history, the presence of extrahepatic bile duct atresia and the need for liver transplantation, current clinical condition and analytical and genetic testing results, considering retrospective and prospective molecular characterization, through WES-based multigene panel design, bioinformatic reanalysis and/or update, as well as testing upgrading to whole exome sequencing or familial segregation studies, if relevant.

**Results:** A total of 163 patients have been included, 77 females (47%) and 86 males (53%), 7 of which have unfortunately passed away. Current ages (n=156) range from 0 to 32 years old (median of 11). Seventy patients presented with extrahepatic biliary atresia, 8 of which with associated extrahepatic clinical features. Etiological diagnosis was attributed to 36 patients, with improvement after family segregation studies were performed. In addition, 65 variants were identified in heterozygosity in genes associated with autosomal recessive inheritance, the most prevalent being the SERPINA1 gene (n=17).

**Discussion:** The multidisciplinary approach to cholestatic disease led to the etiological diagnosis in a large number of cases, even in some currently adult transplanted patients. Diagnoses such as Kabuki Syndrome 1 have been achieved through whole exome sequencing. The re-evaluation of complex patients with cholestatic disease and the follow-up of phenotypic evolution, sometimes irrelevant in the initial observation, are fundamental in the pursuing of an etiological diagnosis and to allow for specific genetic counselling. The number of carriers for recessive inheritance diseases was significant. The prevalence of these variants in the Portuguese population is not known, which might be a challenge for the future.

#### P62 - THE RARE TUMOUR RISK SYNDROMES BATTLE: THE CASE OF PARALLEL TRAJECTORIES AND CARE PATHWAYS FOR HDGC

Liliana Sousa^1,2,3,4^, Luzia Garrido^1,2,3^, Ricardo Amorim^5^, Sara Pereira^2^, Ana Mamede^2^, Bárbara Peleteiro^1,3,5,6^, Susy Costa^1,5^, Renata Oliveira^1^, Pedro Louro^1^, Margarida Marques^1^, Raquel Guimarães^1^, Elsa Madureira^1^, André Magalhães^1^, Fabiana Sousa^1,3,5^, Nuno Teixeira Tavares^1^, Manuela Baptista^1^, Daniela Almeida^1^, Sérgio Castedo^1,2,5^, Fátima Carneiro^1,2,3,5^, Ana Azevedo^1,3,5,6^, Carla Oliveira^1,2,3,5^

^1^ *Centro Hospitalar Universitário São João (CHUSJ), Porto, Portugal;* ^2^*Instituto de Investigação e Inovação em Saúde (i3s) & Institute of Molecular Pathology and Immunology of the University of Porto (Ipatimup), Porto, Portugal;* ^3^*European Reference Network on Genetic Tumour Risk Syndromes (GENTURIS);* ^4^*Department of Economics and Economic Policies Research Unit (NIPE), University of Minho, Braga, Portugal;* ^5^*Faculty of Medicine of the University of Porto (FMUP), Porto, Portugal;* ^6^ *Laboratory for Integrative and Translational Research in Population Health (LA/P/0064/2020), Instituto de Saúde Pública da Universidade do Porto*)

**Introduction:** Rare tumour risk syndromes (RTRS) are rare diseases, affecting 5 per 10.000 people or less and caused by heritable genetic variants, and the lifetime risk to develop various cancers can be as high as 100%.

CDH1 germline variants are the major cause of Hereditary Diffuse Gastric Cancer (HDGC). Variant carriers are at risk for diffuse gastric cancer (both genders) and lobular breast cancer (females) in adulthood. Cancer lifetime risks are greater than 50% by age 80, and higher for females.

Most healthcare organizations are focused on treatment of symptomatic HDGC patients. However, intensive surveillance and risk reduction surgery in asymptomatic carriers have shown to be effective, when performed in expert centres.

Mapping the specific care pathway for HDGC and patients’ clinical trajectories, that will allow development of an IT tool for collection of real patients’ clinical data and care pathway associated healthcare costs/prices for cost-effectiveness studies.

The HDGC optimized care pathway was defined by health professionals from ERN-GENTURIS Healthcare Provider CHUSJ and i3S scientists, with expertise in HDGC, and included: HDGC patients’ settings, condition-specific care pathways, individual clinical trajectories, and clinical procedures for diagnosis, surveillance, prevention and treatment.

We identified 22 individual clinical trajectories for non-carriers, asymptomatic carriers, and carriers affected with lobular breast cancer, diffuse gastric cancer or a combination of both. Regarding HDGC care pathway related procedures, we grouped a total of 59 procedures (medical/surgical/tests/consultations) into nine different modules: initial genetic diagnosis, post-genetic testing, surveillance protocol, risk-reduction protocol, staging protocol, early therapeutic protocol, therapeutic protocol, palliative care protocol and post-surgical follow-up.

We gathered relevant information to construct a HDGC-specific matrix displaying individual patients’ trajectories and pathway of care related procedures that will drive a cost-effectiveness analysis to demonstrate the value of preventive care in HDGC patients.

The ambition of the PREVENTABLE project is to merge specialized clinical knowledge on 8 RTRS pathways of care, real-life clinical data from patients and experiences from professionals and patients, with health economic models and social sciences approaches to estimate the cost-benefit of risk-reduction interventions in RTRS patients.

**Acknowledgement:** PREVENTABLE is funded by the EU under Grant Agreement number 101095483.

#### P63 - RELEVANCE OF MULTIGENE PANELS IN THE MOLECULAR DIAGNOSIS OF PATIENTS WITH DISORDERS OF SEXUAL DEVELOPMENT

Iris Pereira-Caetano^1^, Joana Mendonça^1^, Alice Mirante^2^, Rita Cardoso^2^, Márcia Rodrigues^3^, João Paulo Oliveira^4^, Ana Grangeia^4^, David Barbosa^5^, Luís Vieira^1,6^, João Gonçalves^1,6^

^1^ *Human Genetics Department, National Institute of Health Dr Ricardo Jorge, Lisbon, Portugal.* ^2^ *Pediatric Endocrinology, Diabetes and Growth Unit, Hospital Pediátrico de Coimbra, Centro Hospitalar e Universitário de Coimbra, Portugal;* ^3^ *Medical Genetics Service, Departamento de Pediatria, Hospital de Santa Maria, Centro Hospitalar Universitário de Lisboa Norte, Portugal;* ^4^ *Medical Genetics Service, Centro Hospitalar Universitário de São João, Porto, Portugal;* ^5^ *Endocrinology and Diabetes Service, Hospital Garcia de Orta, Almada, Portugal;* ^6^ *Center for Toxicogenomics and Human Health, Nova Medical School, Lisbon, Portugal. Support: FCT/MCTES, Projects - ToxOmics and Human Health (UIDB/00009/2020) and GenomePT (POCI-01-0145-FEDER-022184).*

**Introduction:** Next generation sequencing (NGS) is increasingly used in the molecular diagnosis of rare diseases, such as disorders of sexual development (DSD), allowing the analysis of a greater number of genes in a single assay, providing faster results with reduced costs. DSD patients may present high phenotypic overlap (genital ambiguity, sex reversal, delayed/absent puberty, infertility) posing challenges to clinical diagnosis. NGS technology using multigene panels has higher hypothesis to identify the genetic cause and novel genetic variants in a large number of cases.

**Methodology:** 33 genomic DNA samples from DSD patients were sequenced on MiSeq using the Ampliseq technology (Illumina). A customized gene panel covering 40 genes was used to prepare the libraries of the target sequences. The 40 genes were subdivided into 5 subpanels: primary sex determination, sex differentiation, hypogonadotropic hypogonadism/infertility, steroidogenesis and premature ovarian insufficiency. Variant classification, according to ACMG-AMP, was based on bioinformatics tools (ex. VEP, HSF, VarSome, Alamut) and databases (gnomAD, HGMD, ClinVar, dbSNP). Pathogenic, Likely pathogenic and VUS variants were confirmed by Sanger sequencing.

**Results:** 16 of the 33 patients were previously studied in our laboratory with negative results for the main genes associated with DSD (SRY, AR, ANOS1, GNRHR, CYP21A2). We identified 9 causative variants (8P/1LP) in 7 patients (21.2%) in AR, HSD17B3, LHCGR, FGFR1 and NR5A1. One patient was a compound heterozygous for HSD17B3 and another simultaneously homozygous for LHCGR and hemizygous for AR. The remaining 5 were homozigous, heterozigous or hemizygous for HSD17B3, LHCGR, NR5A1, FGFR1 or AR. One VUS, FGFR1:c.566G>A p.Arg189His, requires familial studies to revaluate its pathogenicity.

**Discussion:** Multigene NGS studies allows to increase the rate of variant detection, mainly in genes not included in a first molecular approach. It also contributes to establishing or confirming the clinical diagnosis, assisting in decisions regarding the treatment and reproductive management of patients and families, as well as in genetic counselling.

#### P64 - HEREDITARY ANGIOEDEMA – THE OPTIMISATION OF MOLECULAR STUDIES IN DIFFERENTIAL DIAGNOSTICS

Ivo Barreiros^1^, José P. Silva^1^, Catarina S. Pinto^1^, Patrícia Martinho^1^, Madalena Calheiros^2^, Teresa Fidalgo^1^

^1^ *Unidade Funcional de Hematologia Molecular, Serviço de Hematologia Clínica, Centro Hospitalar e Universitário de Coimbra, Coimbra, Portugal;* ^2^ *Hospital de Braga, Braga, Portugal*

**Introduction:** Hereditary angioedema (HAE) is a rare autosomal dominant disorder which affects ~1 in 50 000 individuals. It’s mainly characterized by recurrent episodes of the accumulation of extravascular fluid in various tissues causing sudden swelling of the face, airway, limbs, or gastrointestinal tract. C1-INH (C1 esterase inhibitor) is a major regulator of key enzymes involved in bradykinin generation which increases vascular permeability and allows fluids to flow into extracellular space. Mutations in SERPING1, the gene encoding C1-INH, are responsible for the majority of HAE cases and result in reduced levels of functional plasma C1-INH. This deficiency leads to two C1-INH HAE variants: type 1 (low synthesis) and type 2 (non-functional protein). HAE has also been associated with a lower prevalence in patients with normal C1-INH activity, HAE type 3. This deficiency results from an altered bradykinin system and is associated with variants in the F12, ANGPT1 and PLG genes.

The aim of this work was to carry out the molecular diagnosis of patients with clinical suspicion of HAE.

**Methodology:** This study included 10 individuals, 8 probands and 2 relatives (3M,7F; 11-45Y). Molecular studies were performed by Sanger sequencing of SERPING1, F12, PLG, and ANGPT1; 3D modelling of mutant C1-INH (PDB: 5DU3) using the PyMOL; variant classification according to ACMG guidelines.

**Results:** In this study, despite being a small cohort, several types of HAE were found (Table 1): variants in SERPING1 (2 probands) 1 HAE type 1 c.310C>T (p.Gln104*), pathogenic variant described with a severe HAE; c.639C>A (p.Phe213Leu) variant was classified as a variant of uncertain significance, but 3D modelling showed a conformational change in the α-helix E, near the shutter region, the functional domain responsible for forming the complex with C1. This could affect the function of C1-INH, albeit with mild phenotype; 4 had HAE type 3 and no pathogenic variants were identified in genes studied; the remaining 2 had reduced C1-INH levels without variants in SERPING1 gene and were likely acquired angiodema.

**Discussion:** A good genotype-phenotype correlation in patients with SERPING1 variants was observed. Molecular study of these patients with variable penetrance identifies asymptomatic carriers and, by exclusion, acquired forms. The challenge remains for HAE type 3, where an increasing number of candidate genes may require clinical exome analysis by NGS. This two-step strategy appears to be the most effective for studying HAE patients, allowing more precise diagnosis and more appropriate treatment.

#### P65 - ASSESSING PARKINSON’S DISEASE AND RELATED DISORDERS THROUGH MOLECULAR DIAGNOSIS: EXPLORING THE MUTATIONAL SPECTRUM AND CLINICAL IMPLICATIONS FROM A LABORATORY PERSPECTIVE

Patrícia Isabel Marques^1,2,*^, Ana Filipa Brandão^1,2,*^, Alexandra Manuel Lopes^1,2^, Sara Morais^1,2^, Joana Sá^1,2^, Ana Lopes^1,2^, Rita Bastos-Ferreira^1,2^, Diana Pinto^1,2^, Miguel Alves-Ferreira^1,2,3^, Fátima Lopes^1,2^, Paulo Silva^1,2^, Filipe Alves^1,2^, Marina Magalhães^4^, Nuno Vila Chã^4^, Alexandre Mendes^4^, Joana Damásio^4^, Ana Graça Velon^5^, João Lourenço^6^, Miguel Dias Grunho^7^, Maria José Rosas^8^, Ana Oliveira^8^, Rui Araújo^8^, Miguel Gago^9^, Graça Duarte Sousa^10^, Maria Margarida Calejo^11^, Sandra Moreira^11^, Gisela Carneiro^12^, Margarida Rodrigues^12^, Leonor Correia Guedes^13^, Miguel Coelho^13^, Mário Miguel Rosa^13^, Anabela Ferreira Valadas^13^, Daniela Pimenta Silva^13^, Rui Duarte Barreto^13^, Maria João Nabais Sá^1,2^, João Parente Freixo^1,2^, Jorge Sequeiros^1,2,3^, Jorge Oliveira^1,2^

^1^ *CGPP-IBMC – Centro de Genética Preditiva e Preventiva, Instituto de Biologia Molecular e Celular, Universidade do Porto, Porto, Portugal;* ^2^ *i3S – Instituto de Investigação e Inovação em Saúde, Universidade do Porto, Porto, Portugal;* ^3^ *ICBAS School of Medicine and Biomedical Sciences, Universidade do Porto, Porto, Portugal;* ^4^ *Serviço de Neurologia, Hospital Geral de Santo António, Centro Hospitalar Universitário de Santo António, Porto, Portugal;* ^5^ *Serviço de Neurologia, Hospital de São Pedro, Centro Hospitalar de Trás-os-Montes e Alto Douro, Vila Real, Portugal;* ^6^ *Serviço de Neurologia, Hospital de Santo António dos Capuchos, Centro Hospitalar Universitário de Lisboa Central, Lisboa, Portugal;* ^7^ *Serviço de Neurologia, Hospital Garcia de Orta, EPE, Lisboa, Portugal;* ^8^ *Serviço de Neurologia, Centro Hospitalar Universitário de São João; e Faculdade de Medicina da Universidade do Porto, Porto, Portugal;* ^9^ *Serviço de Neurologia, Unidade Hospitalar de Guimarães, Centro Hospitalar do Alto Ave, Guimarães, Portugal;* ^10^ *Serviço de Neurologia, Centro Hospitalar de Vila Nova de Gaia/Espinho, EPE - Unidade I, Vila Nova de Gaia, Portugal;* ^11^ *Serviço de Neurologia, Hospital Pedro Hispano, Unidade Local de Saúde de Matosinhos, Matosinhos, Portugal;* ^12^ *Serviço de Neurologia, Hospital de Braga, Braga, Portugal;* ^13^ Serviço de Neurologia, Hospital de Santa Maria, Centro Hospitalar de Lisboa Norte, Lisboa, Portugal^*^Contributed equally

**Introduction:** Parkinson disease (PD) is a debilitating neurodegenerative disease, characterized by rest tremor, rigidity, bradykinesia, postural instability and dementia. Parkinsonism refers to a broad range of diseases that share clinical features with PD. While mostly sporadic, about 5-10% of cases can be attributed to monogenic causes. Among familial cases, disease-causing variants in LRRK2 account for ~5% of cases, while diallelic variants in PRKN are responsible for up to 50% of early-onset cases.

**Methodology:** To describe the mutational spectrum of PD-related genes and the diagnostic yield of a WES-based multigene panel for PD, we reviewed a cohort of 318 patients studied at our laboratory (2018-2023).

**Results:** A conclusive molecular diagnosis was established in 34 cases harbouring pathogenic (PAT) or likely pathogenic (L-PAT) variants at 9 different loci. These comprised PRKN, PINK1, VPS13C, LRRK2 and SNCA (in PD); PRKRA, ATP1A3 and DCTN1 (in Parkinsonism); and CSF1R (gene associated to a differential diagnosis of PD). Among all these, the most frequent mutated gene was LRRK2, in which variant NM_198578.4 (LRRK2): c.6055G> A was observed in 19 cases. Regarding the other affected genes, PRKN had variants in 8 patients, DCTN1 in 3 cases and variants at other genes occurred in one patient each. Of the remaining 284 patients, 151 (47.5%) had no variants reported, while 133 (41.8%) had variants of unknown significance (VUS) or one PAT/L-PAT heterozygous variant without a second PAT/L-PAT allele in recessive forms. Among these, 2 were heterozygotes for NM_004562.3(PRKN):c.719C>T, previously described in late-onset PD and apparent autosomal dominant inheritance, further documenting the difficulties in variant interpretation and reporting.

**Discussion:** The diagnostic yield of all patients referred for WES-based panels was 10.7%. In the 41.8% of the patients with VUS, clinical revaluation and/or segregation analysis within the family may provide data for reclassification and, eventually, lead to a definitive diagnosis.

Also, genetic testing using WES-based multigene panels grant the opportunity for reanalysis of inconclusive cases, as further evidence or new genes are identified.

#### P66 - ANALYSIS OF GENETIC TESTING RESULTS IN 292 PATIENTS WITH HEREDITARY DYSTONIA: MUTATIONAL SPECTRUM AND DIAGNOSTIC YIELD

Diana Pinto^1,2,*^, Fátima Lopes^1,2,*^, Liliana Rocha^1,2,*^, Alexandra M. Lopes^1,2^, Sara Morais^1,2^, Ana Filipa Brandão^1,2^, Ana Lopes^1,2^, Miguel Alves-Ferreira^1,2,14^, Joana Sá^1,2^, Marina Magalhães^3^, Ana Sofia Morgadinho^4^, Ana Graça Velon^5^, Joana Damásio^1,2,6^, Miguel Coelho^7^, Sofia T. Duarte^8^, Inês Carrilho^9^, João Lourenço^10^, Miguel Leão^11^, Diogo Carneiro^4^, Inês Antunes Cunha^4^, Maria José Rosas^12^, Sofia Quintas^13^, Sónia Figueiroa^9^, Paulo Silva^1,2^, Filipe Alves^1,2^, Maria João Nabais Sá^1,2^, João Parente Freixo^1,2^, Jorge Sequeiros^1,2,14^, Jorge Oliveira^1,2^

^1^ *CGPP-IBMC – Centro de Genética Preditiva e Preventiva, Instituto de Biologia Molecular e Celular, Universidade do Porto, Portugal;* ^2^ *i3S – Instituto de Investigação e Inovação em Saúde, Universidade do Porto, Portugal;* ^3^ *Departamento de Neurologia, Centro Hospitalar Universitário de Santo António, Porto, Portugal;* ^4^ *Departamento de Neurologia, Centro Hospitalar e Universitário de Coimbra, Coimbra, Portugal;* ^5^ *Departamento de Neurologia, Centro Hospitalar Trás-os-Montes e Alto Douro, Vila Real, Portugal;* ^6^ *Departamento de Neurologia, Centro Hospitalar Universitário de Santo António, Porto, Portugal;* ^7^ *Serviço de Neurologia, Hospital de Santa Maria, Centro Hospitalar de Lisboa Norte, Lisboa, Portugal;* ^8^ *Hospital Dona Estefânia, Centro Hospitalar Universitário de Lisboa Central, Lisboa, Portugal;* ^9^ *Centro Materno Infantil do Norte, Centro Hospitalar Universitário de Santo António, Porto, Portugal;* ^10^ *Departmento de Neurologia, Hospital Santo António dos Capuchos, Centro Hospitalar de Lisboa Central, Lisboa, Portugal;* ^11^*Serviço de Genética Médica, Centro Hospitalar Universitário de São João/Faculdade de Medicina da Universidade do Porto, Portugal;* ^12^ *Departmento de Neurologia, Centro Hospitalar Universitário de São João/Faculdade de Medicina da Universidade do Porto, Portugal;* ^13^ *Unidade de Neuropediatria, Departamento de Pediatria, Hospital de Santa Maria, Centro Hospitalar e Universitário de Lisboa Norte, Lisboa, Portugal;* ^14^ *ICBAS – Instituto de Ciências Biomédicas Abel Salazar, Universidade do Porto, Porto, Portugal;* ^*^ *Contributed equally*

**Introduction:** Dystonia is a movement disorder characterized by involuntary muscle contractions and abnormal posturing, which may present isolated or with additional signs. Over the last decade, whole-exome sequencing (WES) was instrumental to identify several new genetic causes of dystonia.

Our aim was to determine the diagnostic yield of a WES-based multigene panel for dystonia and characterize the mutational spectrum in this cohort.

**Methodology:** We reviewed our laboratory diagnostic database for patients (n=292) sequentially tested (2016-2023, august) with a WES-based multigene panel for dystonia.

**Results:** A molecular diagnosis of dystonia (clearly pathogenic or likely pathogenic variants) was established in 9% (n=25) of the patients. Approximately 51% (n=150) had an uncertain result, due to variants of unknown significance (VUS) while 40% (n=117) had no variants reported.

Six patients carried variants in PRKRA or SLC2A1 genes (n=3 each), and six had variants in GNAL, PRRT2 or TOR1A (n=2 each); eleven patients showed variants in ADCY5, ATM, CLCN1, GCH1, IFIH1, PANK2, PRKCG, SGCE, TH, THAP1 or YY1 genes (n=1 each); two additional patients carried large deletions (in chromosomes 16 and 18) affecting dystonia-related genes.

From the 267 patients without a conclusive molecular diagnosis, 44 were reanalysed with a larger multigene panel (clinical exome). A subsequent molecular diagnosis was achieved in 2 patients (2 KMT2B variants). After re-analysis, only 32 patients had VUS described, and no variants were reported in 10.

**Discussion:** A WES-based multigene panel allowed the molecular confirmation of 17 patients with (isolated or combined) dystonia and 8 with differential diagnoses. Several disease-causing variants, including CNVs, were identified. Since KMT2B was associated with dystonia only after 2016, it was not included in initial versions of this panel; therefore, KMT2B variants were identified only in reanalysis. The application of these WES-based multigene panels reduces time to diagnosis and enables more accurate molecular diagnosis for patients with hereditary dystonia.

#### P67 - RECLASSIFICATION OF BRCA1/2 VARIANTS PREVIOUSLY CLASSIFIED AS VUS (ACMG-AMP GUIDELINES) WITH GENE-SPECIFIC GUIDELINES FROM CLINGEN ENIGMA AND CANVIG-UK

Pedro Rodrigues^1,2^, Patrícia Theisen^1^, João Gonçalves^1,2^

^1^ *Human Genetics Department, National Institute of Health Dr. Ricardo Jorge, Lisbon, Portugal.* ^2^ *Center for Toxicogenomics and Human Health, Nova Medical School, Lisbon, Portugal.*

In recent years, the number of BRCA1/2 germline variants associated with hereditary breast/ovarian cancer syndrome (HBOC), classified as variants of uncertain significance (VUS) according to ACMG-AMP guidelines (ACMGg) has been increasing. Reclassification of VUS as (likely) benign or (likely) pathogenic is crucial for maximizing diagnostic yield and appropriately managing HBOC patients. Recently, specific guidelines to improve classification of BRCA1/2 variants were independently developed by ClinGen ENIGMA1 (CG-Eg) and CanVIG-UK2 (CV-UKg).

Main goals: i) independently reclassify BRCA1/2 variants previously classified as VUS (ACMGg) with the new guidelines (CG-Eg and CV-UKg); ii) compare the results between the different guidelines iii) evaluate the potential clinical impact of this reclassification.

BRCA1/2 germline variants identified in patients with suspected HBOC and previously classified as VUS (8 missense, 5 intronic) were independently reclassified according to CG-Eg and CV-UKg. Variant assessment included: query of clinical/population databases and use of VEP, Alamut, VarSome and Franklin-Genoox.

VUS reclassification (using CG-Eg versus CV-UKg) was in agreement for 10 variants (2 VUS, 6 likely benign (LB) and 2 benign (B)). The remaining 3 VUS were reclassified as LB with CG-Eg and kept as VUS with CV-UK. Application of specific guidelines reduced the number of VUS from 10 to 2 (CG-Eg) or to 5 (CV-UKg).

The main difference between CG-Eg and CV-UKg is related with the downgrading strength of PM2 and the upgrading strength of BP1 criteria (in CG-Eg) for missense variants present outside clinically important functional domains and without splicing impact. The difference in BP1 strength has a major impact, making CG-Eg more stringent and reducing the number of VUS. The use of different guidelines, even if gene-specific, can lead to dissimilar classifications, a general consensus leading to a unique international guideline will be useful.

1- https://cspec.genome.network/cspec/ui/svi/doc/GN092;

2- https://www.cangene-canvaruk.org/gene-specific-recommendations. Support: FCT/MCTES, ToxOmics and Human Health (UIDB/00009/2020). GenomePT(POCI-01-0145-FEDER-022184).

#### P68 - APPLICATION OF THE ABC CLASSIFICATION SYSTEM IN A CLINICAL CASE OF MARBACH-SCHAAF SYNDROME

Isabel Alonso^1^, Jorge D da Silva^2,3^, Sílvia Magalhães^1^, Maria L de Almeida^1^, Natália Tkachenko^2,3^, Ana R Soares^1,2,3^

^1^ *Medical Genetics Unit, Hospital Pediátrico, Centro Hospitalar e Universitário de Coimbra, EPE;* ^2^ *Prenatal Diagnosis Unit, Maternidade Bissaya Barreto – Centro Hospitalar e Universitário de Coimbra, EPE;* ^3^*Pathological Anatomy Unit, Centro Hospitalar e Universitário de Coimbra, EPE*

**Introduction:** ABC system is a recently described model for genetic variant classification that aims to complement the more commonly used American Collage of Medical Genetics (ACMG) guidelines. This model seems to bring advantages mainly for classification of variants of unknown significance (VUS), copy number variants, hypomorphic alleles, extensive homozygosity and regulatory changes. By separating functional and clinical consequences of a variant, and by reinforcing the importance of proper genotype-phenotype match, this systems aims to give a better guide to variant significance. G. Houge et al propose the application of the ABC system to ACMG or Sherloc classifications VUSs.

The authors aimed to classify a VUS using ABC system in a rare case of suspected Marbach-Schaaf syndrome, in order to understand if this system could clarify this variant role in this patient phenotype.

**Methodology:** Application of the ABC system classification in a VUS case, after full clinical and laboratory characterization.

**Results:** A 7 years-old boy was observed in Medical Genetics consultation presenting with severe intellectual disability (ID), autism spectrum disorder, auto and hetero aggressiveness, facial dysmorphisms, macrosomia and macrocephaly. The NGS panel for ID genes revealed the variant c.380C>T; p.(Ala127Val) in heterozygosity, in PRKAR1B gene, that occurred de novo. Even so, this variant continued to be classified as VUS, according to ACMG. This gene has been associated with Marbach-Schaaf syndrome, which was a clinically compatible phenotype. After ABC system application (Step A – Functional grading 3, Step B – Clinical grading 1, Step C – Combined 3 + 1=E), the variant was classified as “Genetic variant of potential interest”. The group that described the syndrome agreed that this could be our diagnosis.

**Discussion:** With this case, the authors wish to stress not only the need for applying, more than different, complementary systems for variants classification, but also the importance of clinical and laboratory collaboration in these challenging genetic cases. The ABC system can illustrate the advantage of this collaboration as both parties count for the final classification. For the patient himself and the family, this small change may help to understand “why?” as well as to give proper and specific genetic counselling.

**References:**

- Houge G., et al, Stepwise ABC system for classification of any type of genetic variant, EJHG, 2021

- Marbach et al, Variants in PRKAR1B cause a neurodevelopmental disorder with autism spectrum disorder, apraxia, and insensitivity to pain, GIM, 2021

- Marbach et al, Phenotypic characterization of seven individuals with Marbach–Schaaf neurodevelopmental syndrome, AJMG Pt A, 2022

#### P69 - TUMOURS WITH CDH1 GENOMIC LOSS ASSOCIATE WITH BEST OUTCOME IN MICE AND MEN

Nelson Martins^1,2,3^, Rita Barbosa-Matos^1,2,4^, Celina São José^1,2,5^, Alexandre Dias^1,2,4^, Irene Gullo^1,2,6^, Silvana Lobo^1,2,3^, Francisco Moreira^6^, Ana Luisa Amaral^1^, Mafalda Santos^1,2^, Marta Ferreira^1,2,7^, Kate Eason^8^, Raquel Manzano-Garcia^8,9^, Oscar M. Rueda^9^, Suet-Feung Chin^8^, Carla Oliveira^1,2,3^

^1^ *i3S – Instituto de Investigação e Inovação em Saúde, Porto, Portugal;* ^2^ *IPATIMUP – Institute of Molecular Pathology and Immunology of the University of Porto, Portugal;* ^3^ *Faculty of Medicine, University of Porto, Portugal;* ^4^ *nstitute of Biomedical Sciences Abel Salazar, University of Porto, Portugal;* ^5^ *Max Planck Institute for Molecular Genetics, RG Development & Disease, Berlin, Germany.* ^6^ *Department of Pathology, Centro Hospitalar Universitário de São João, 4200-319 Porto, Portugal;* ^7^ *Department Computer Science Faculty of Science, University of Porto, Porto, Portugal.* ^8^ *CRUK Cambridge Institute, University of Cambridge, Cambridge, UK;* ^9^ *MRC Biostatistics Unit, University of Cambridge, Cambridge.*

**Introduction:** Breast cancer is the most incidence cancer worldwide and the leading cause of cancer-associated deaths in females. Breast cancer’s copy-number landscape and transcriptomic profile can define different IntClusters with distinguished molecular drivers and survival probabilities.

CDH1 is a tumour suppressor gene, with a proven role in gastric and breast carcinogenesis. Previous results indicate CDH1 intron 2 with regulatory features. Using in vivo experiments and in silico analysis of METABRIC cohort, we aim to explore the putative role of CDH1 intron 2 regulatory regions in breast cancer progression and prognosis.

**Methodology:** We evaluated tumour burden in lungs from mice injected with CRIPSR-Cas9 clones harbouring distinct structural variants causing CDH1 impairment: one exonic and one intronic. Furthermore, we divided METABRIC patients into Homozygous deletions (HOMD), Heterozygous Deletion (HETD) and Amplification (AMP) - iCREs Study Groups - depending on the CDH1 intron 2 copy number and compared clinical-pathological and survival features.

**Results:** Results demonstrate that cancer cells with CDH1 intron 2 impairment present lower tumour burden in mice lung’s, suggesting a role for these regions in the metastasis process. Each iCREs Study Group presented distinguished clinical features, such as ER and HER2 status, tumour grade and NPI classifications. Evaluation of the tumour transcriptome of AMP and HOMD groups showed a closer profile with intronic edited clone, than with exonic edited clone. Kaplan-Meier survival analysis also suggests breast tumours with CDH1 intron loss displaying a better prognosis.

**Discussion:** These findings support the hypothesis that CDH1 intronic regulatory elements play a role in lung colonization in mice and modulate breast cancer prognosis in humans.

**Acknowledgements:** Solve-RD fellowship (H2020-SC1-2017); Erasmus+ 2020 Credit Mobility (2020-1-PT01-KA103-077702); 3DChroMe (PTDC/BTM-TEC/30164/2017; POCI-01-0145-FE

#### P70 - IMPORTANCE OF REPEATING AN INCONCLUSIVE cfDNA RESULT – A THREE-YEAR RETROSPECTIVE STUDY

Maria Lopes-de-Almeida^1,2^, Ana R Soares^1,3,4^, Sílvia Magalhães^1^, Isabel Alonso^1^

^1^ *Genetyca-ICM, Atrys, Portugal.* ^2^ *Serviço de Genética Médica, Hospital de Braga EPE, Braga, Portugal;* ^3^ *Serviço de Genética Médica, Centro de Genética Médica Dr. Jacinto Magalhães, Centro Hospitalar Universitário de Santo António, Porto, Portugal.* ^4^ *Unidade Multidisciplinar de Investigação Biomédica, Instituto de Ciências Biomédicas Abel Salazar, Universidade do Porto, Portugal.*

**Introduction:** Non-invasive prenatal testing (NIPT) is a very accurate screening test that uses cell-free DNA (cfDNA) in maternal blood for specific chromosomal disorders in the fetus. Although in most of the cases a conclusive, positive or negative result, is obtained, there is a small percentage of cases that are inconclusive due to technical issues, such as low fetal fraction or insufficient sequencing data. In the literature, the proportion of inconclusive results ranges between 1% and 8%, mainly due to the applied technology used and the gestational age at blood collection. An inconclusive result has been associated with an increased risk for aneuploidies.

**Methodology:** We performed a retrospective study between 1st september 2020 and 31st august 2023 of all pregnancies with an inconclusive cfDNA from our laboratory.

**Results:** From a total 8025 cases, 256 (3.2%) resulted in an inconclusive result. Three cases were not repeated due to pregnant woman option or early fetal loss. For 253 cases a new blood collection was performed and resulted in 231 (91.3%) low-risk results (5 of them non conclusive for sex chromosome aneuploidies), 12 (4,7%) were high-risk results and 10 (4%) remained inconclusive after the second test.

**Discussion:** We describe our experience over the past three years regarding inconclusive NIPT results. This study shows the importance of new blood collection after an inconclusive cfDNA result, reducing the number of cases in which an invasive test is necessary.

**References**

1. Van der Meij KRM, Sistermans EA, Macville MVE, et al. TRIDENT‐2: national implementation of genome‐wide non‐invasive prenatal testing as a first‐tier screening test in The Netherlands. Am J Hum Genet. 2019;105(6):1091‐1101. 10.1016/j.ajhg.2019.10.005

2. Gil MM, Quezada MS, Revello R, Akolekar R, Nicolaides KH. Analysis of cell‐free DNA in maternal blood in screening for fetal aneuploidies: updated meta‐analysis. Ultrasound Obstet Gynecol. 2015;45(3):249‐266. 10.1002/uog.14791

3. Yaron Y. The implications of non‐invasive prenatal testing failures: a review of an under‐discussed phenomenon. Prenat Diagn. 2016;36(5):391‐396. 10.1002/pd.4804

#### P71 - THE ROLE OF GENETIC COUNSELING IN PRE-SYMPTOMATIC GENETIC TESTING: LESSONS FROM FAMILIAL AMYLOIDOSIS POLYNEUROPATHY (FAP)

Ana Miguel Capela^1^, Carla Carmona^1,2^, Ana Rita Soares^1,2^, Célia Soares^1,2,3,4^, Isabel Serra Nunes^1,2^, Jorge Diogo Silva^1,2,5,6^, Maria Abreu^1^, Nataliya Tkachenko^1,2^, Ana Maria Fortuna^1,2^, Cláudia Falcão Reis^1,2,5,6^, co-first authors

^1^ *Serviço de Genética Médica, Centro de Genética Médica Jacinto Magalhães, Centro Hospitalar Universitário de Santo António, Porto, Portugal;* ^2^ *Unit for Multidisciplinary Research in Biomedicine, Instituto de Ciências Biomédicas Abel Salazar/Universidade do Porto, Porto, Portugal;* ^3^ *Departamento de Ciências Médicas, Universidade de Aveiro, Aveiro, Portugal;* ^4^ *i3S – Instituto de Investigação em Saúde, Universidade do Porto, Porto, Portugal;* ^5^ *Life and Health Sciences Research Institute (ICVS), University of Minho, Campus de Gualtar, Braga, Portugal;* ^6^ *ICVS/3B’s, PT Government Associated Laboratory, Braga/Guimarães, Portugal - Clinical Academic Center, Braga, Portugal*

**Introduction:** Pre-symptomatic genetic testing (PSGT) allows for identification of individuals at risk of developing hereditary conditions. Portuguese law (Law nº12/2005, article 9º) establishes that PSGT for adult-onset diseases is ordered by medical geneticists providing genetic counseling (GC), and, if there’s no effective treatment, after a mandatory psychological evaluation. GC offers individuals and families support to navigate the complexities of hereditary conditions. In an era of personalized medicine, early detection allows for targeted interventions and better patient care. We aim to highlight the critical role of GC in PSGT, with TTR-related Familial Amyloidosis Polyneuropathy (FAP), an autosomal dominant condition, as an example.

**Methodology:** Retrospective descriptive study. Review of clinical files to collect clinical, genetic, and social data from patients seen in our center from January 2015 to August 2023.

**Results:** 443 consultands were referred for PSGT, with the majority referred due to FAP family history (60%:144 females:124 males). In this group, 12 consultands opted out, one could not give informed consent due to intellectual disability, and three reported symptoms, expediting the process. Out of the 251 individuals that underwent testing, 157 had a negative result, and 93 were carriers for a pathogenic variant. Referral to Neurology consultation was provided when genetic testing was positive, and 83/95 were evaluated. On first Neurology assessment, 80 consultands were asymptomatic and 3 patients had mild unspecific symptoms. During routine follow-up, 9 patients developed symptoms. All symptomatic patients are currently under treatment with Tafamidis or Patiseran. Individuals and couples were informed about their reproductive options and given the possibility of further counselling before family planning. Within the reproductive options, four carriers opted for referral for Pre-implantation genetic testing (PGT).

**Discussion:** FAP was the most common referral given its prevalence and increasing treatment options. Not all consultands opted for testing, underlining the importance of GC in individual’s decision making. A medical evaluation is crucial to this process, allowing for urgent referral of symptomatic patients.

**Conclusions**: GC is an integral component of pre-symptomatic genetic testing, exemplified here by its role in FAP. GC empowers individuals to make informed choices about genetic testing, family planning, and personalized healthcare.

#### P72 - THE USE OF GENE-SPECIFIC CLASSIFICATION GUIDELINES VS ACMG 2015: MODY CASE STUDY

Paulo Dario^1,2,*^, Margarida Vaz^1,*^, Gisela Gaspar^1^, Mafalda Bourbon^1,2^

^1^ *Department of Health Promotion and Prevention of Noncommunicable Diseases, National Institute of Health Doutor Ricardo Jorge, 1649-016 Lisbon, Portugal;* ^2^*BioISI - BioSystems & Integrative Sciences Institute, Faculty of Sciences, University of Lisbon, 1749-016 Lisbon, Portugal.* ^*^ *These authors were equal contributors to this study.*

**Introduction:** MODY is a dominant monogenic form of diabetes caused by the presence of a pathogenic variant in one of the 14 genes identified to date as associated with this disease. The process of variant classification using the 2015 ACMG classification guidelines can be complex and time-consuming, and the data needed to assess pathogenicity is difficult to interpret and also generic to apply to a specific disease. The Monogenic Diabetes (MDEP) Variant Classification Expert Panel (VCEP) has developed specific guidelines for the classification of MODY variants and regularly reviews the classification of variants submitted to ClinVar. For this, having comprehensive MODY variant database with co-segregation information, variant prevalence, phenotypic and population data, and in silico evidence is essential for accurate classification as well as can be helpful for labs the sharing of their data. In near future, with routine use of NGS, there will be an increase in the number of rarer variants, which will have even less available information and for that reason will be more difficult to classify. Therefore, following the same guidelines in variant classification and frequently check ClinVar to detect alterations in classification of variants are good practices that laboratories should follow.

Our aim here is to present how variants previously classified as VUS using 2015 ACMG guidelines have been able to arrive at a definitive classification using the specific MDEP rules.

**Methods:** In this study, we compared the classification of two variants found in patients from the Portuguese MODY study, initially considered as VUS using the ACMG 2015 guidelines and later reclassified applying MDEP guidelines, but also confirmed at ClinVar.

**Results:** Applying 2015 ACMG guidelines, both variants NM_000545.8(HNF1A): c.599G>A(p.Arg200Gln) and NM_000162.5(GCK):c.757G>A(p.Val253Ile) were classified as VUS. The first variant was later reclassified as pathogenic using the MDEP guidelines, and it was given a revised status in ClinVar by the MDEP expert panel. The second variant remained as VUS according to MDEP.

**Conclusions:** Using VCEP specific guidelines instead of generic ACMG ones may result in a more accurate and definitive classification because these are disease/gene specific. As with other VCEP guidelines, MDEP includes decisions on how to procced for large deletions, which were not contemplated in the generic ACMG procedure.

#### P73 - CUMULUS CELL DAMAGE INDIRECTLY HELPS TO PREDICT OOCYTE QUALITY IN INFERTILE FEMALES UNDERGOING ICSI

Bárbara Rodrigues^1,2,3,4,5^, Vanessa Sousa^1,2,3,4,5^, Filipa Esteves^3,4,5^, Joana Pires^3,4,5^, Daniela Sousa^6^, Raquel Brandão^6^, Carla Leal^6^, Rosário Santos^1,2,3^, Emídio Vale-Fernandes^2,3,6^, António J. A. Nogueira^7^, Solange Costa^3,4,5^, Paula Jorge^1,2,3^

^1^ *Serviço de Genética Laboratorial, Clínica de Genética e Patologia, Centro Hospitalar Universitário de Santo António (CHUdSA), Porto, Portugal;* ^2^ *UMIB - Unit for Multidisciplinary Research in Biomedicine, ICBAS - School of Medicine and Biomedical Sciences, University of Porto, Porto, Portugal;* ^3^ *ITR - Laboratory for Integrative and Translational Research in Population Health, Porto, Portugal;* ^4^ *Environmental Health Dept, National Institute of Health, Porto, Portugal EPIUnit;* ^5^ *Instituto de Saúde Pública da Universidade do Porto, Portugal;* ^6^ *Centre for Medically Assisted Procreation/ Public Gamete Bank, Centro Materno-Infantil do Norte Dr. Albino Aroso (CMIN), Centro Hospitalar Universitário de Santo António (CHUdSA), Porto, Portugal;* ^7^ *CESAM - Center for Environmental and Marine Studies, Department of Biology, University of Aveiro, Aveiro, Portugal.*

**Introduction:** Intracytoplasmic sperm injection (ICSI) is currently used in clinical practice for couples with fertility issues. Some studies have shown an association between male reproductive ability and sperm DNA damage levels, assessed by comet assay. However, little is known regarding this endpoint and female fertility, mostly due to tissue accessibility. To overcome this, we used cumulus cells (CC) to analyze DNA damage in search of correlation with clinical parameters evaluated in the context of infertility.

**Methodology:** DNA damage was assessed via comet assay, in two different tissues, blood and CC, from females undergoing ICSI: 22 potentially fertile and 35 infertile. DNA damage levels (%TDNA) were compared between the two groups (fertile vs infertile), and correlated, within each group, with hormone levels, stimulation days, number of cumulus-oocyte complexes (COCs) retrieved, and oocytes injected. All analysis were performed using SigmaPlot version 14.0 (Systat Software®Inc., Chicago, IL, United States).

**Results:** No significant differences were found in %TDNA levels between the 2 groups. However, the DNA damage observed in CC was notably increased in the infertile females when compared to the potentially fertile, although it did not reach statistical significance. Interestingly, %TDNA in CC was significantly correlated with the number of oocytes injected, in both groups. This finding was not observed in the blood. Moreover, the difference between number of COCs retrieved and oocytes injected was significantly higher in the infertile females group and showed a correlation with the damage observed in CC.

**Discussion:** Our results established a correlation between DNA damage in CC and oocyte quality. CC support and nurture oocytes during development, but DNA damage in CC can predict a reduced oocyte quality and availability for injection. This finding underscores the importance of CC in oocyte development and emphasizes the need to consider tissue-specific effects in DNA damage studies, particularly those related to fertility and reproductive health. Nevertheless, further studies are needed to confirm our results.

### CLINICAL CASES

#### P74 - UNRAVELLING THE C.295C>T VARIANT IN FANCA: EXAMINING ITS UBIQUITY AND DIVERSE PRENATAL MANIFESTATIONS AMONG THE ROMANI IBERIAN POPULATION

Diogo Fernandes da Rocha, MD^1^, Carla Ramalho, MD, PhD^2,3,4^, Renata d’ Oliveira, MD^1^, Ana Grangeia, MD, PhD^1,2^

^1^ *Serviço de Genética Médica, Centro Hospitalar Universitário de São João, Porto, Portugal;* ^2^ *Faculdade de Medicina da Universidade do Porto, Porto, Portugal;* ^3^ *Centro de diagnóstico pré-natal, Serviço de Obstetrícia, Centro Hospitalar Universitário de São João, Porto, Portugal;* ^4^ *i3s – Instituto de Investigação e Inovação em Saúde, Porto, Portugal*

**Introduction:** Fanconi anemia (FA) is a rare genetic disorder characterized by bone marrow failure, congenital abnormalities (such as skeletal malformation of the limbs), and an elevated risk of cancer.

**Methodology:** Here, we describe three different prenatal FA manifestations tied to the same variant. The first one, a first-time pregnant Romani adolescent that showed gastric dilation in a morphological ultrasound and a double bubble sign at 25 gestational weeks, suggestive of a possible high intestinal obstruction (duodeno-jejunal stenosis/atresia). Its neonatal biochemical screening revealed elevated immunoreactive trypsinogen levels in two consecutive samples. This newborn eventually died on the 35th day of life due to multisystemic failure, including hypoxic-ischemic encephalopathy, acute kidney injury, and non-hepatic cholestasis. The second one, a 41-year-old Romani woman with a G10P9 pregnancy who had a radial axis anomaly in an ultrasound performed at 16 gestational weeks. The family history was remarkable, notably a 23-year-old daughter with bilateral thumb agenesis, and two monozygotic twin daughters, one with bilateral thumb agenesis and deviation of the right wrist, and the other with varying thumb sizes. The third one, is a three-year-old Romani boy with prenatal diagnosis of single pelvic kidney, cryptorchidism and IUGR, and postnatal diagnosis of oesophageal atresia and tracheoesophageal fistula with bilateral radial agenesis.

**Results:** In the first patient, it was requested the most frequent pathogenic variants for cystic fibrosis (negative) and NGS panel for cholestasis (only Gilbert’s Syndrome detected). Upon revaluation of clinical exome sequencing data, it was identified the homozygous pathogenic variant c.295C>T, p.(Gln99*) in the FANCA gene. In the second one, it was performed genetic targeted analysis for the referred FANCA variant, confirming the homozygous state in the affected foetus. For the third one, cytogenetic testing showed heightened chromosome breakage with DEB, and targeted genetic testing confirmed the homozygosity for the same FANCA variant.

**Discussion:** Previous articles have found that the variant c.295C>T is associated with a founder effect in the Romani Iberian population (PMID: 15522956). These cases underscore this variant’s phenotypic variability within this homogeneous population. Declaration of Interests: Nothing to declare.

#### P75 - CHARACTERIZATION OF NFIB-ASSOCIATED PHENOTYPES – AN INHERITED NFIB PARTIAL DELETION

Raquel Rodrigues^1^, Sílvia Serafim^2^, Ana Sousa^1^, Ana Berta Sousa^1^

^1^ *Serviço de Genética Médica, Departamento de Pediatria, Hospital de Santa Maria, CHULN, Lisboa, Portugal;* ^2^ *Departamento de Genética Humana, Instituto Nacional de Saúde Doutor Ricardo Jorge, Lisboa, Portugal*

**Introduction:** Microdeletions involving NFIB are rare. In 2018, haploinsufficiency was associated with the autosomal dominant disorder ‘Macrocephaly, acquired, with impaired intellectual development (MACID)’ (#618286), characterized by intellectual disability (ID), macrocephaly, motor delay, hypotonia, behavioral abnormalities, and structural brain anomalies. We report a maternally inherited NFIB deletion, aiming to help delineate the molecular and phenotypic spectrum of this rare syndrome.

**Methods:** The proband is a 9yo girl, whose mother was referred to Genetics in pregnancy due to learning difficulties and facial dysmorphism. She was followed up from birth, and evolved with mild developmental delay, attention deficit hyperactivity disorder, macrocephaly, and facial features overlapping her mother, namely long face with high forehead, thin eyebrows, narrow nasal bridge, low columella, thin upper lip, and jutting chin.

DNA samples were analyzed by aCGH.

**Results:** aCGH in the proband revealed a 150.68kb deletion in 9p23p22.3, encompassing 5’UTR and first 2 exons of NFIB gene. Subsequently, aCGH data from her mother, dating back to the pregnancy, was reevaluated, and the same deletion, previously classified as VOUS before MACID description, was reclassified as pathogenic.

**Discussion:** Members of the NFI family are pivotal in normal development of several organs, including the brain. Deletions and sequence variants in NFIA and NFIX are associated with ID and structural brain anomalies. Recently, it was shown that MACID is caused by NFIB haploinsufficiency. Thus far only 5 different deletions harboring exclusively NFIB were reported. We describe 2 additional cases with the smallest deletion so far.

It was hypothesized that deletions including additional genes correlated with more pronounced dysmorphism. The clinical features observed in our patients are similar to those observed in individuals with SNVs, and in those with deletions encompassing either only the whole NFIB gene or extending to neighboring genes, which challenges this notion.

This case shows the importance of periodical review of patient data.

#### P76 - RARE 47,XXY/46,XX MOSAICISM IN A CHILD WITH AN OVOTESTICULAR DISORDER OF SEXUAL DEVELOPMENT (OT-DSD)

Nayara D. Oliveira^1^, Ana C. Alves^1^, Barbara S. Marques^1^, Laurentino R. Simão^1^, Ricardo C. Peliano^1^, Mónica I. Viegas^1^, Humberto Vassal^2^, Raquel G. Silva^3^, Filomena M. Brito^1^, Hildeberto O. Correia^1^

^1^ *Unidade de Citogenética, Departamento de Genética Humana, Instituto Nacional de Saúde Doutor Ricardo Jorge, Lisboa;* ^2^ *Serviço de Pediatria, Hospital Portimão, Centro Hospitalar Universitário Algarve, Portimão;* ^3^ *Serviço de Genética Médica, Departamento de Pediatria, Hospital de Santa Maria, Centro Hospitalar Universitário Lisboa Norte, Centro Académico de Medicina de Lisboa, Lisboa.*

Sex chromosomal abnormalities can disrupt the processes of sex determination and subsequent sex differentiation, potentially leading to disorders of sexual development (DSD). One such rare condition is mosaicism with a 46,XX/47,XXY chromosomal pattern, which presents variable phenotypes and clinical manifestations, including ovotesticular DSD (OT-DSD). OT-DSD is a rare disorder of sexual differentiation, accounting for 3 to 10% of all DSD, and is characterized by the presence of both ovarian and testicular tissues in the same individual.

We present the case of a child referred to karyotype analysis due to ambiguous genitalia (AG) observed at birth, later identified with OT-DSD. The child was born after a closely monitored pregnancy without complications, and both parents were healthy individuals with no significant family history.

Cytogenetic analysis using GTL banding was performed on peripheral blood, revealing a mosaic karyotype with two cell lines: 46,XX (72%) and 47,XXY (28%). The presence of the SRY gene, was confirmed by Fluorescence in situ Hybridization (FISH). Buccal swab sampling, using X and Y centromere FISH probes also reasserted this mosaicism on interphase nuclei.

At 2 months of age, pelvic ultrasound revealed lateral ovotestis (right)/ovarian structure (left) and a uterus with the expected dimensions and morphology. At 18 months of age, the patient underwent surgical removal of the ovotestis, and chromosomal analysis was performed, once again revealing the same mosaic pattern but with a different proportion of cell lines 46,XX (34%) and 47,XXY (66%).

A child presenting OT-DSD poses a complex challenge for a multidisciplinary team comprising specialized clinicians such as paediatricians, endocrinologists, surgeons, psychologists, and geneticists, along with the support of a genetic laboratory. Following extensive clinical investigations, karyotype analysis and additional genetic tests are indispensable for unravelling the genetic aetiology of this disorder. They enhance our capacity to predict the patient’s phenotype and play a crucial role in making informed medical decisions. Regular follow-up is essential to ensure the optimal health and quality of life for individuals affected by OT-DSD.

**Conflict of interest:** None declared.

**Acknowledgments**

We thank the patient and the family for the study and all professionals involved.

#### P77 - AP1B1-RELATED AUTOSSOMAL RECESSIVE KERATITIS-ICHTHYOSIS-DEAFNESS SYNDROME (KIDAR)

Rosário Silveira-Santos, Patrícia Dias, Ana Sousa, Ana Berta Sousa

Serviço de Genética Médica, Departamento de Pediatria, Hospital de Santa Maria, CHULN, Lisboa, Portugal

**Introduction:** The widespread use of arrayCGH technology has led to identification of several patients with combined microdeletion and microduplication syndromes.

6p25 microdeletion syndrome is a rare neurodevelopmental disorder characterized by a wide spectrum of congenital abnormalities. Ocular anomalies are frequently seen and have been linked to FOXC1 haploinsufficiency (MIM*601090).

17q12 microduplication syndrome (MIM#614526) has a variable phenotype and incomplete penetrance for developmental delay (DD)/intellectual disability (ID), such that it is often inherited from an apparently unaffected parent.

We present the first report of a patient with copy number changes involving both these syndromic regions.

**Methods:** The patient is a 20-year-old man, the second child of non-consanguineous parents. His father had learning disability and mild proportionate short stature. He was born with congenital glaucoma and aniridia and exhibited central corneal opacity on the left eye. He presented short stature and had no dysmorphic features. He graduated high school with a professional diploma, with educational support but no learning difficulties. His psychological assessment showed no cognitive deficit.

**Results:** Microarray analysis identified a 1.19 Mb interstitial deletion in 6p25.3p25.2 involving three genes, including FOXC1, as well as a 1.39 Mb copy gain in chromosome 17q12 encompassing 14 OMIM genes, including LHX1 and HNF1B. FISH studies revealed that the deletion was de novo and the duplication was inherited in tandem from his father.

**Discussion:** To date, only a few cases of small 6p25 deletions overlapping our case have been described in the literature. This deletion explains the patient´s eye phenotype as FOXC1 gene is associated with anterior segment developmental anomalies of the eye.

17q12 duplication can explain the family’s neurodevelopmental phenotype. The absence of DD/ID in our patient may be related to the incomplete penetrance of these features in both syndromes and a smaller number of genes involved in comparison to other cases. Targeted educational intervention has probably contributed to the successful academic outcome.

#### P78 - REPORT OF A RARE 3q29 INTERSTITIAL MICRODELETION: PRENATAL DIAGNOSIS AND POSTNATAL FOLLOW-UP

Laurentino Simão^1^, Sónia Pedro^1^, Bárbara Marques^1^, Sílvia Serafim^1^, Cristina Ferreira^1^, Ana Tarelho^1^, Filomena Brito^1^, Marisa Silva^1^, Cristina Alves^1^, Mónica Viegas^1^, Ana Paula Silva^2^, Márcia Rodrigues^3^, Ângela Ferreira^2^, Hildeberto Correia^1^

^1^*Unidade de Citogenética, Departamento de Genética Humana, Instituto Nacional de Saúde Doutor Ricardo Jorge, I.P., Lisboa*, ^2^ *Centro de Diagnóstico Pré-Natal, Centro Hospitalar Universitário do Algarve, Hospital de Faro, Faro*, ^3^ *Serviço de Genética Médica, Departamento de Pediatria, Hospital de Santa Maria, Centro Hospitalar Universitário Lisboa Norte, Lisboa*

Distal interstitial deletions in the 3q29 region are rare. The characterization of new prenatal diagnosis (PND) cases and their follow-up may add knowledge about the affected region.

A 21-year-old woman was referred for PND at 20 weeks of gestation due to fetal increased nuchal translucency, cystic renal dysplasia and hyperechogenic focus on the left ventricle. A microarray (CMA) study was performed using the CytoScan 750K (Thermo Fischer®), after a normal result in the rapid aneuploidies diagnosis by QF-PCR (Devyser®).

CMA identified a ~594 Kb interstitial deletion at 3q29 - arr[GRCh37] 3q29 (196190768-196784544)x1, in a XX fetus; a similar deletion was identified in the mother. This is located into the described 3q29 recurrent deletion syndrome region that spans 1.6 Mb. The pregnancy continued after genetic counseling, and the term newborn presented an Apgar score of 10/10.

The identified microdeletion encompasses several genes, including 3 OMIM morbid genes, RNF168, NRROS, and CEP19, associated with autosomal recessive heredity. It is not possible to detect prenatal characteristics associated with them. The deletion may be considered of uncertain clinical significance, possibly pathogenic.

Regarding prenatal age, two reports described deletions partially overlapping with the present one: a case of a de novo deletion and another with maternal inheritance. Both presented intrauterine growth restriction and the first had ventricular septal defect (VSD).

The 3q29 recurrent microdeletions detected in PND are also rare, presenting reduced birth weight, with VSD occurring only in isolated cases. The pregnancy was uneventful in most cases.

In the present case, normal cardiac evaluation and psychomotor development were found in the neonatal and postnatal periods; there were no facial dysmorphisms or morphological changes. Only a small left kidney and the presence of cortical cystic formations were observed and are being monitored by pediatric nephrology. At the 12-month appointment, the child presented normal psychomotor development.

Amongst the cases reported with microdeletions in 3q29, the presence and severity of symptoms are variable, and the penetrance is incomplete. Even in inherited deletions phenotypic variability has been reported, and thus, inheritance may not be the most relevant factor to predict the phenotype.

The case presented brings an addition to the rare data in prenatal age and reinforces the knowledge about distal interstitial microdeletions in the 3q29 region in prenatal age and in the neonatal and postnatal periods.

#### P79 - FAMILIAL ADENOMATOUS POLYPOSIS (FAP) - PREVALENCE OF PATHOGENIC VARIANTS USING A NEW MULTIGENE PANEL: RETROSPECTIVE REVIEW IN A SYNLAB LABORATORY CENTER

Ana Correia^1^, Lisandra Castro^1^, Márcia Cardoso^1^, Ariana Conceição^1^, Natália Salgueiro^1^, Michael Freitas^1^, Inês Ribas^1^, João Mata^1^, Natalya Tkachenko^2^, Cláudia Falcão Reis^2^, Mariana Soeiro e Sá^3^, Oana Moldovan^3^, Maria Lopes de Almeida^4^, Alexandra Gonçalves-Rocha^4^, Ana Sousa^3^, Sara Ribeiro^5,6^, Sofia Maia^5,6,7^, Margarida R. Lima^1^

^1^ *Unidade de Genética Molecular e Genómica-SynlabHealth Genética Médica, Porto;* ^2^ *Serviço de Genética-Centro Hospitalar do Porto;* ^3^ *Serviço de Genética-Centro Hospitalar de Lisboa Norte;* ^4^ *Unidade de Genética Médica-Hospital de Braga;* ^5^ *Serviço de Genética Médica, Hospital Pediátrico, Centro Hospitalar e Universitário de Coimbra Portugal;* ^6^ *Centro Académico Clínico de Coimbra, Coimbra, Portugal;* ^7^ *Clínica Universitária de Genética, Faculdade de Medicina da Universidade de Coimbra, Portugal*

**Introduction:** Colorectal carcinoma (CRC) is one of the most common neoplasms worldwide, and there are several genetic syndromes that predispose to CRC. Some of these syndromes are also characterized by the development of many (tens to thousands) polyps in the rectum and colon. Genetic analysis by next generation sequencing has become increasingly available and cheaper, and we, therefore, designed a new multigene panel, which includes several genes associated with hereditary polyposis syndromes, allowing for a more precise diagnosis.

**Methods:** To determine the pathogenic/likely pathogenic (P/LP) variants prevalence in intestinal polyposis cases, we reviewed the results of 226 cases tested at our center. All samples were tested by a multigene panel, that included the following genes: APC, BMPR1A, MSH3, MUTYH, NTHL1, POLE, POLD1, PTEN, RNF43, SMAD4 and STK11. We divided the cases in two subgroups: patients with personal and family history of polyposis and patients with polyposis, but no family history of polyposis. NGS was performed on Ilumina Platform, using the Twist Human Comprehensive Exome (Twist Bioscience).

**Results:** Our study revealed 23 P/LP variants in all analysed genes (23/226=10%). However, we noticed that in the subgroup of patients with personal and family history of polyposis (n=9) the prevalence is much higher (22%) than in the other subgroup (n=216; prevalence of 9%).

Most of the P/LP variants (82%) detected in these patients were in the APC and MUTYH genes. The remaining variants (18%) were detected in the NTHL1, POLE, PTEN and SMAD4 genes.

**Discussion:** This retrospective study demonstrated that APC and MUTYH genes continue to be the main genes associated with polyposis. Since in almost a fifth of the cases the underlying genetic cause was due to P/LP variants in other genes, the use of a polyposis multigene panel should be the standard testing approach. This approach will result in a higher diagnosis rate and more tailored surveillance strategy.

#### P80 - MUTATION PATTERN IN PORTUGUESE PATIENTS WITH HEREDITARY AND SPORADIC BREAST AND OVARIAN CANCER. EXPERIENCE OF A SYNLAB LABORATORY CENTER

Márcia Cardoso^1^, Lisandra Castro^1^, Ana Correia^1^, Ariana Conceição^1^, Natália Salgueiro^1^, Marcelo Dantas^1^, Sónia Barros^1^, Cristiana Ferreira^1^, Natalya Tkachenko^2^, Cláudia Falcão Reis^2^, Mariana Soeiro e Sá^3^, Oana Moldovan^3^, Maria Lopes de Almeida^4^, Alexandra Gonçalves-Rocha^4^, Sara Ribeiro^5,6^, Sofia Maia^5,6,7^, Ana Sousa^3^, Margarida R. Lima^1^

^1^ *Unidade de Genética Molecular e Genómica-SynlabHealth Genética Médica, Porto;* ^2^ *Serviço de Genética-Centro Hospitalar do Porto;* ^3^ *Serviço de Genética-Centro Hospitalar de Lisboa Norte;* ^4^ *Unidade de Genética Médica-Hospital de Braga;* ^5^ *Serviço de Genética Médica, Hospital Pediátrico, Centro Hospitalar e Universitário de Coimbra, Coimbra, Portugal;* ^6^ *Centro Académico Clínico de Coimbra, Coimbra, Portugal;* ^7^ *Clínica Universitária de Genética, Faculdade de Medicina da Universidade de Coimbra, Coimbra, Portugal*

**Introduction:** Next-generation sequencing (NGS) allows for a more cost-effective and quickly detection of pathogenic and likely pathogenic variants. Identification of these variants in breast and ovarian cancer allows for increased clinical surveillance, early detection and surgical decision and predicts the response to poly (ADP-ribose) polymerase (PARP) inhibitors.

**Methods:** To determine the pathogenic/likely pathogenic (P/LP) variants prevalence in HBOC and SBOC cases, we performed a retrospective review of 1038 samples received at the Synlab laboratory of Porto center between January 2022 and August 2023. All samples were tested by a multigene panel, that included the following genes: ATM, BARD1, BRCA1, BRCA2, BRIP1, CDH1, CHEK2, CTNNA1, EPCAM, MLH1, MSH2, MSH6, NBN, PALB2, PMS2, PTEN, RAD51C, RAD51D, STK11 and TP53. NGS was performed on Illumina Platform, using a custom Twist kit (Twist Bioscience).

**Results:** Our study revealed 85 P/LP variants in all analysed genes (85/1038=8%). However, we noticed that in the subgroup with breast or ovarian cancer and family history of HBOC (n=315) the prevalence is higher (11,1%), while in the subgroup with no family history (n=684) the prevalence is 7%. In the subgroup of healthy individuals with family history of breast and ovarian cancer (n=39) only 2.5% had P/LP variants. Within the P/LP variants, 46% of the variants were identified in BRCA1 and BRCA2. The non-BRCA1/2 genes variants represent 54% of the total P/LP variants detected.

**Discussion:** This retrospective review demonstrated the relevance of using a multigene panel as a standard approach for breast and ovarian cancer patients. It was also important to highlight the genetic heterogeneity found in these patients since the majority of P/LP variants were detected in non-BRCA1/2 genes.

#### P81 - ARTHROGRYPOSIS MULTIPLEX CONGENITA TYPE 6: PRENATAL DIAGNOSIS THROUGH THE POWER OF WHOLE EXOME SEQUENCING (WES)

Ariana Conceição^1^, Natalia Salgueiro^1^, Lisandra Castro^1^, Claudia Alves^2^, Marcelo Dantas^1^, João Mata^1^, Célia Mendes^1^, Elsa Garcia^1^, Joana Trindade^2^, Guilhermina Ladeira^3^, Margarida R. Lima^1^

^1^*Unidade de Genética Molecular e Genómica-SynlabHealth Genética Médica, Porto;* ^2^ *Unidade de Citogenética-SynlabHealth Genética Médica, Porto;* ^3^ *Unidade de Obstetrícia-Hospital de Cascais*

**Introduction:** Arthrogryposis multiplex congenita-6 (AMC6) is a severe genetic autosomal recessive disorder of skeletal muscle with onset of symptoms in utero. Pregnancies are usually complicated by polyhydramnios and reduced fetal movements. Affected individuals have congenital joint contractures, dysmorphic facial features, distal skeletal anomalies with clenched hands and clubfeet, and edema with fetal hydrops. Fetal demise or termination of pregnancy often occurs after ultrasound detection of abnormalities. Those that survive to birth have significant hypotonia with absent spontaneous movements, respiratory insufficiency, arthrogryposis, and multiple pterygia. Skeletal muscle is hypoplastic, immature, and underdeveloped, with nemaline rods, poorly developed sarcomeres, and poor cross-striation. Death in infancy usually occurs. We describe a case of an ongoing pregnancy where the ultrasound revealed a fetus with a very pronounced generalized subcutaneous edema (anasarca).

**Methods:** 24-year-old pregnant woman, amniocentesis at 23 weeks due to ultrasound that revealed a fetus with anasarca and poor movements. Microrray-CGH and WES analysis were performed. Bioinformatic analysis was focused on the phenotype related.

**Results/Discussion:** Our targeted phenotype-based analysis revealed 3 variants:

CNV´S analysis revealed a 1.7MB deletion on X chromosome (Xp22.31) which was also found in the array-CGH analysis. The recurrent region Xp22.31 (included STS gene) is associated with Ichthyosis, X-linked recessive (OMIM#308100), and confirms the carrier status of the female fetus.

Two heterozygous variants were found in NEB gene: c.21076C>T (p. Arg7026Ter) and c.22905 + 1delG. According to ACMG guidelines we classified these variants as Pathogenic. The clinical information provided allowed us to conclude that these variants were compatible with autosomal recessive Arthrogryposis multiplex congenita, 6 (OMIM#619334).

Prenatal diagnosis by WES becomes a widespread technique. Due to its sensitivity this technology has the potential to provide information that can lead to rare difficult diagnosis to be made on time, and guide future genetic counselling in families.

#### P82 - PRENATAL DIAGNOSIS OF A BECKWITH-WIEDEMANN SYNDROME CASE: CLINICAL SUSPICION AND MOLECULAR TEST AS A TOOL IN PRE-NATAL DIAGNOSIS

Elsa Garcia^1^, Marta Moreira^1^, Natália Salgueiro^1^, Lisandra Castro^1^, Ariana Conceição^1^, Cláudia Alves^2^, Joana Trindade^2^, Dulcina Lopes^3^, Margarida Reis Lima^1,2^

^1^*Unidade de Genética Molecular e Genómica-SynlabHealth, Genética Médica, Porto;* ^2^ *Unidade de Citogenética-SynlabHealth Genética Médica, Porto;* ^3^ *Hospital de Cascais, Unidade de Obstetrícia, Lisboa*

**Introduction:** Beckwith-Wiedemann syndrome (BWS; OMIM#130650) is an overgrowth disorder, characterized by macrosomia, congenital malformations and tumor predisposition, caused by dysregulation of gene expression in the imprinted 11p15.5 chromosomal region. Most of the affected BWS cases are diagnosed after birth and it is often difficult to diagnose it prenatally. The cluster of imprinted genes is divided into two functional regions, with differential methylation, known as imprinting centers (IC1 and IC2), that play a role in the development of BWS.

Loss of methylation at IC2 occurs in 50% of BWS patients, gain of methylation at IC1 occurs in 5% of BWS patients, CDKN1C mutations occurs in 10% of BWS patients; paternal uniparental disomy 11p15.5 (with abnormal methylation patterns of both IC1 and IC2), is present in 20% of BWS patients and finally duplication, inversion or translocation of 11p15.5 occurs in 1% of cases.

**Methods:** We present a case of a 32-year-old pregnant woman which fetal ultrasound at 20 weeks, revealed abdominal wall defect, gastroschisis, and short femur. Amniocentesis with aneuploidy screening, microarray-CGH and whole-exome-sequencing (WES), with bioinformatic analysis focused on our targeted skeletal dysplasia panel, were performed.

The clinical suspicion of BWS at 24 weeks led us to further molecular investigation. MS-MLPA kit ME030, from MCR-Holland, was performed to evaluate the methylation pattern of 11p15.5 region.

**Results and Discussion:** Aneuploidy screening, microarray-CGH and WES analysis revealed a normal result. MS-MLPA analysis revealed an abnormal methylation pattern at both IC1 (gain of methylation) and IC2 (loss of methylation). This result is compatible with paternal 11p15.5 uniparental disomy, with a very low recurrence risk. Our result confirmed the diagnosis of BWS and allowed us to identify the causal genetic mechanism.

Currently, ultrasound is a fundamental tool for the prenatal detection of affected cases. Molecular tests allowed us to confirm the diagnosis, to identify the causal genetic mechanism, to assess the recurrence risk, thus providing to be an excellent research tool, therefore complementing the diagnosis.

#### P83 - CONSTITUCIONAL MISMATCH REPAIR DEFICIENCY SYNDROME– A RETROSPECTIVE COHORT FROM A PORTUGUESE TERCIARY HOSPITAL

Ariana C. Mendes^1,3^, Inês R. Luz^2,3^, Maria Alice Carvalho^2,3^, Manuel Brito^2,3^, Joaquim de Sá^1,3^, Sofia Maia^1,3,4^, Jorge M. Saraiva^1,3,5^

^1^*Medical Genetics Unit, Hospital Pediátrico, Centro Hospitalar e Universitário de Coimbra, Coimbra, Portugal;* ^2^*Pediatric Oncology Unit, Hospital Pediátrico, Centro Hospitalar e Universitário de Coimbra;* ^3^*Clinical Academic Center of Coimbra, Coimbra, Portugal;* ^4^*University Clinic of Genetics, Faculdade de Medicina, Universidade de Coimbra;* ^5^*University Clinic of Pediatrics, Faculty of Medicine, University of Coimbra, Portugal.*

**Introduction:** Constitutional mismatch repair deficiency (CMMRD) is a rare pediatric cancer predisposition syndrome caused by biallelic germline variants in one of the mismatch repair genes (MLH1, MSH2, MSH6 and PMS2). Some phenotype-genotype correlations have already been established.

**Methodology:** Review of the clinical and molecular data of the patients with CMMRD diagnosed/evaluated in the Medical Genetics Unit of our institution in the last 23 years, comparing phenotype and genotype to those described in the literature.

**Case Reports**: Patient 1 – Female patient evaluated after the diagnosis of metastatic mucinous colorectal adenocarcinoma at the age of 17 years. Genetic studies identified two causal variants in the PMS2 gene, in compound heterozygous state, confirmed by segregation studies. She died at the age of 18.

Patients 2 and 3 – Two brothers with cafe-au-lait spots and the diagnoses of glioblastoma multiforme and anaplastic astrocytoma died at the ages of 9 and 10, respectively. Suspicion of CMMRD lead to investigation of their parents, which carried a heterozygous causal variant in the PMS2 gene. These results led to the post mortem likely diagnosis of CMMRD in the children.

Patient 4 – A five years old male patient with Coats disease and polydactyly underwent whole exome sequencing, which identified, as a secondary finding, a homozygous likely pathogenic variant in the PMS2 gene, establishing the molecular diagnosis of CMMRD. He also had cafe-au-lait spots. He is currently being followed by the Pediatric Oncology Unit, and there is no evidence of malignancy for the time being.

**Discussion:** Our data is in accordance with the literature, confirming the extreme severity and high mortality rate of CMMRD, and also corroborates the greater incidence of brain and Lynch syndrome associated malignancies, rather than hematological tumors, in CMMRD. We are currently collecting data from patients followed in other Portuguese centers, in order to have a bigger cohort, which may allow for further conclusions to be drawn.

#### P84 - WHOLE-EXOME-SEQUENCING (WES) – AN IMPORTANT TOOL TO ACCURATE DIAGNOSIS OF PRENATAL CONGENITAL ANOMALIES

Lisandra Castro^1^, Natália Salgueiro^1^, Ariana Conceição^1^, Marta Moreira^1^, Elsa Garcia^1^, Michael Freitas^1^, Adriana Gavina^1^, Cláudia Alves^2^, Cecília Correia^2^, Oana Moldovan^3^, Margarida R. Lima^1^

^1^*Unidade de Genética Molecular e Genómica-SynlabHealth Genética Médica, Porto;* ^2^ *Unidade de Citogenética-SynlabHealth Genética Médica, Porto;* ^3^ *Serviço de Genética-Centro Hospitalar de Lisboa Norte.*

**Introduction:** Prenatal WES is increasingly being used when karyotype and microarray are negative in pregnancies with congenital malformations (that represent 3% of cases and are associated with significant perinatal mortality and long-term morbidity).

Multiple congenital anomalies-neurodevelopmental syndrome (MCAND) is an X-linked recessive congenital multisystemic disorder that affecting the central nervous, cardiac, skeletal, craniofacial and genitourinary systems. This syndrome is characterized by distinct clinical features including poor growth, global developmental delay, short stature, impaired intellectual development, hypotonia, microcephaly, hydrocephalus, congenital heart defects and dysmorphic craniofacial features. Other characteristic features include ventriculomegaly and thin corpus callosum. The severity of this multisystemic disorder is extremely variable, ranging from death in early infancy to survival into the second/third decade.

**Methods:** 38-year-old pregnant woman, amniocentesis at 23 + 1 weeks, due to ventriculomegaly and short femur on US. Initially, karyotype and microarray-CGH analysis were performed. WES analysis was requested after TOP. Bioinformatic analysis was focused on the phenotype of the fetus.

**Discussion:** The karyotype and microarray-CGH analysis revealed a normal result. Due to the phenotype of the fetus and the advanced pregnancy stage, the couple decided to proceed with TOP. WES analysis was requested and a hemizygous variant in OTUD5 gene, c.667G>A (p.Ala223Thr) was detected. According to ACMG guidelines we classified this variant as Likely Pathogenic. The mother`s study revealed that the variant was “the novo”. The clinical information provided allowed us to conclude that this variant is compatible with X-linked multiple congenital anomalies-neurodevelopmental syndrome (OMIM#301056).

This result confirms that WES is an extremely efficient tool to study prenatal congenital malformations and very helpful to establish the definitive diagnosis and consequently leading to an increase in diagnostic rates. In this specific case, this diagnosis provided an useful information for guidance and family counselling for future pregnancies.

#### P85 - PHELAN–MCDERMID SYNDROME: A DE NOVO RING CHROMOSOME

Marta Souto^1^, Catarina Pinto^1^, Ana Matos^1^, Regina Arantes^1^, Márcia Martins^2^, Osvaldo Moutinho^3^, Rosário Pinto Leite^1^

^1^*Laboratório de Genética, Centro Hospitalar Trás-os-Montes e Alto Douro, Vila Real, Portugal;* ^2^ *Consulta de Genética, Centro Hospitalar de Trás-os-Montes e Alto Douro, Vila Real, Portugal;* ^3^ *Departamento da Mulher e da Criança, Centro Hospitalar Trás-os-Montes e Alto Douro, Vila Real, Portugal*

**Introduction:** The 22q13 deletion syndrome also known as Phelan–McDermid syndrome (PMS), is generally characterized by fetal hypotonia, global developmental delay, varied degrees of intellectual disability, normal to accelerated growth, severely delayed or absent speech, and minor dysmorphic traits. Autism and aggressive behaviors are present in some cases.

The authors present a patient with a de novo ring chromosome 22 and 22q13 deletion.

**Methodology:** 2-year-old boy referred to genetic consultation due to polymalformative syndrome, psychomotor development delay, hypotonia, hyperlaxity, deafness, VUR grade 4 with D-edge atrophy, peculiar facies. It was the first child of non-consanguineous couple, with apparent cognitive limitation.

Blood culture and cytogenetic analysis were performed according standards protocols. Fluorescence in situ Hybridization (FISH) technique with subtelomeric probe for chromosome 22 was applied. Array Comparative Genomic Hybridization (aCGH) was performed.

**Results:** Cytogenetics analysis revealed a 22q13 deletion in all metaphases analyzed. FISH technique confirmed that it was a ring. Parents karyotype was normal. aCGH technique revealed a 7,809Mbp deletion in 2q13.2q13.33 region, comprising SHANK3 and 110 more genes.

**Discussion:** The present case has a de novo ring chromosome 22 with 22q13 deletion. Terminal deletions at 22q13 are usually associated with the formation of ring chromosomes. In literature there are one hundred patients described with ring chromosome 22 and their clinical phenotype is similar to those with terminal 22q deletion.

The child has hypotonia, global developmental delay and minor dysmorphic features consistent with 22q13 deletion syndrome.

Despite the loss of 111 genes, the haploinsufficiency of SHANK3 gene is associated to the major neurological features that characterized 22q13 deletion. It has a role in synaptogenesis, in synaptic plasticity and in the regulation of dendritic spine morphology.

Every new case of a rare chromosomal alteration should be reported in order to obtain a more precise genotype/phenotype correlation, improving risk evaluation and genetic counselling.

#### P86 - SOMATIC MOSAICISM FOR APC PATHOGENIC VARIANT IN COLON POLYPOSIS: A CASE REPORT

Regina Arantes^1^, Marta Souto^1^, Jorge Lage^2^, Gabriela Soares^3^, Osvaldo Moutinho^4^, Rosário Pinto Leite^1^

^1^ *Laboratório de Genética, Centro Hospitalar de Trás-os-Montes e Alto Douro, Vila Real, Portugal;* ^2^ *Serviço de Gastrenterologia, Centro Hospitalar de Trás-os-Montes e Alto Douro, Vila Real, Portugal;* ^3^ *Consulta de Genética, Centro Hospitalar de Trás-os-Montes e Alto Douro, Vila Real, Portugal;* ^4^ *Departamento da Mulher e da Criança, Centro Hospitalar de Trás-os-Montes e Alto Douro, Vila Real, Portugal.*

**Introduction:** Clinical somatic mosaicism is the presence of a pathogenic variant in a gene occurring post-zigotically, usually causing an attenuated phenotype. Mosaic pathogenic variants in the APC gene are known to cause polyposis in patients presenting as single cases and it can preclude or delay the correct diagnosis. Confirmation of this rare diagnosis is important for the clinical follow-up of the patient and for risk information for relatives.

**Clinical case:** A 27 years-old female patient was referred for genetics consultation and testing for colonic polyposis. Both parents and the only brother are healthy, with no polyposis detected. The patient has one 8 years-old healthy daughter. Colonoscopy detected more than 60 adenomas, some with high grade dysplasia, mostly present in the sigmoid and rectum. Due to the large number of polyps, a total colectomy was performed, with no invasive adenocarcinoma detected. Upper digestive tract endoscopy was normal and no other relevant clinical features were present.

**Methods:** Blood sample was collected for genetic testing, and a NGS panel for polyposis genes was performed. NGS testing was performed in three separate adenomas.

**Results:** No pathogenic variant was detected in the blood sample. A pathogenic variant was detected in the APC gene in all three samples of adenomas: c.3880C>T (p.Q1294*), with allelic fractions of 52%, 63% and 38%. Additionally, the pathogenic variant c.712C>T (p.Q238*) was detected in one adenoma, with an allelic fraction of 6%. A review of the sequencing data of the blood sample confirmed that the c.3880C>T (p.Q1294*) was absent in all reads.

**Discussion:** Somatic mosaicism of pathogenic APC variants has been reported in the literature and should be suspected in patients that present with polyposis, no family history and a negative genetic test in a blood sample. If mosaicism is suspected, three separate adenomas should be tested, and if a pathogenic variant is found in at least two of those, mosaicism is confirmed. Absence of the variant in a blood sample indicates a very low risk that the variant is present in gonadal tissue, but testing of offspring should be discussed. This diagnosis is important for patient management and family counselling.

#### P87 - DETECTION OF LARGE GENOMIC REARRANGEMENTS IN BRCA1/BRCA2: A RETROSPECTIVE STUDY

Sónia Custódio^1^, Raquel Rodrigues^1^, Ana Medeira^1^, Juliette Dupont^1^, Patrícia Dias^1^, Márcia Rodrigues^1^, Mariana Soeiro e Sá^1^, André Travessa^1^, Raquel G. Silva^1^, Marta P. Soares^1^, Célia Carvalho^2^, Noélia Custódio^2^, Maria Carmo-Fonseca^2^, Ana Sousa^1^, Ana B. Sousa^1^

^1^ *Serviço de Genética Médica, Departamento de Pediatria, Hospital de Santa Maria, Centro Hospitalar Universitário Lisboa Norte, Lisboa, Portugal;* ^2^ *Instituto de Medicina Molecular João Lobo Antunes, Faculdade de Medicina, Universidade de Lisboa, Lisboa, Portugal*

**Introduction:** Acute myeloid leukemia (AML) is the most common acute leukemia in adults, with an annual rate of 4.3 new cases per 100.00. Characterized by immature myeloid cell proliferation and bone marrow failure, which can be subdivided into 9–11 pathogenetically different subtypes. Cytogenetic analysis plays an important role in the study of this pathology since the detection of specific chromosomal alterations are associated to subtypes and has prognostic and therapeutic implications. The most common cytogenetics abnormalities associated with AML are t(8;21)(q22;q22), inv/t(16)(p12q22) and t(15;17)(q22;q12).

**Clinical Report:** The authors present a case of a 68-year-old man with anemia, leukopenia with monocytosis and adenomegaly. The patient was admitted with suspect of AML. In peripheral blood sample, classical and molecular cytogenetics analysis were required to confirm the diagnosis.

Cytogenetic analysis revealed an unbalance translocation between the long arm of chromosome 1 (q21) and the short arm of chromosome 22 (p11), in 18 metaphases, which result in trisomy of long arm (q) of chromosome 1. Fluorescent in situ hybridization (FISH) analysis for t(15;17) and inv(16) had a normal pattern.

**Discussion and Conclusion:** The present case had an unbalanced translocation der(22)t(1;22)(q21;p11) resulting in trisomy of 1q. This is a rare event and usually detected as a secondary chromosomal abnormality in a complex karyotype. Recent studies have shown that the gain of 1q is a poor prognostic biomarker in acute leukemia associated to worse e survival and high risk.

The patient died two weeks after diagnosis with rapidly progressive disease, confirming the previous reports that trisomy 1q is associated with a poor prognosis.

To best our acknowledgment this is the first case reported with trisomy 1q involving the unbalanced translocation der(22)t(1;22)(q21;p10) in AML patients.

The present case highlights the importance of conventional cytogenetics, as it contributes to the diagnosis, prognostic and evaluation treatment of AML.

#### P88 - CARTILAGE-HAIR HYPOPLASIA DIAGNOSIS AFTER UNREVEALING WES STUDIES

Sónia Barros^1^, Inês Ribas^1^, Lisandra Castro^1^, Natália Salgueiro^1^, André Travessa^2^, Margarida Coucelo^3^, Janet Pereira^3^, Joana Azevedo^3^, Sérgio Sousa^4^, Margarida Reis-Lima^1^

^1^ *Unidade de Genética Molecular e Genómica - SynlabHealth Genética Médica, Porto;* ^2^ *Serviço de Genética - Centro Hospitalar de Lisboa Norte (CHLN);* ^3^ *UFHM - S. Hematologia Clínica - CHUC;* ^4^ *Serviço de Genética Médica- Hospital Pediátrico de Coimbra - CHUC*

**Introduction:** Cartilage-hair hypoplasia (CHH) is a rare pleiotropic autosomal recessive genetic disorder, characterized by disproportionate short stature, chest deformity, and increased risk of infections in infancy and childhood, among others. This disease is caused by pathogenic mutations in the RMRP gene, which encodes a 268bp untranslated RNA.

In this study, we present two case reports from patients who presented clinical characteristics of CHH whom whole exome sequencing (WES) failed to reveal a candidate variant.

**Methods:** The first patient is a seven-month-old female, with short-long-bones in PCT1. The second patient is a two-year-old male with immunodeficiency and slow weight gain.

Whole-exome sequencing (WES) was performed in both cases. WES bioinformatic analysis was focused on our 117 genes panel, associated with skeletal dysplasia.

Sanger sequencing for RMRP was performed in both cases using specific primers.

**Results:** In both cases WES analysis revealed a normal result. The clinical suspicion of CHH led us to further molecular investigation. Sanger sequencing for RMRP gene was performed.

Patient #1 presented the following variants: n.195C>T and n.-4_-23dupTACTCTGTGAAGCTGAGGAC, both classified as pathogenic and described in the literature.

Patient #2 presented the following variants: n.+6T>C and n.-5_-26dupTACTACTCTGTGAAGCTGAGAA, also described and classified as pathogenic in the literature.

The variants found in both cases were compatible with the clinical phenotype of the patients and confirm the diagnosis of CHH.

**Discussion:** It is essential to note that there are still a significant percentage of cases in which WES analysis leads to false negative results, for several reasons as part of its own technical limitations.

Therefore, it is crucial to clearly characterize the clinical phenotype of the studied patients and insist in studying with other techniques. In these two cases the use of Sanger sequencing allowed us to confirm the genetic diagnosis of the patients and provide a correct genetic counselling to the family. Clinical judgment is crucial to lead the laboratory investigations.

#### P89 – PERMISSION TO PUBLISH NOT GRANTED BY THE AUTHORS

#### P90 - THE IMPORTANCE OF KARYOTYPING IN UNRAVELING A CHROMOSOME 15 DUPLICATION RING

Paula Oliveira^1^, Isaltina França^1^, Manuela Mota Freitas^1,2,3^, Cristina Candeias^1,2,3^, Megan Braga^1^, Ana Miguel Capela^4^, Maria Abreu^4^, Cláudia Falcão Reis^4^, Natália Oliva-Teles^1,2,3,5^

^1^*Serviço de Genética Laboratorial, Clínica de Genética e Patologia, Centro Hospitalar Universitário de Santo António (CHUdSA), Porto, Portugal;* ^2^*UMIB – Unidade Multidisciplinar de Investigação Biomédica, ICBAS – Instituto de Ciências Biomédicas Abel Salazar, Universidade do Porto, Porto, Portugal;* ^3^*ITR – Laboratory for Integrative and Translational Research in Population Health, Porto, Portugal*;^4^Serviço de Genética Médica, Centro de Genética Médica Jacinto Magalhães, Centro Hospitalar Universitário de Santo António, Porto, Portugal;^5^MEDCIDS – Departamento Medicina da Comunidade, Informação e Decisão em Saúde, Faculdade de Medicina, Universidade do Porto, Porto, Portugal.

**Introduction:** The most frequent region of chromosome 15 involved in CNVs is 15q11q13, which includes Prader-Willi/Angelman Syndrome critical region (PWACR). This region is highly susceptible to genomic rearrangements including interstitial deletions, duplications and the formation of small supernumerary chromosomes (SMCs) such as rings. Chromosome 15q11-q13 duplication syndrome (OMIM #608636) has variable expressivity, and the duplicated segment’s size, level of mosaicism, tissues where it is expressed and parental origin influence severity.

**Methodology:** We report a 10 yo boy with attention deficit hyperactivity disorder (ADHD), mild learning difficulties, tall stature and macrocephaly. Psychometric evaluation excluded intellectual disability. Brain MRI showed myelinization delay. He was referred to our laboratory for conventional/molecular cytogenetics studies to assess a 10,29 Mb 15q11.2q13.3 duplication reported on a previous microarray-CGH. Chromosome GTL banded metaphases analysis was done in peripheral blood cultures according to standard methods and FISH analysis was performed using UBE3A (Cytocell) probe in lymphocytes and in buccal swab sample.

**Results:** Both karyotype and FISH (blood lymphocytes and buccal swab, respectively), revealed two cell lines – one with a ring chromosome and another 46,XY. Blood lymphocytes: mos 47,XY,+r(15)[16]/46,XY[14].ish r(15)(UBE3A+) Buccal swab: nuc ish(UBE3A x3)[24]/(UBE3A x2)[76]

**Discussion:** Our patient had a milder phenotype than previous reports of 15q11.2q13.3 Duplication Syndrome, prompting further investigation. Conventional cytogenetic analysis showed that the chromosome 15 duplicated material was a small supernumerary ring of chromosome 15 and revealed a true mosaicism in this patient. Mosaicism often poses a challenge in phenotypic determination, and in this instance it likely contributed to our patient’s milder presentation. This report shows that karyotype and FISH analysis can play an important role in unravelling a duplication’s nature, clarifying microarray-CGH results, and allowing more accurate genetic counselling.

#### P91 - CTNNA1 PATHOGENIC VARIANT DETECTED IN A PATIENT WITH LOBULAR BREAST CANCER: CASE REPORT

Michael Freitas^1^, Maria Lopes de Almeida^2^, Lisandra Castro^1^, Natália Salgueiro^1^, Ariana Conceição^1^, Ana Correia^1^, Marcelo Dantas^1^, João Mata^1^, Adriana Gavina^1^, Cristiana Ferreira^1^, Margarida R. Lima^1^

^1^ *Unidade de Genética Molecular e Genómica-SynlabHealth Genética Médica, Porto;* ^2^ *Unidade de Genética Médica-Hospital de Braga*

**Introduction:** Hereditary diffuse gastric cancer (HDGC) is characterised by early onset diffuse gastric cancer (DGC) and lobular breast cancer (LBC). The CTNNA1 gene, has been recently classified as HDGC predisposing gene, encoding for α-E-catenin, which is a protein that interacts with E-cadherin in the “adherens junction complex”. However, the associated causality between variant-type and the tumor spectrum in CTNNA1 variants patients is yet unclear.

**Case Report:** A 58-year-old female with lobular breast cancer (LBC) and family history of gastric cancer (father and uncles). The patient was referred to our Center to perform the multigene panel for Hereditary Breast and Ovarian Cancer (HBOC) risk. As we included recently in our panel the CTNNA1 gene, a frameshift variant c.2617_2620del (p.Glu873fs) was detected. This variant is not described in population databases, disease databases or in the literature consulted. According to ACMG recommendations this variant is classified as likely pathogenic.

**Discussion:** Pathogenic variants in CTNNA1 are described as being involved in gastric cancer and lobular breast cancer. This case report demonstrated, the importance to investigate the presence of variants in CTNNA1 in families with gastric cancer and/or lobular breast cancer. Moreover, current knowledge supports considering CTNNA1 variants as clinically actionable in HDGC carrying families.

The use of extended multigene panels is currently a strong tool in Oncology. Testing for more cancer risk genes become increasingly important for better characterization of the associated clinical phenotypes and cancer epidemiology.

#### P92 - UNEXPECTED RESULTS IN AML: CONVENTIONAL CYTOGENETICS REMAINS IMPORTANT

Ana Matos^1^, Catarina Pinto^1^, Regina Arantes^1^, Marta Souto^1^, Manuel Cunha^2^, Osvaldo Moutinho^3^, Rosário Pinto-Leite^1^

^1^ *Laboratório de Genética, Centro Hospitalar Trás-os-Montes e Alto Douro (CHTMAD);* ^2^ *Serviço de Hematologia, CHTMAD, Vila Real, Portugal;* ^3^ *Departamento da Mulher e da Criança, CHTMAD, Vila Real, Portugal.*

**Introduction:** Acute myeloid leukemia (AML) is a malignant neoplasm characterized by the clonal expansion of stem cells of the myeloid lineage. It exhibits a wide range of subgroups, each of which may be link to distinct genetic and molecular alterations, and has a varying prognosis. The authors present three suspected AML cases with rare chromosomal abnormalities.

**Methodology:** Three bone marrow cell cultures and GTL banding were performed. Conventional cytogenetic analysis followed the standard cytogenetic guidelines.

**Results:** Cytogenetic analysis revealed, in case 1, monosomy on chromosome 7 and a reciprocal translocation between the long arms of chromosomes 11 (q13) and 21 (q22).

In case 2, a translocation was observed between the long arms of chromosomes 19 (q13) and 21 (q22). A sole trisomy of chromosome 14 were observed in case 3.

**Discussion:** The cytogenetic alterations detected, in the three cases, despite being associated with AML, are rare.

Case 1 and case 2 presents a translocation involving the 21q22 region, respectively, t(11;21)(q13;q22) and t(19;21)(q13;q22). In literature, these two translocations are described in a total of 5 cases, all with AML diagnosis and poor outcome. Translocations on chromosome 21q22 are associated with a poor prognosis, as they may involve the RUNX1 gene, which have an important role in critical cellular processes and leukemogenesis. A monosomy of chromosome 7 was also detected in case 1. This monosomy is seen in AML and is associated with a poor prognosis.

The trisomy 14, detected in case 3, is a rare event with only 18 cases described at the literature. Its prognosis still unclear, although some authors referred that is associated with aggressive disease course and is found at initial diagnosis, it may represents an early event in leukomagenesis.

In the three cases presented, the cytogenetic abnormalities detected justify the poor prognosis of these patients associated with AML, highlighting the extremely importance of cytogenetic analysis, not only confirms the diagnosis but it also offers critical information regarding to the prognosis and therapeutic strategy.

#### P93 - WHOLE EXOME SEQUENCING- FIRST TIER TEST FOR FETUSES WITH SEVERE CENTRAL NERVOUS SYSTEM ANOMALIES

Natália Salgueiro^1^, Ariana Conceição^1^, Lisandra Castro^1^, Elsa Garcia^1^, Ana Correia^1^, Cláudia Alves^2^, Fernando Santos^3^, Nuno Pereira^3^, Isabel Cerveira^3^, Dulcina Lopes^4^, Adosinda Rosmaninho^5^, Maria Lopes de Almeida^5^, Lina Ramos^6,7^, Fabiana Ramos^6,7^, Margarida R. Lima^1,2^

^1^*Unidade de Genética Molecular e Genómica-SynlabHealth Genética Médica, Porto;* ^2^*Unidade de Citogenética-SynlabHealth Genética Médica, Porto;* ^3^*Unidade de Medicina fetal- DPN, CHTV;* ^4^*Hospital de Cascais, Unidade de Obstetrícia, Lisboa;* ^5^*Serviço de Ginecologia/Obstetrícia, DPN, Hospital Senhora da Oliveira - Guimarães, EPE;* ^6^*Unidade de Genética Médica-Hospital Pediátrico, Centro Hospitalar e Universitário de Coimbra, Coimbra;* ^7^*Centro de Diagnóstico Pré-Natal Maternidade Bissaya Barreto*

**Introduction:** Approximately 2–5% of pregnancies present with sonographically detected fetal anomalies that warrants further evaluation. The central nervous system (CNS) is affected in 9% of fetal malformations but accounts for around one‐third of all termination of pregnancies (TOP) due to fetal anomalies. Moreover, CNS anomalies are often diagnosed in an advanced stage of pregnancy.

Next‐generation sequencing technology, such as targeted multigene panels or Whole Exome sequencing (WES) is sometimes used as a second‐or third‐option diagnostic test in selected cases of CNS fetal anomalies. Recent studies have shown that WES provides an additional rate of 5–57% over that of chromosomal microarray analysis (CMA) in prenatal setting.

In this study, we retrospectively studied 21 cases referred to our center for genetic evaluation due to CNS anomalies. Five of these cases were evaluated after TOP and 19 were ongoing pregnancies.

**Methods:** All fetuses were referred to our center for genetic evaluation. WES analysis were performed in all cases. Bioinformatic analysis was focused on our 59 gene panel for congenital anomalies of nervous system and mainly on the targeted phenotype. In all cases the bioinformatic analysis included copy number variants (CNVs).

**Results:** In 28,7% (6/21) of the cases, our study identified a genetic aetiology for the fetus phenotype. Of these 6 diagnosed cases, 4 were “de novo” autosomal dominant (AD) condition, 1 revealed an inherited autosomal recessive (AR) condition, and the last one was a X-linked disease.

**Conclusions**: In our study, WES provided a high diagnostic rate (around 30%) in fetuses with CNS anomalies. These results demonstrated that an accurate identification of the ultrasound detected anomalies is crucial for a successful diagnosis. These data suggest that WES may be considered a major tool for the prenatal diagnosis of fetal CNS anomalies. This is of particular importance given the time constraints of an ongoing pregnancy and the risk of recurrence in future pregnancies.

#### P94 - TREACHER COLLINS SYNDROME: CLINICAL AND MOLECULAR INSIGHTS FROM A RETROSPECTIVE STUDY

Joana Adelaide Catanho^1^, Susana Lemos Ferreira^1^, Mafalda Melo^1^, Sofia Nunes^1^, Diana Antunes^1^, Márcia Rodrigues^2^, Rui Gonçalves^1^, Teresa Kay^1^, Margarida Venâncio^1^, Inês Carvalho^1^

^1^*Serviço de Genética Médica, Hospital Dona Estefânia, Centro Hospitalar Universitário Lisboa Central, Lisboa, Portugal;* ^2^ *Serviço de Genética Médica, Departamento de Pediatria, Hospital de Santa Maria, Centro Hospitalar Universitário Lisboa Norte, Lisbon, Portugal*

**Introduction:** Treacher Collins syndrome (TCS) is a rare mandibulofacial dysostosis (MFD) caused by pathogenic variants in TCOF1 (#154500); POLR1D (#613717); POLR1C (#248390) or POLR1B (#618939) genes, involved in ribosomal RNA transcription.

Clinical features include facial bones hypoplasia, external ear anomalies, hearing loss, downward palpebral fissures, and colobomas. It is associated with incomplete penetrance and variable expression, so misdiagnosis may occur. Some patients remain without a known genetic anomaly.

**Objectives and Methods:** Retrospective review of clinical and molecular data from 1) patients with clinical suspicion of TCS or 2) diagnosed cases of TCS, in our outpatient Genetic Department, ranging from 2004-2023.

**Results:** In total, we identified 11 patients. From 1), 9 cases had initial suspicion of TCS. Of these, 4 were diagnosed with TCS (TCOF1-3; POLR1D-1); 1 with Burn-McKeown Syndrome (TXNL4A); 4 remained without molecular diagnosis and did not return for follow-up. From 2), 1 patient was diagnosed due to deafness molecular investigation (POLR1D); a father with milder phenotype was identified after segregation studies (TCOF1).

Common features in this case series were malar/zygomatic hypoplasia (9/11); micrognathia (9/11); retrognathia (8/11); conductive hearing loss (8/11); downward palpebral fissures (8/11); lower eyelid coloboma (7/11), partial absence of lower eyelashes (7/11); microtia (6/11); cleft palate (6/11). In patients without molecular diagnosis, all presented malar/zygomatic hypoplasia, micro-retrogathia and 3 had hearing loss. Only 1 case had reported prenatal findings.

To the best of our knowledge, we identified 3 new variants: 2 frameshift and 1 splice-site in TCOF1.

**Conclusions**: Phenotypic findings are consistent with literature, yet common features are not exclusive to TCS. We identified 2 cases with milder characteristics, evidencing phenotypic variation associated with TCS. These findings enhance the importance of genetic diagnosis in MFD, for proper counseling and management. Timely revision of previous undiagnosed cases is advisable. We contributed to expand molecular data, adding 3 new TCOF1 variants not previously reported.

#### P95 - LATE-ONSET HEREDITARY SPASTIC PARAPARESIS TYPE 11 (SPG-11) – A PHENOTYPIC RE-INTERPRETATION OF A KNOWN PATHOGENIC VARIANT

Leonor Dias^1,2^, Mafalda Seabra^1,2^, Joana Guimarães^1,2^, Ana Grangeia^2,3^, Miguel Leão^3^

^1^ *Neurology Department, Centro Hospitalar Universitário de São João, E.P.E.;* ^2^ *Medical Faculty, Porto University;* ^3^ *Human Genetic Department, Centro Hospitalar Universitário de São João, E.P.E.*

**Introduction:** Hereditary spastic paraparesis type 11 (SPG-11) is an autosomal recessive complicated spastic paraparesis with usual onset in adolescence or before 30 years of age. It presents a clinical course that can include urinary incontinence, cognitive impairment, peripheral nerve involvement, and parkinsonism. Atypical forms are described, but rarely present in advanced age. We present a consanguineous family with 3 affected siblings presenting a late-onset SPG-11.

**Clinical Case:** We describe a family with three affected siblings: a 51-year-old male, a 58-year-old female, and a 60-year-old female. All the affected members presented gait impairment starting in the fifth decade of life (41- 57 years), with neurological examination revealing spastic paraparesis with lower limb hyperreflexia. In addition to spastic paraparesis, other systems were involved, namely urinary dysfunction, extrapyramidal signs, peripheral nerve involvement, non-amnestic mild cognitive impairment, and pseudo-bulbar involvement. Brain MRI revealed a thin corpus callosum. No patient had a history of visual impairment. Clinical exome sequencing of the index patient revealed a splicing pathogenic variant c.6477 + 4A>G(r.spl?) in homozygosity in the SPG11 gene. All affected members of this family harbored the same pathogenic variants thus confirming SPG-11 diagnosis.

**Discussion:** Late-onset SPG-11 is an atypical presentation, leading to an important delay until evaluation by a neurologist and delaying diagnosis. It is more often associated with missense and splice site variants. The pathogenic variant c.6477 + 4A>G (r.spl?) had been previously described in compound heterozygosity, associated with the pathogenic missense variant c.1282A>T, in exon 6), in a family with a more severe early-onset SPG-11 phenotype (Stevanin et al 2008). Our work expands the clinical phenotype of this splice site variation.

#### P96 - A NOVEL KDM2B VARIANT ASSOCIATED WITH NEURODEVELOPMENT DISORDER – A CASE REPORT

Inês C. Santos^1^, João Parente Freixo^2^, Margarida Venâncio^1^, Diana Oliveira Antunes^1^

^1^ *Serviço de Genética Médica, Hospital Dona Estefânia, Centro Hospitalar Universitário Lisboa Central, Lisboa, Portugal;* ^2^ *Centro de Genética Preditiva e Preventiva, Instituto de Biologia Molecular e Celular, Instituto de Investigação e Inovação em Saúde, Universidade do Porto, Porto, Portugal.*

**Introduction:** KDM2B is a protein coding gene known to be involved in numerous biological processes, encompassing the regulation of the cell cycle, metabolic regulation and DNA-damage repair. Consistent with its critical role as an epigenetic regulator and transcriptional control, KDM2B has been described as a regulator of cellular differentiation, thus associated with cancer. While its functions are well-known, its association with neurodevelopmental disorders is only recently being established.

**Case Report:** We report a 25-year-old (yo) female, first referred to our outpatient clinic at the age of 12 due to global development delay and facial dysmorphisms. She is the second child of non-consanguineous parents, born at 39 weeks of gestation – birth weight at the 24th centile, length at 83rd centile and head circumference at 23rd centile. Pregnancy, pre- and perinatal history was unremarkable. Global developmental marked by delayed acquisition of motor skills, independent walking achieved at 6yo and speech delay, uttering first words after her third birthday and comprehensive phrases at 6yo. At observation, coarse facial features, prominent nose with broad nasal tip and short philtrum. Further diagnosed with moderate intellectual disability (ID), auto and hetero-aggression, hyperactivity and sleep disturbances. Brain MRI showed dyskinesia of the corpus callosum, metabolic screening within normal ranges. Karyotype, 22q.12 and 17p11.2 FISH, Array Comparative Genomic Hybridization (ACGH) 60K, ACGH 750K and target gene panel for ID, came back normal or non-correlated with the phenotype. Recently, whole-exome trio analysis – patient and healthy parents – was preformed and revealed a de novo heterozygous likely pathogenic variant in KDM2B gene - c.3911dup (p. (Glin1305Alafs*8)).

**Discussion:** The association between KDM2B and neurodevelopmental disorders is a relatively new field of study. Only recently, a novel syndrome caused by heterozygous KDM2B variants clinically associated to ID has been delineated. We describe a novel variant that adds on to this knowledge, deepening our understanding about the genotype-phenotype correlation with this gene variants.

#### P97 - DISTAL TRISOMY 6Q: A RARE GENETIC CONDITION DETECTED AT PRENATAL DIAGNOSIS

Cláudia Alves^1^, Joana Trindade^1^, Diana Martins^2^, João Paulo Marques^2^, Mafalda Lopes^1^, Cristina Perez^3^, Maria João Oliveira^1^, Ana Azeredo^1^, Natália Salgueiro^4^, Cecília Correia^1^, Margarida Reis Lima^1,4^

^1^ *Unidade de Citogenética-SynlabHealth Genética Médica, Porto;* ^2^ *Hospital Beatriz Ângelo, Serviço de Ginecologia/Obstetrícia, Lisboa;* ^3^ *Unidade de Citogenética e Arrays, SYNLAB Diagnósticos Globales S.A.U.;* ^4^ *Unidade de Genética Molecular e Genómica-SynlabHealth, Genética Médica, Porto*

**Introduction:** Distal trisomy 6q is a rare chromosomal anomaly syndrome resulting from the partial duplication of the long arm of chromosome 6, with highly variable phenotype, typically characterized by growth and developmental delay, intellectual disability and craniofacial dysmorphism. Other features reported include cardiac, urogenital, ophthalmologic and hand and foot anomalies, as well as umbilical hernia, spasticity, and seizures (ORPHA:96098).

Ultrasonographic findings in reported cases involving distal 6q duplications are limited and vary between patients. Fetal abnormalities include microcephaly, flat nasal bridge, cardiovascular anomaly, and a single umbilical artery.

**Methods:** A 19-years-old primigesta was referred at 13 weeks of gestation due to fetal cystic hygroma, reversed a-wave in the ductus venosus and single umbilical artery.

Multiplex-PCR for screening common aneuploidies and microarray-CGH were performed.

At 18W+5D gestation, the fetus had cystic hygroma and hyperechogenic kidneys. Amniotic fluid (AF) sample was received for confirmatory microarray-CGH test. Fetal echocardiogram at 21W+6D showed an apparently isolated left superior vena cava, systemic venous return with coronary sinus dilation.

**Results and Discussion:** Array analysis from uncultured CVS showed a mosaic copy number gain of 72.2 Mb at chromosome 6q16.1q27, classified as pathogenic.

Around 19 weeks of gestation, amniocentesis was performed and microarray-CGH confirmed the 6q16.1q27 gain (non-mosaic state). Subsequently, karyotype analysis of the AF revealed a structural abnormality, where an additional segment 6q16.1q27 located at the terminal band of the short arm of one chromosome 6 was observed.

Parental karyotypes were suggested to exclude a chromosomal rearrangement and help estimate the recurrence risk. The couple refused autopsy and anatomopathological investigations.

Few terminal 6q duplication cases are described in the literature, specially at prenatal diagnosis, which difficult the correlation between this genetic abnormality and ultrasound findings.

Conventional karyotype in this case also contributed with valuable information for future counselling and genetic guidance.

#### P98 - THE ROLE OF GENETIC TESTING IN THE DIAGNOSIS OF CILIOPATHIES

Rafael Graça^1^, Diana Antunes^1,2,3^, Catarina Silveira^1^, Yuri Chiodo^1^, Maria Carmo-Fonseca^4^

^1^ *GenoMed - Diagnósticos de Medicina Molecular, S.A, Lisboa, Portugal*, ^2^ *Santa Marta Hospital, Medical Genetics Department, Lisboa, Portugal*, ^3^ *Dona Estefânia, Medical Genetics Department, Lisboa, Portugal*, ^4^ *Instituto de Medicina Molecular João Lobo Antunes, Faculdade de Medicina da Universidade de Lisboa, Portugal*

**Introduction:** TCiliopathies comprise a group of disorders associated with genetic variants, which result in either abnormal formation or function of cilia. These disorders can affect multiple organ systems and are primarily inherited as an autosomal recessive trait. Primary ciliary dyskinesia (PCD) is a common ciliopathy that affects the function of cilia in the respiratory and reproductive systems, being clinically characterised by recurrent infections, bronchiectasis and in, some cases, infertility.

**Methods:** Retrospective observational analysis of genetic testing for suspected ciliopathies/PCD from May 2020 to September 2023. For diagnosis, two gene panels were used: Primary Ciliary Dyskinesia panel (n=41) and Ciliopathies panel (n=176). The mean age of the 25 patients tested was 10 years old. The focus of the study was to identify the diagnostic success of gene panels in clinical practices.

**Results:** Out of the total patients, 24% (n=6) were given a clear and conclusive genetic diagnosis, characterized by the identification of either homozygous or compound heterozygous pathogenic (P) or likely pathogenic (PP) variants. Three patients have a presumable diagnosis with one P/PP variant identified and one variant of uncertain significance (VUS) with a clear tendency for pathogenicity. Six additional patients have an inconclusive diagnosis with only one P/PP variant identified and/or low impact VUS. The remaining ten patients received a negative genetic diagnosis. PP/P variants we identified in genes CCDC103, CEP164, DNAH5, DNAH9, DRC1, HYDIN, NPHP1 and RSPH1.

**Discussion:** Variant RSPH1 (NM_080860.4): c.275-2A>C was the most common cause of disease, identified in homozygosity in three patients. The definitive diagnosis ratio for a more focused gene panel (PCD panel) and a larger panel (Ciliopathies panel) are 20% (3/15) and 30% (3/10), respectively. Despite the complex aetiology of ciliopathies with monogenic, digenic and oligogenic forms, genetic testing is an effective method for providing a conclusive diagnosis, complementing other diagnostic techniques such as nasal nitric oxide high-speed video microscopy or transmission electron microscopy. While there is currently no cure for ciliopathies, as the clinical features associated with certain genotypes can vary greatly, early diagnosis and tailored therapies can improve the quality of life for affected individuals. This work aims to increase awareness about the far-reaching effects of genetic testing and the importance of data sharing for result interpretation.

#### P99 - STILLBIRTH CASE REPORT ASSOCIATED WITH A PATERNALLY INHERITED VARIANT IN MAGEL2 GENE

Maria S. Nóbrega^1^, Cláudia Falcão Reis^2,3,4^, Vânia Machado^2^, Marisa Teixeira^1^, Joaquim Sá^1,5^, Rita Cerqueira^1^

^1^*1 CGC Genetics Unilabs;* ^2^ *Consulta de Genética, Hospital de Santo Espírito da Ilha Terceira;* ^3^ *Serviço de Genética, Centro Hospitalar Universitário de Santo António;* ^4^ *Instituto de Investigação em Ciências da Vida e da Saúde, Escola de Medicina, Universidade do Minho*, ^5^ *Serviço de Genética Médica, Centro Hospitalar e Universitário de Coimbra*

**Introducion:** Schaaf-Yang syndrome is a rare multisystem disorder, with similarities to Prader-Willi syndrome, with variable profile and severity. It is caused by heterozygous truncating variants in the maternally imprinted MAGEL2 gene. The presence of paternally inherited variants in MAGEL2 gene has already been associated with fetal demise.

**Methodology:** We present a case of a female stillbirth after a 39 weeks pregnancy from a couple with a prior healthy child and no significant personal or family clinical history. All fetal sonographies were normal as were the posterior anatomopathology studies. Fetal array CGH study was negative and a trio whole exome analysis was requested. Whole exome sequencing was performed at Illumina Novaseq 6000® and the resulting data was processed and analyzed with an in-house bioinformatics pipeline and NGS analysis software.

**Results:** Single nucleotide variant (SNV) analysis detected the frameshift variant NM_019066.5: c.37_38insAC p.(Pro13Hisfs*18), in heterozygosity, in exon 1 of 1, in MAGEL2 gene. The variant was also present in the father. Variant presence was confirmed through Sanger sequencing for both fetal and paternal samples. The detected variant is not described in gnomAD, however is present in ClinVar (ID: 435801) as a variant of unknown significance. According to ACMG criteria the classification is unknown significance. The variant is absent from controls in gnomAD (PM2supporting) and is a frameshift that escapes NMD located in a critical region for protein function (PVS1strong).

**Discussion:** MAGEL2 gene is associated with Schaaf-Yang syndrome [MIM 615547], an autosomal dominant disorder that can skip generations if the mutation is on the maternal chromosome. This clinical case recalls the relevance of trio whole exome sequencing for etiological diagnosis, significantly surpassing array CGH, and proposes a link between a case of a stillbirth with no morphologic anomalies and a possible Schaaf-Yang syndrome. Additionally, the identification of a truncating variant inherited from the father could eventually be relevant in the future for the family genetic counseling.

